# Supplementary material for: Isolable radical cation and dication of dialumene
Source: Nat Commun. 2026 Feb 18;17:1937. doi: 10.1038/s41467-026-69607-6 (PMC12923815; doi:10.1038/s41467-026-69607-6)
Supplement: Supplementary file 1 — Supplementary Information [file 41467_2026_69607_MOESM1_ESM.pdf]

Supplementary Information

Isolable Radical Cation and Dication of Dialumene

Xufang Liu, Arseni Kostenko, Eva Körber, Huaiyuan Zhu, Karsten Meyer and Shigeyoshi Inoue*

Contents

1. Supplementary Methods 3

1.1 General Methods and Instrumentation 3

1.2 Synthesis and Characterization 5

1.2.1 Synthesis of $I^{i}Pr_2Al(SiTMS_3)I_2$ 5

1.2.2 Synthesis of 1 7

1.2.3 Synthesis of 2 10

1.2.4 Synthesis of 3 12

1.2.5 Synthesis of 4a 14

1.2.6 Synthesis of 4b 17

1.2.7 Synthesis of 5 17

1.2.8 Synthesis of 6 20

1.2.9 Synthesis of 7 23

1.2.10 Synthesis of 8 24

1.2.11 Synthesis of 9a 27

1.2.12 Synthesis of 9b 31

1.3 Electrochemical Measurements on Dialumene 1 34

1.4 Redox-Reversible Transformation 39

1.4.1 Synthesis of 2 via the reduction of 3 39

1.4.2 Synthesis of 1 via the reduction of 2 or 3 39

1.4.3 Synthesis of 2 via the reaction of 1 and 3 40

1.5 Reactivity of Radical Cation 2 40

2 Single Crystal X-Ray Structure Determination 41

3 Computational Details 52

4 Supplementary References 56

1. Supplementary Methods

1.1 General Methods and Instrumentation

All experiments and manipulations were carried out under argon atmosphere using standard Schlenk or glovebox techniques. The glassware was heat-dried under vacuum prior to use. All glass junctions were coated with PTFE-based grease Merkel Triboflon III. For stirring, PTFE-coated magnetic stirrer bars were used or glass-coated ones if stated. Liquid phases were transferred using standard PE/PP syringes equipped with stainless steel cannula or directly canted from vessel to vessel if not stated otherwise. Solvents were dried by standard methods (withdrawal from MBraun Solvent Purification System and storage over molecular sieves, or distilled from sodium/ benzophenone or CaH_2 under argon atmosphere and degassed via freeze-pump-thaw cycling). All chemicals were purchased from commercial suppliers and used as received if not stated otherwise. Deuterated solvents were obtained from Deutero Deutschland GmbH and were dried over molecular sieves.

All NMR samples were prepared under argon in J. Young PTFE tubes. NMR spectra were recorded on a Bruker AV400US, DRX400, AVHD300 and AV500cr at ambient temperature (300 K) if not stated otherwise. ^{1}H and ^{13}C NMR spectra were calibrated against the residual proton and natural abundance carbon resonances of the respective deuterated solvent as internal standard.

Quantitative elemental analyses (EA) were carried out using a EURO EA (HEKA tech) instrument equipped with a CHNS combustion analyzer at the Laboratory for Microanalysis at the TUM Catalysis Research Center.

The UV-vis spectra were taken on an Agilent Cary 50 spectrophotometer with a Schlenk quartz cuvette at the Central Analytic Department at the TUM Catalysis Research Center.

EPR spectra were recorded on a JEOL continuous-wave spectrometer JES-FA200, equipped with an X-band Gunn diode oscillator bridge, a cylindric mode cavity, and a helium cryostat. The spectra shown were measured using the following parameters: microwave frequency = 8.944 GHz, modulation amplitude 1.0 mT, microwave power 1.0 mW, modulation frequency 100 kHz, time constant of 0.1 s. Data analysis and simulation was performed using the software “eview” and “esim”, written by Dr. Eckhard Bill (MPI CEC, Mülheim/Ruhr),[1,2] based on a Spin-Hamiltonian description of the electronic ground state:

$$\hat{H} = D\left(\hat{S}_z^2 - \frac{1}{3}S(S+1) + \frac{E}{D}\left(\hat{S}_x^2 - \hat{S}_y^2\right)\right) + \mu_B\, \underline{g}\, \vec{S}.$$

Here, S represents the total spin quantum number of the coupled system, D and E/D are the axial and rhombic zero-field parameters, respectively, and $\underline{g}$ is the g-matrix. Calculations are based on the S = 5/2 routines developed by Gaffney and Silverstone.[3] EPR line widths, W, are given in units of mT at full-width-half-maximum (FWHM).

Electrochemical measurements were carried out at room temperature under dinitrogen atmosphere with an *µ*Autolab Type III potentiostat. Samples were recorded in 1 mM solutions of the analyte in tetrahydrofuran containing 0.1 M electrolyte (≥99.0% for electrochemical analysis, purchased from Sigma Aldrich and used without further purification) using a rotating disk electrode with a glassy carbon (3 mm diameter) as working electrode and platinum wires as counter and pseudo-reference electrodes. Ferrocene (Fc) was added as an internal standard and all measurements were referenced to the Fc^+/Fc couple.

1.2 Synthesis and Characterization

1.2.1 Synthesis of I^{i}Pr$_2$Al(SiTMS$_3$)I$_2$

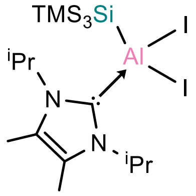

TMSI (2.4 g, 12 mmol) was added dropwise to a stirring solution of I^{i}Pr·AlH$_3$ (0.84 g, 4 mmol) in 40 mL toluene via cannula at 0°C. After 10 minutes the ice bath was removed and stirring was continued at room temperature for 1 h followed by heating to 40 ºC for another 3 h to ensure complete conversion to I^{i}Pr·AlI$_3$. A dropping funnel, containing 50 mL toluene solution of KSiTMS$_3$ (1.14 g, 4 mmol), was connected to this flask which was added dropwise over 2 hours to the in situ generated solution of I^{i}Pr·AlI$_3$ at –78 ºC. After addition, the solution was stirred at –78°C for 1h, followed by stirring at room temperature overnight. The solution was evaporated to dryness and the residue was washed with pentane (2 × 20 mL). The crude product was extracted with toluene (3 × 40 mL). Finally, I^{i}Pr$_2$Al(SiTMS$_3$)I$_2$ was obtained as a colorless solid from the concentrated solution of toluene at –30 °C overnight (1 g, 35% yield).

^{1}H NMR (400 MHz, C$_6$D$_6$): δ [ppm] 5.73 (hept, J = 6.9 Hz, 2H, C*H*(CH$_3$)$_2$), 1.46 (s, 6H, *CH$_3$* NHC), 1.21 (d, J = 6.9 Hz, 12H, CH(C*H$_3$*)$_2$), 0.61 (s, 27H, SiC*H$_3$*).

^{13}C{^{1}H} NMR (101 MHz, C$_6$D$_6$): δ [ppm] 127.39 (*C*=*C* NHC), 53.11 (*C*H(CH$_3$)$_2$), 22.14 (CH(*C*H$_3$)$_2$), 10.08 (*C*H$_3$ NHC), 4.48 (Si*C*H$_3$). No signal was found for aluminum-bonded carbene carbon atom, due to the quadrupolar momentum of the ^{27}Al nucleus.

^{29}Si NMR (79 MHz, C$_6$D$_6$): no signal found.

Elemental Analysis (%): Calcd: C 33.89, H 6.68, N 3.95; Found: C 33.93, H 6.54, N 3.82.

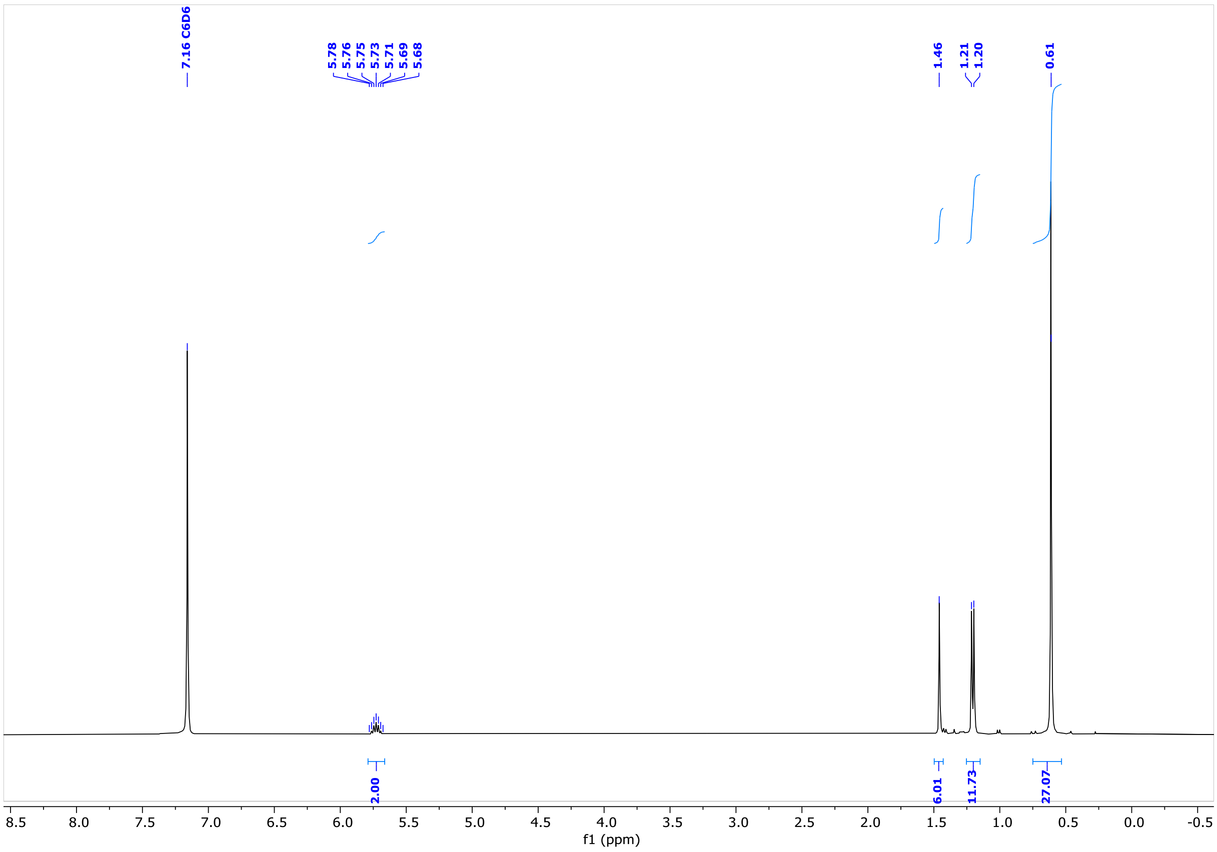

Supplementary Figure 1. ^{1}H NMR spectrum of **I^{i}Pr$_2$Al(SiTMS$_3$)I$_2$** in C_6D_6 at 300K.

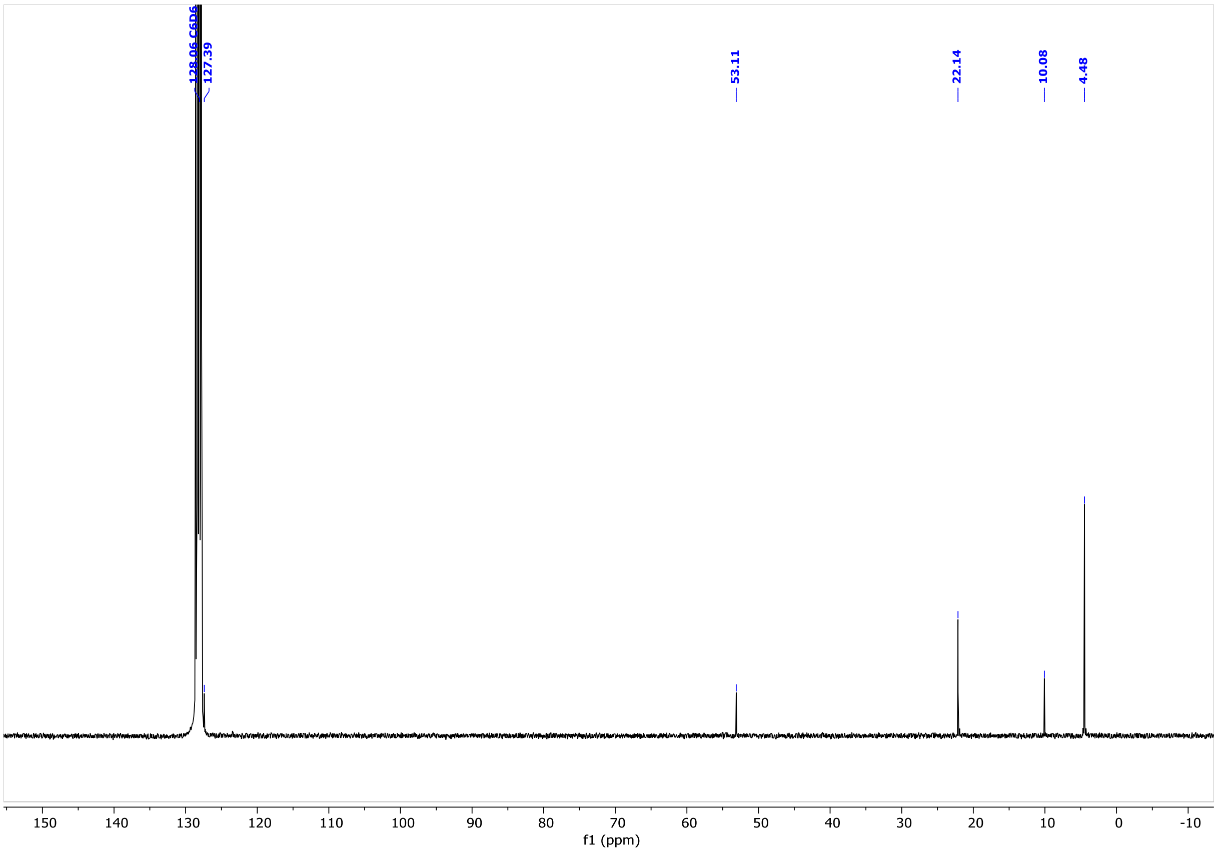

Supplementary Figure 2. ^{13}C{^{1}H} NMR spectrum of **I^{i}Pr$_2$Al(SiTMS$_3$)I$_2$** in C_6D_6 at 300K.

1.2.2 Synthesis of 1

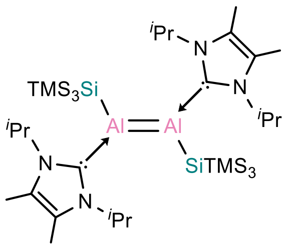

6 mL of dry benzene was added to a flask containing $I^{i}Pr_2Al(SiTMS_3)I_2$ (0.36 g, 0.508 mmol) and KC_8 (0.206 g, 1.525 mmol) at room temperature with vigorous stirring. Gradually the solution turned dark blue and the stirring was continued for 24h at RT. The deep blue solution was filtered and the solid residue was extracted with dry benzene (3 × 5 mL), until the supernatant become colorless. Afterwards, the solution was evaporated to dryness. Finally, **1** was obtained as a dark blue solid from the concentrated solution of toluene at –30 °C (0.164 g, 71% yield). X-ray quality crystals were grown from a saturated toluene solution of **1** at –30 °C over 2 days.

^{1}H NMR (400 MHz, C_6D_6): δ [ppm] 6.10 (hept, *J* = 7.1 Hz, 4H, $CH(CH_3)_2$), 1.69 (s, 12H, CH_3 NHC), 1.49 (d, *J* = 7.0 Hz, 12H, $CH(CH_3)_2$), 1.36 (d, *J* = 7.1 Hz, 12H, $CH(CH_3)_2$), 0.45 – 0.32 (m, 54H, $SiCH_3$).

^{13}C{^{1}H} NMR (101 MHz, C_6D_6): δ [ppm] 177.56 (carbene atom NHC), 126.04 (*C*=*C* NHC), 53.64 ($CH(CH_3)_2$), 22.97 ($CH(CH_3)_2$), 22.24 ($CH(CH_3)_2$), 10.06 (CH_3 NHC), 4.91 ($SiCH_3$).

^{29}Si NMR (79 MHz, C_6D_6): δ [ppm] -10.80 ($SiCH_3$). No signal was found for aluminum-bonded silicon atom, due to the quadrupolar momentum of the ^{27}Al nucleus.

Elemental Analysis (%): Calcd: C 52.80, H 10.41, N 6.16; Found: C 53.29, H 10.55, N 6.12.

UV-vis: 598 nm (Ɛ = 11351 L mol^{-1} cm^{-1}).

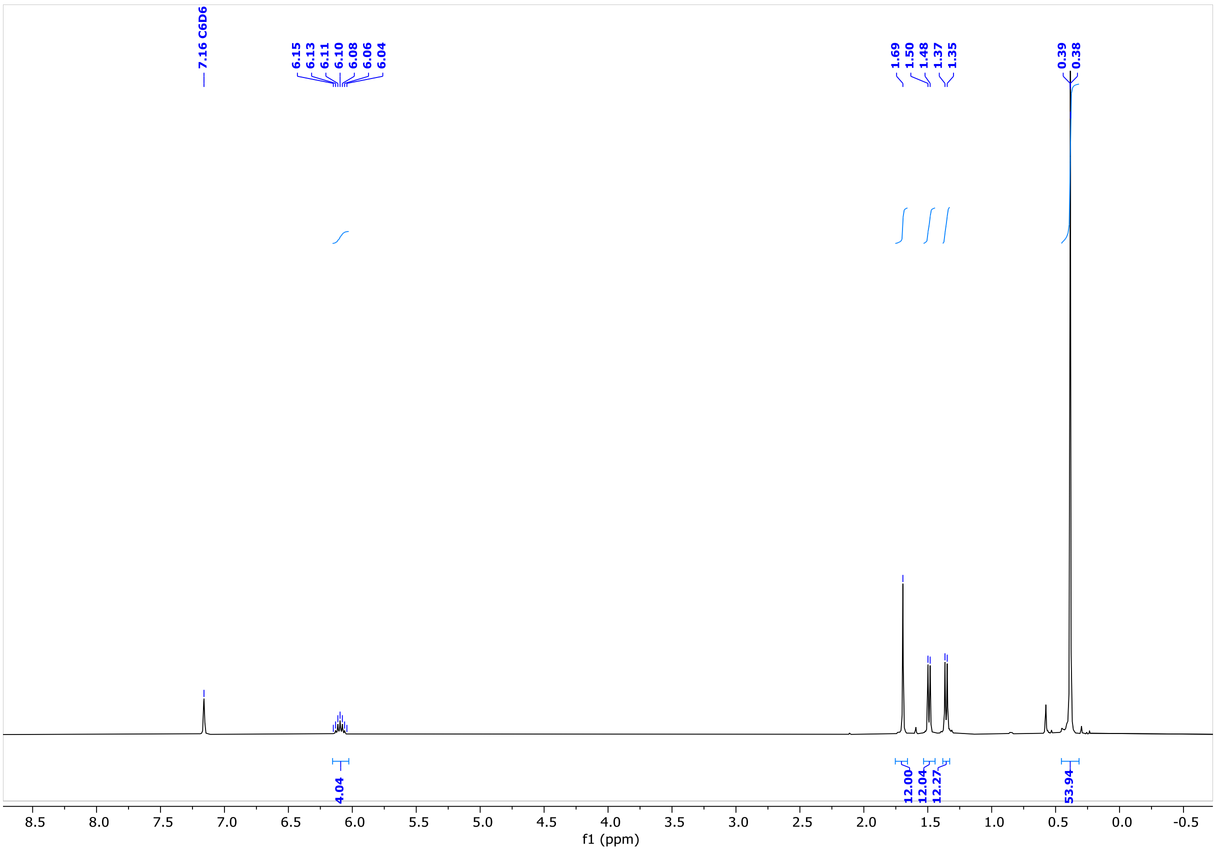

Supplementary Figure 3. ^{1}H NMR spectrum of **1** in C_6D_6 at 300K.

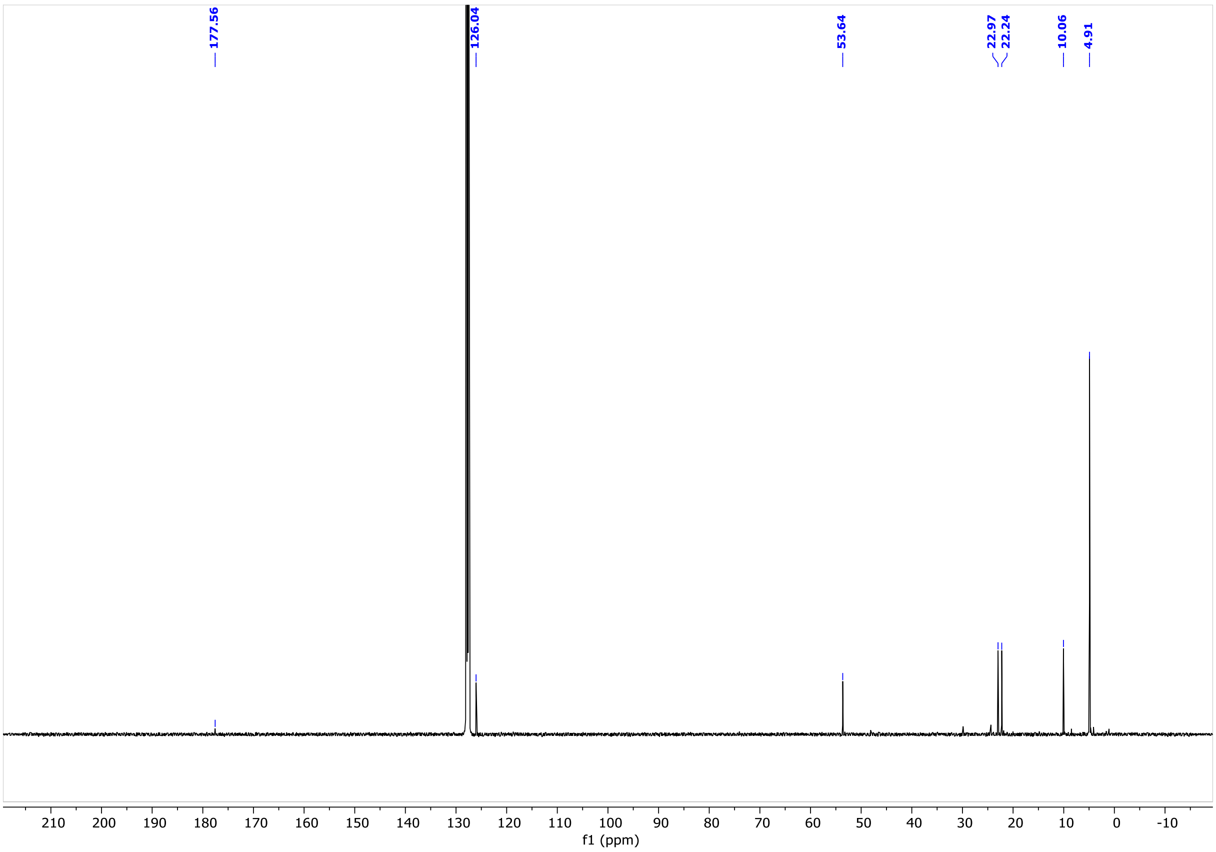

Supplementary Figure 4. $^{13}C\{^{1}H\}$ NMR spectrum of **1** in C_6D_6 at 300K.

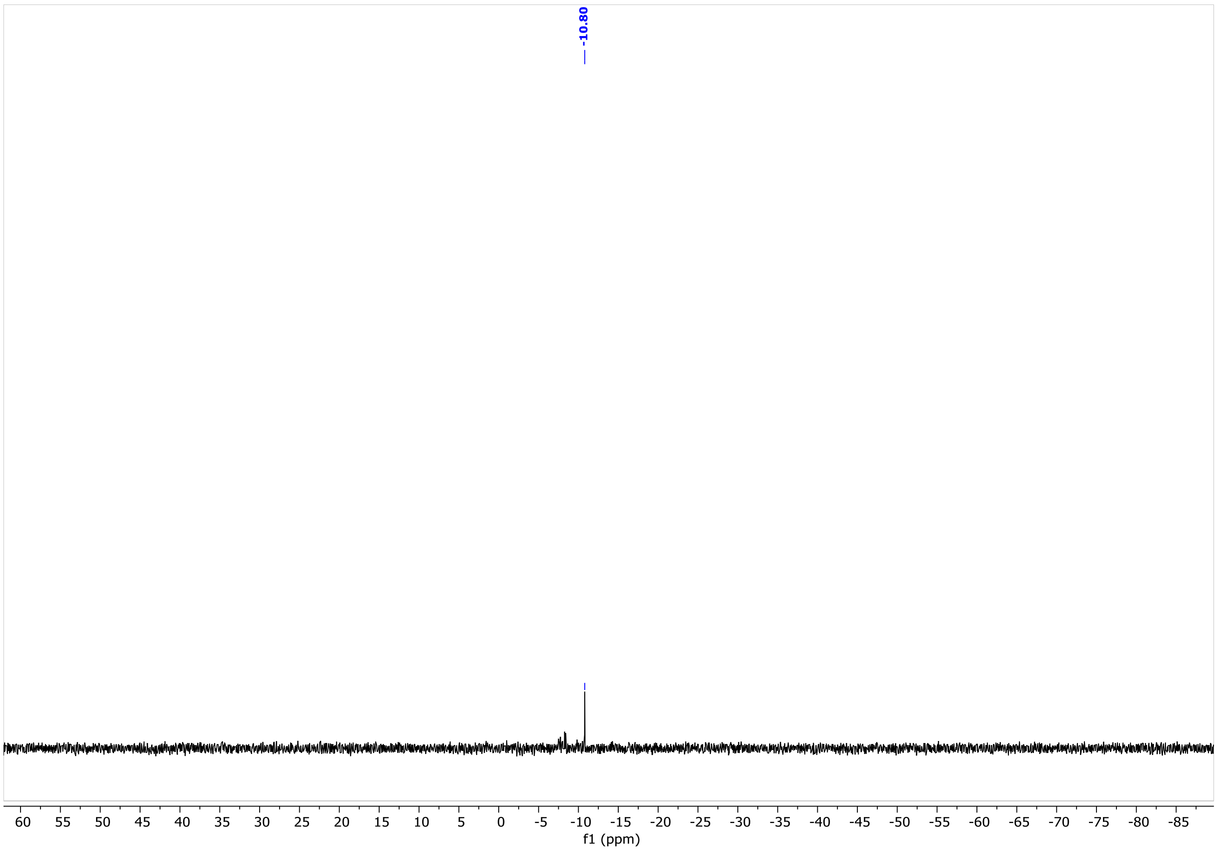

Supplementary Figure 5. ^{29}Si NMR spectrum of **1** in C_6D_6 at 300K.

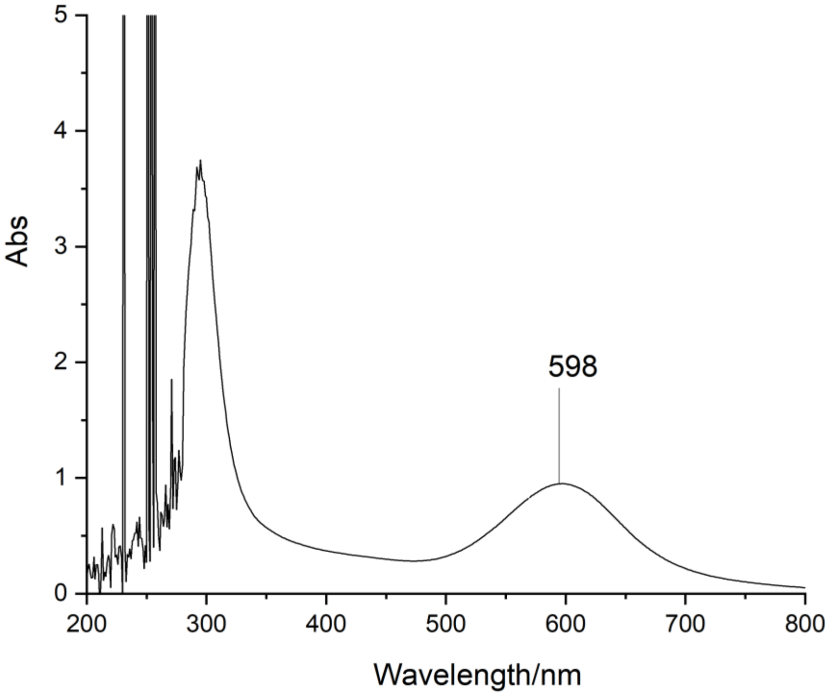

Supplementary Figure 6. UV–vis of compound **1** in toluene.

1.2.3 Synthesis of 2

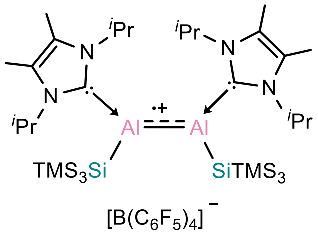

A mixture of dialumene **1** (10 mg, 0.011 mmol) and $[Ph_3C][B(C_6F_5)_4]$ (10.15 mg, 0.011 mmol) was stirred in fluorobenzene (0.3 mL) at room temperature for 10 minutes. The color of the solution changed from dark blue to dark purple. All volatiles were dried under reduced pressure, and the residue was washed with pentane (3 × 0.3 mL) to give a purple solid. The crude product was recrystallized in a mixture of fluorobenzene and pentane at -30 °C to give **2** as purple crystals (7 mg, 40% yield).

Elemental Analysis (%): Calcd: C 48.38, H 5.96, N 3.53; Found: C 48.74, H 5.76, N 3.40.

UV-vis: 550 nm (Ɛ = 3842 L mol^{-1} cm^{-1}), 957 nm (Ɛ = 3144 L mol^{-1} cm^{-1}).

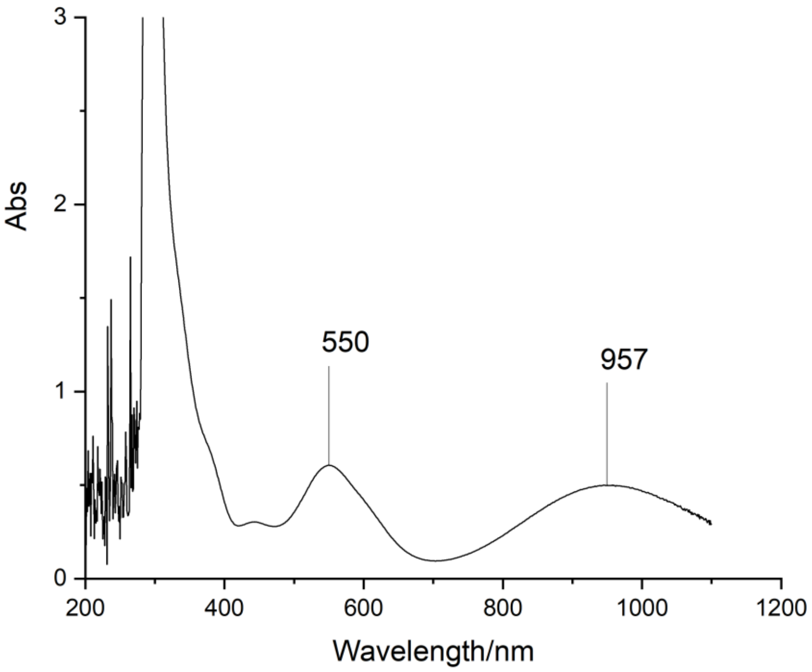

Supplementary Figure 7. UV–vis of compound **2** in a mixture of toluene and fluorobenzene.

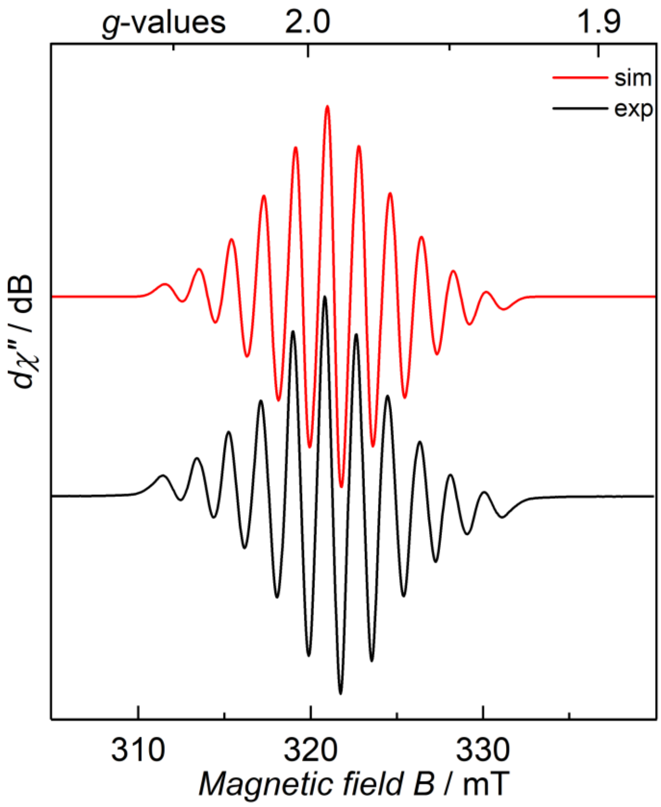

Supplementary Figure 8. CW X-band EPR spectrum of **2**, recorded in a capillary as a 3 mM *o*-DFB solution at 293 K (black trace), and its simulation (red trace). Experimental conditions: microwave frequency v = 8.987 GHz, modulation amplitude = 1.0 mT, microwave power = 1.00 mW, modulation frequency = 100 kHz, time constant = 0.1 s. Simulation parameters: g_{iso} = 1.9981, linewidths W_{iso} = 0.853 mT. Hyperfine coupling to *two* ^{27}Al nuclei (I = 5/2, 100% nat. abundance) was determined as A_{iso} = 17.1 · 10^{-4} cm^{-1} (1.83 mT). *A*-strain effects were considered by using m_I square-dependent linewidth broadening $c2 \cdot m_I^2$ with parameter c2 = 0.141 · $10^{-4}cm^{-1}$.

1.2.4 Synthesis of 3

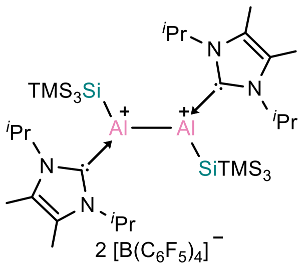

A mixture of dialumene **1** (10 mg, 0.011 mmol) and $[Ph_3C][B(C_6F_5)_4]$ (20.3 mg, 0.022 mmol) was stirred in fluorobenzene (0.4 mL) at room temperature for 10 minutes. The color of the solution changed from dark blue to light yellow, along with the precipitation of a pale yellow solid. 0.5 mL of cold pentane was added to the reaction mixture, and the resulting precipitate was washed with pentane (2 × 0.4 mL) to afford **3** as a pale yellow solid (20.6 mg, 83% yield). X-ray quality crystals were grown from a mixture of 1,2-difluorobenzene and pentane at -30 °C over 2 days.

^{1}H NMR (400 MHz, DFB+C_6D_6): δ [ppm] 4.58 – 4.37 (m, 4H, C*H*$(CH_3)_2$), 2.22 (s, 12H, *CH$_3$* NHC), 1.91 (d, *J* = 6.4 Hz, 12H, CH(C*H*$_3$)$_2$), 1.59 (d, *J* = 6.4 Hz, 12H, CH(C*H*$_3$)$_2$), 0.12 (s, 54H, SiC*H*$_3$).

^{11}B NMR (128 MHz, DFB+C_6D_6): δ [ppm] -16.24.

^{19}F NMR (377 MHz, DFB+C_6D_6): δ [ppm] -132.43 (br), -163.77 (t, *J* = 18.9 Hz), -167.50 (br).

It was not possible to measure ^{13}C{^{1}H} NMR and ^{29}Si NMR, due to the poor solubility of **3** in DFB.

Elemental Analysis (%): Calcd: C 46.60, H 4.18, N 2.47; Found: C 48.57, H 3.90, N 2.35.

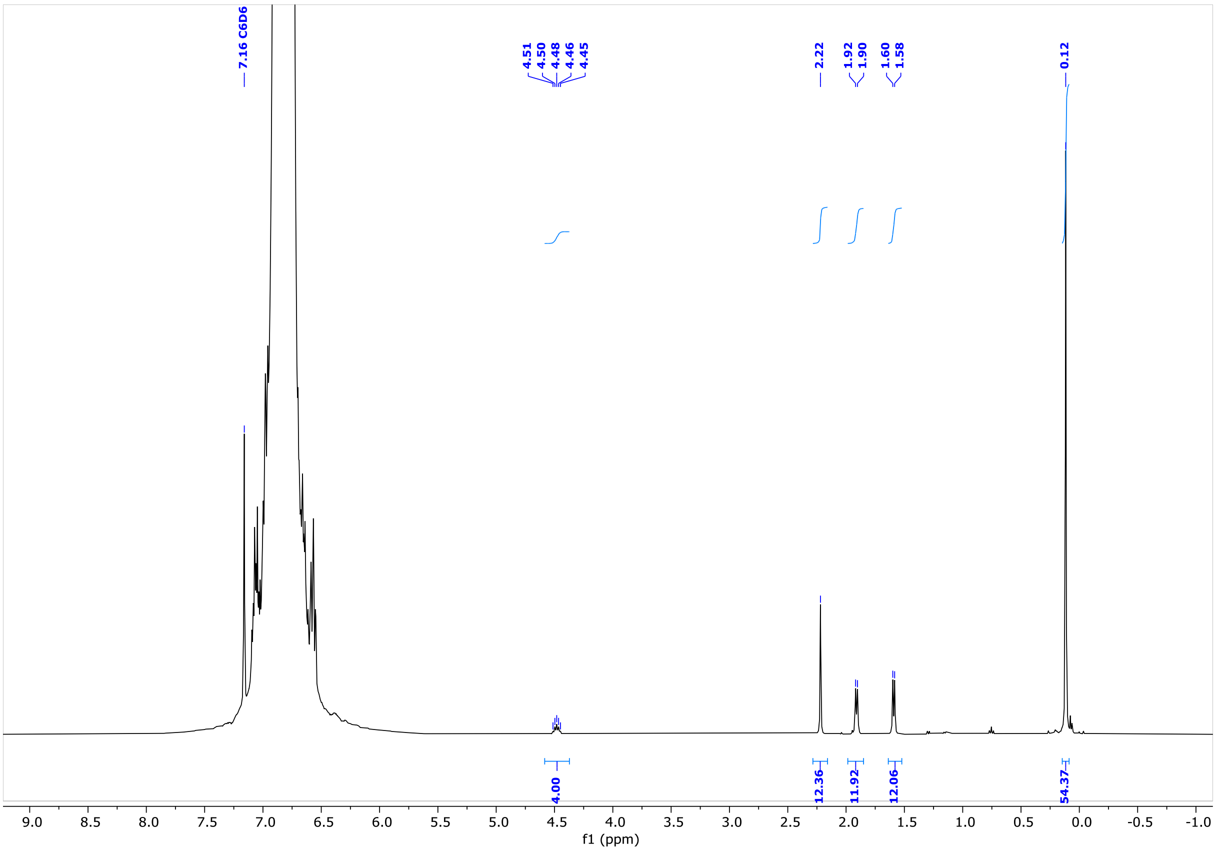

Supplementary Figure 9. ^{1}H NMR spectrum of **3** in a mixture of DFB and C_6D_6 at 300K.

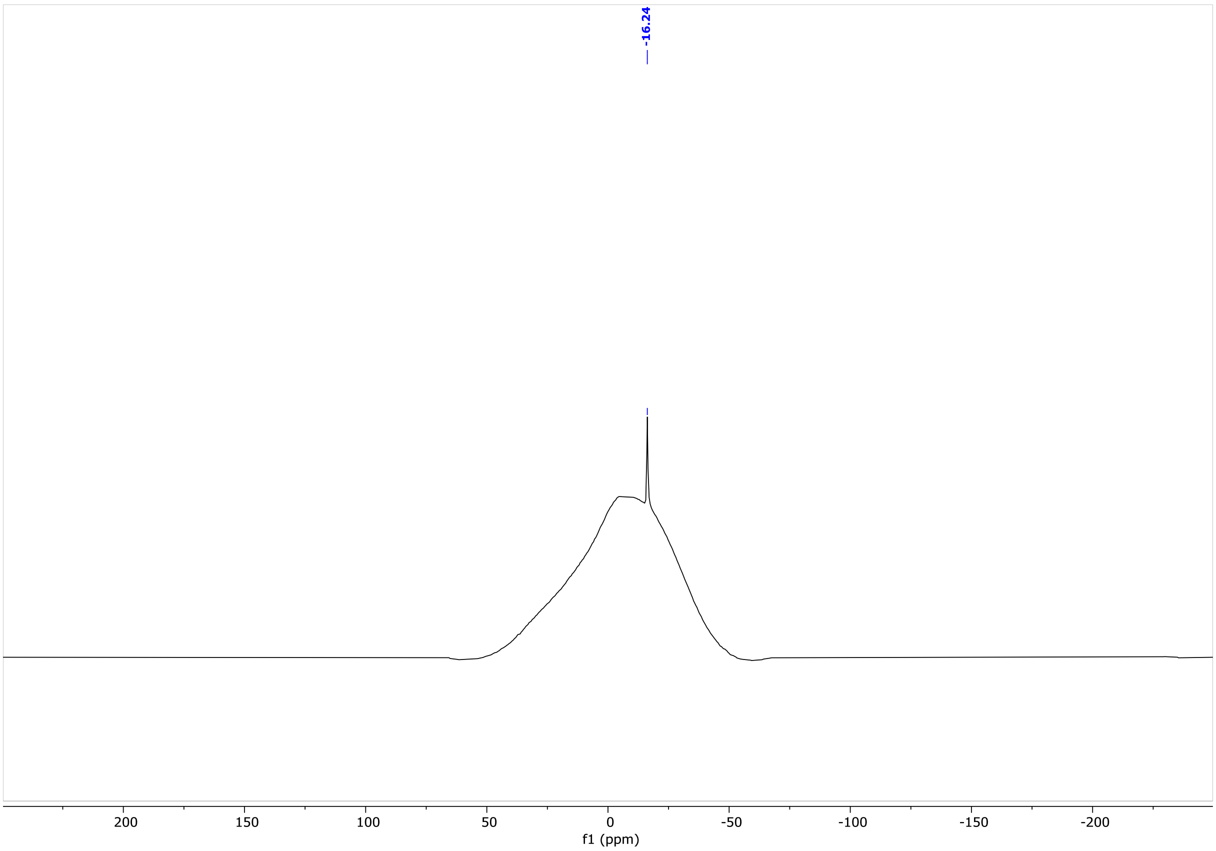

Supplementary Figure 10. ^{11}B NMR spectrum of **3** in a mixture of DFB and C_6D_6 at 300K.

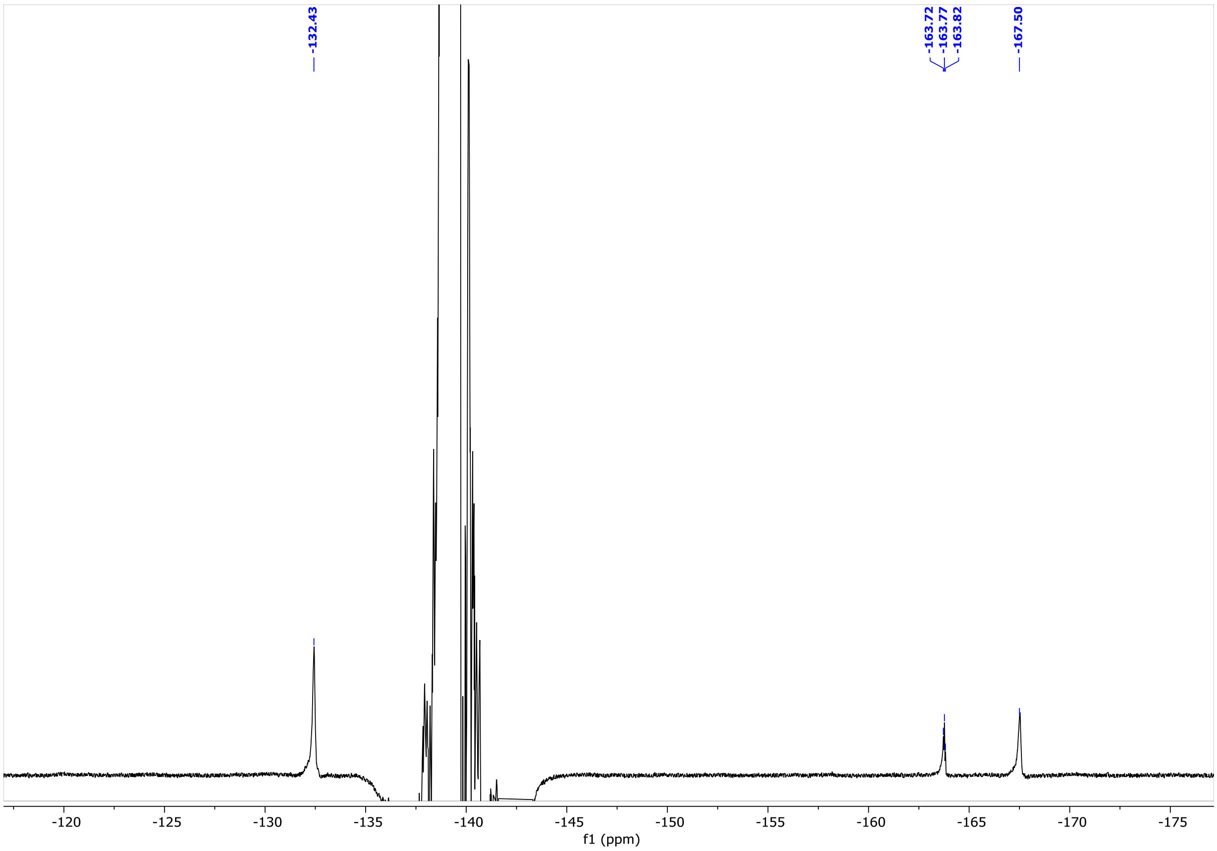

Supplementary Figure 11. ^{19}F NMR spectrum of **3** in a mixture of DFB and C_6D_6 at 300K.

1.2.5 Synthesis of 4a

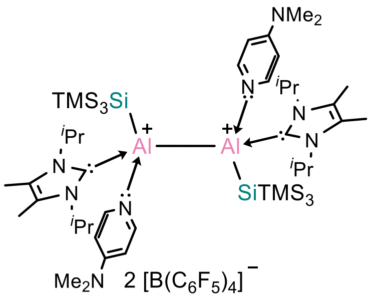

A mixture of **3** (25 mg, 0.011 mmol) and DMAP (2.7 mg, 0.022 mmol) was stirred in 1,2-difluorobenzene (0.4 mL) at room temperature for 10 minutes. The color of the solution changed from pale yellow to colorless. The crude product was recrystallized in a mixture of 1,2-difluorobenzene and pentane at -30 °C to give **4a** as colorless crystals (20 mg, 72% yield).

^{1}H NMR (400 MHz, DFB+C_6D_6): δ [ppm] 8.79 (s, 2H, C*H* in DMAP), 8.59 (s, 2H, C*H* in DMAP), 7.20 – 6.40 (m, 4H, C*H* in DMAP, overlap with the DFB solvent), 4.23 – 4.06 (m, 2H, C*H*(CH_3)$_2$),

3.97 – 3.77 (m, 2H, C*H*$(CH_3)_2$), 3.09 (s, 12H, N(CH_3)$_2$), 2.01 (d, *J* = 2.0 Hz, 12H, *CH₃* NHC), 1.35 (d, *J* = 6.9 Hz, 6H, CH(CH_3)$_2$), 1.10 (d, *J* = 6.9 Hz, 6H, CH(CH_3)$_2$), 1.05 (d, *J* = 6.8 Hz, 6H, CH(CH_3)$_2$), 0.65 (d, *J* = 6.9 Hz, 6H, CH(CH_3)$_2$), 0.16 (s, 54H, SiCH_3).

^{11}B NMR (128 MHz, DFB+C_6D_6): δ [ppm] -16.27.

^{19}F NMR (377 MHz, DFB+C_6D_6): δ [ppm] -132.47 (br), -163.83 (t, *J* = 20.2 Hz), -167.58 (t, *J* = 19.5 Hz).

It was not possible to measure ^{13}C{^{1}H} NMR and ^{29}Si NMR, due to the poor solubility of **4a** in DFB.

Elemental Analysis (%): Calcd: C 48.77, H 4.57, N 4.46; Found: C 49.76, H 4.37, N 4.30.

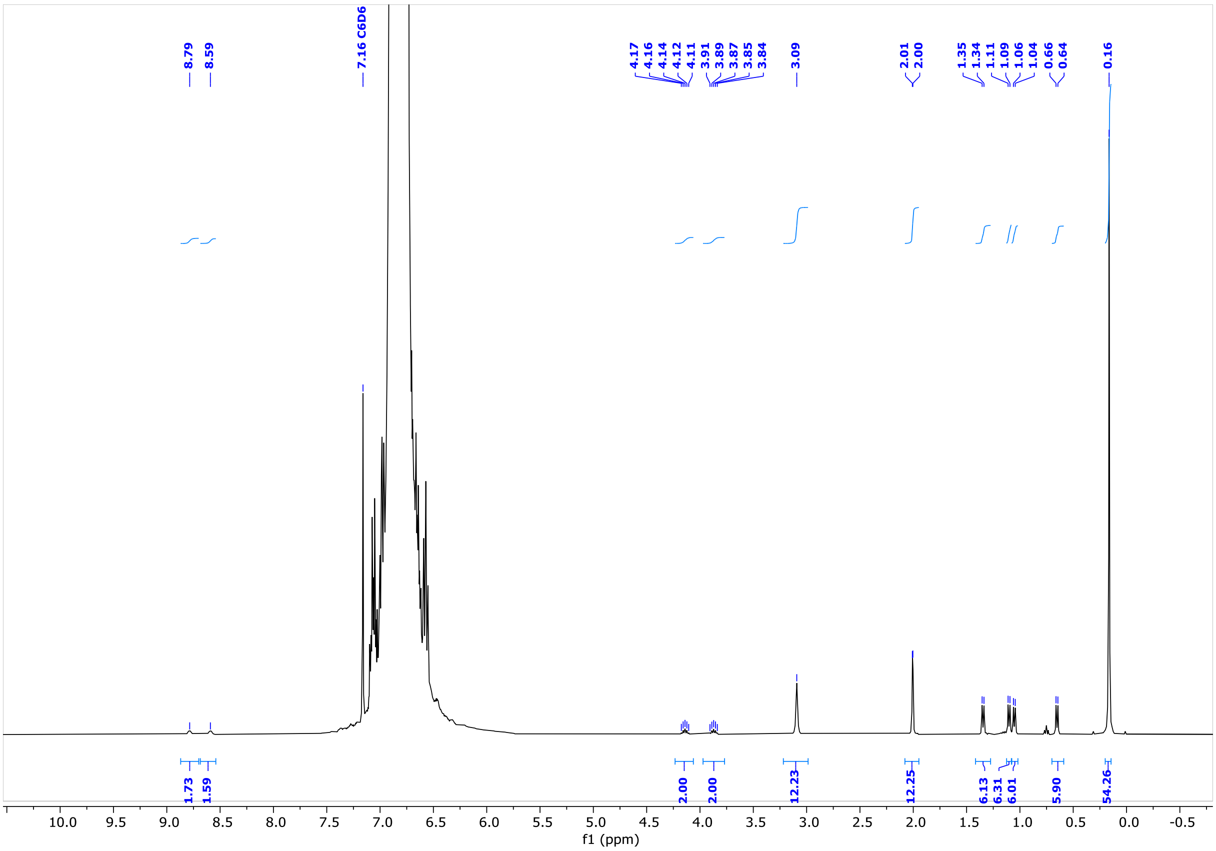

Supplementary Figure 12. ^{1}H NMR spectrum of **4a** in a mixture of DFB and C_6D_6 at 300K.

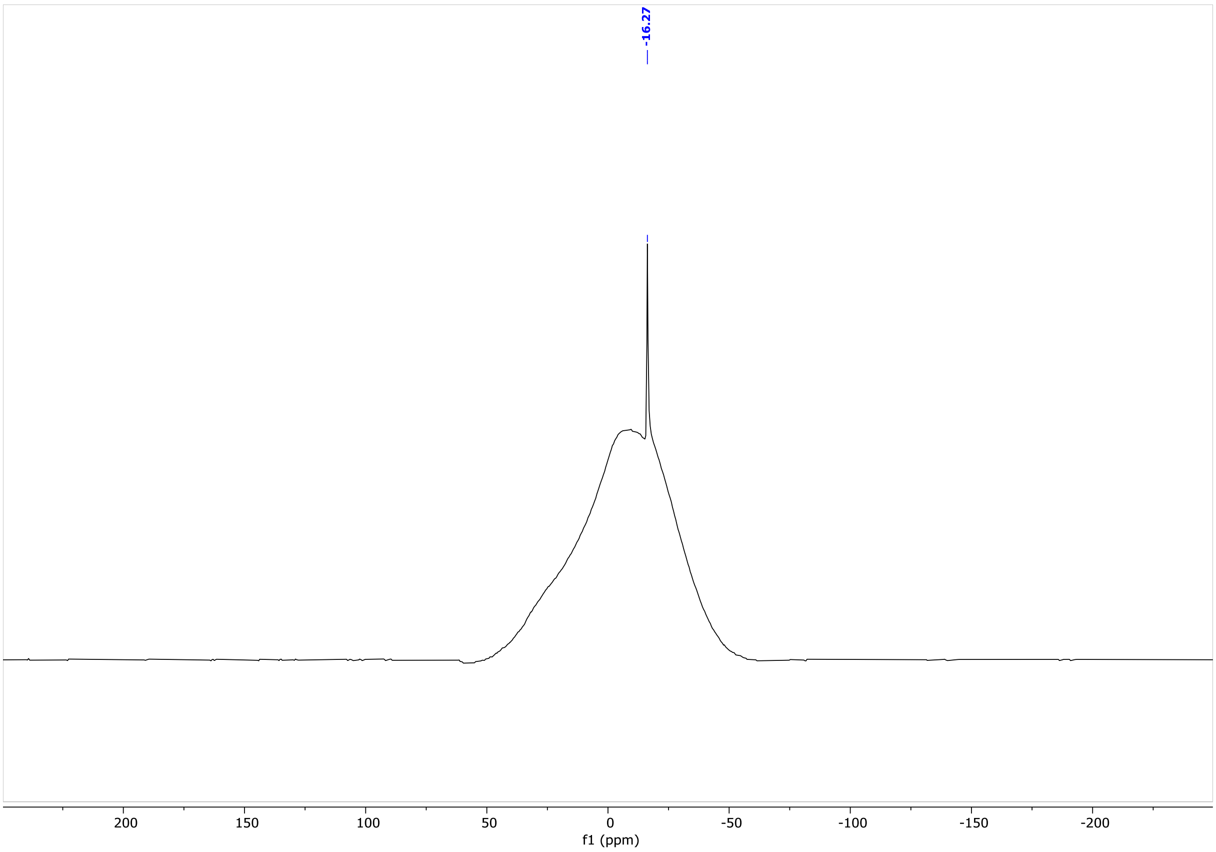

Supplementary Figure 13. ^{11}B NMR spectrum of **4a** in a mixture of DFB and C_6D_6 at 300K.

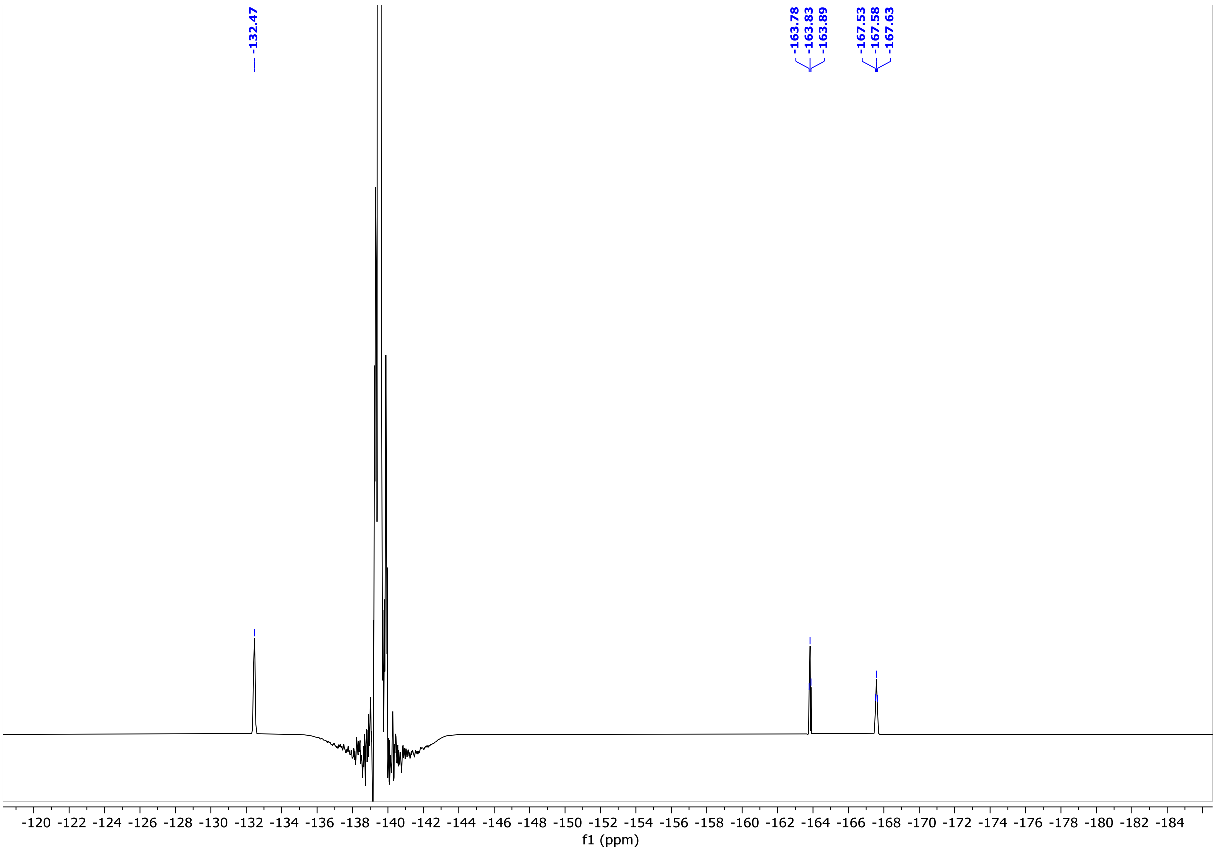

Supplementary Figure 14. ^{19}F NMR spectrum of **4a** in a mixture of DFB and C_6D_6 at 300K.

1.2.6 Synthesis of 4b

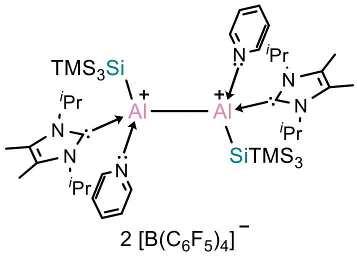

Treatment of **3** (25 mg, 0.011 mmol) with pyridine (1.74 mg, 0.022 mmol) in 1,2-difluorobenzene (0.6 mL) at room temperature resulted in an instant color change from pale yellow to light yellow. Light yellow crystals (suitable for XRD) were observed in the reaction solution which were isolated via filtration (18 mg, 67%).

It was not possible to measure NMR, due to the poor solubility of **4b** in DFB.

Elemental Analysis (%): Calcd: C 48.52, H 4.32, N 3.46; Found: C 49.69, H 4.35, N 3.13.

1.2.7 Synthesis of 5

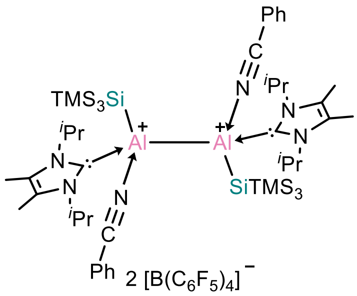

A mixture of **3** (11 mg, 0.0049 mmol) and PhCN (1 mg, 0.0097 mmol) was stirred in 1,2-difluorobenzene (0.3 mL) at room temperature for 10 minutes. The color of the solution changed from pale yellow to bright yellow. The crude product was recrystallized in a mixture of 1,2-difluorobenzene and pentane at -30 °C to give **5** as yellow crystals (10.1 mg, 84% yield).

^{1}H NMR (400 MHz, DFB+C_6D_6): δ [ppm] 7.93 (s, 4H, C*H* in Ph), 7.67 (s, 2H, C*H* in Ph), 7.51 (s, 4H, C*H* in Ph), 5.26 – 4.08 (broad, 4H, C*H*(CH_3)$_2$), 2.16 (s, 12H, *CH₃* NHC), 1.51 (broad, 24H, CH(C*H*$_3$)$_2$), 0.16 (s, 54H, SiC*H*$_3$).

^{11}B NMR (128 MHz, DFB+C_6D_6): δ [ppm] -16.25.

^{19}F NMR (377 MHz, DFB+C_6D_6): δ [ppm] -132.40 (br), -163.71 (t, J = 20.3 Hz), -167.49 (t, J = 19.4 Hz).

It was not possible to measure $^{13}C\{^{1}H\}$ NMR and ^{29}Si NMR, due to the poor solubility of **5** in DFB.

Elemental Analysis (%): Calcd: C 49.52, H 4.24, N 3.40; Found: C 50.77, H 3.87, N 3.24.

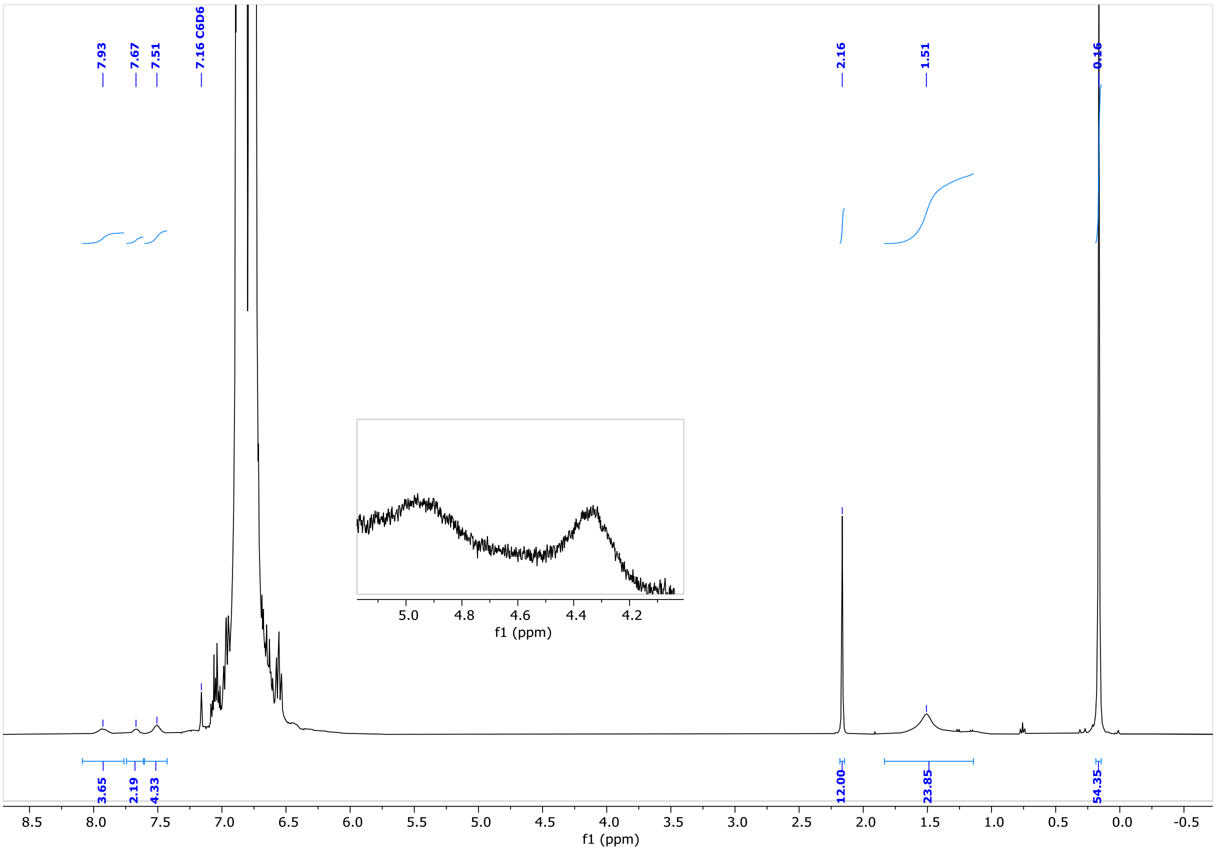

Supplementary Figure 15. ^{1}H NMR spectrum of **5** in a mixture of DFB and C_6D_6 at 300K.

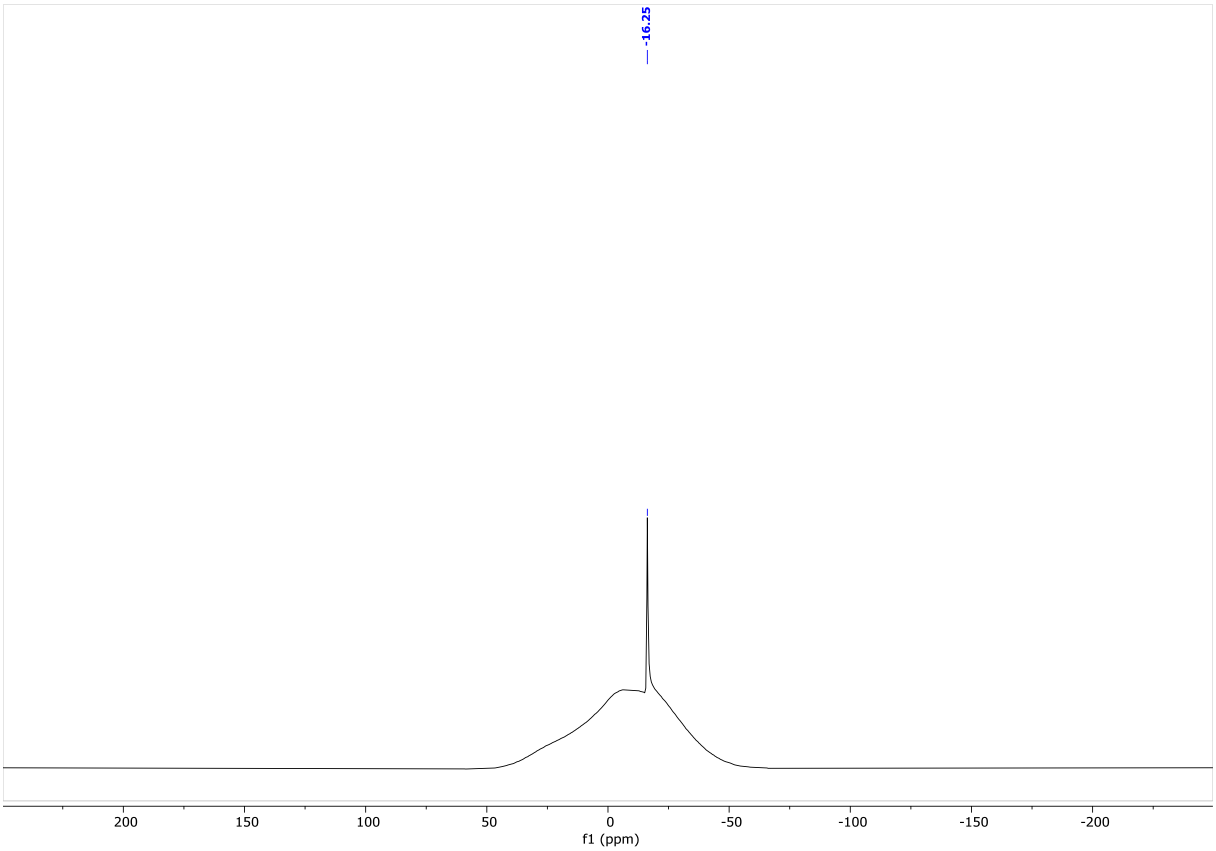

Supplementary Figure 16. ^{11}B NMR spectrum of **5** in a mixture of DFB and C_6D_6 at 300K.

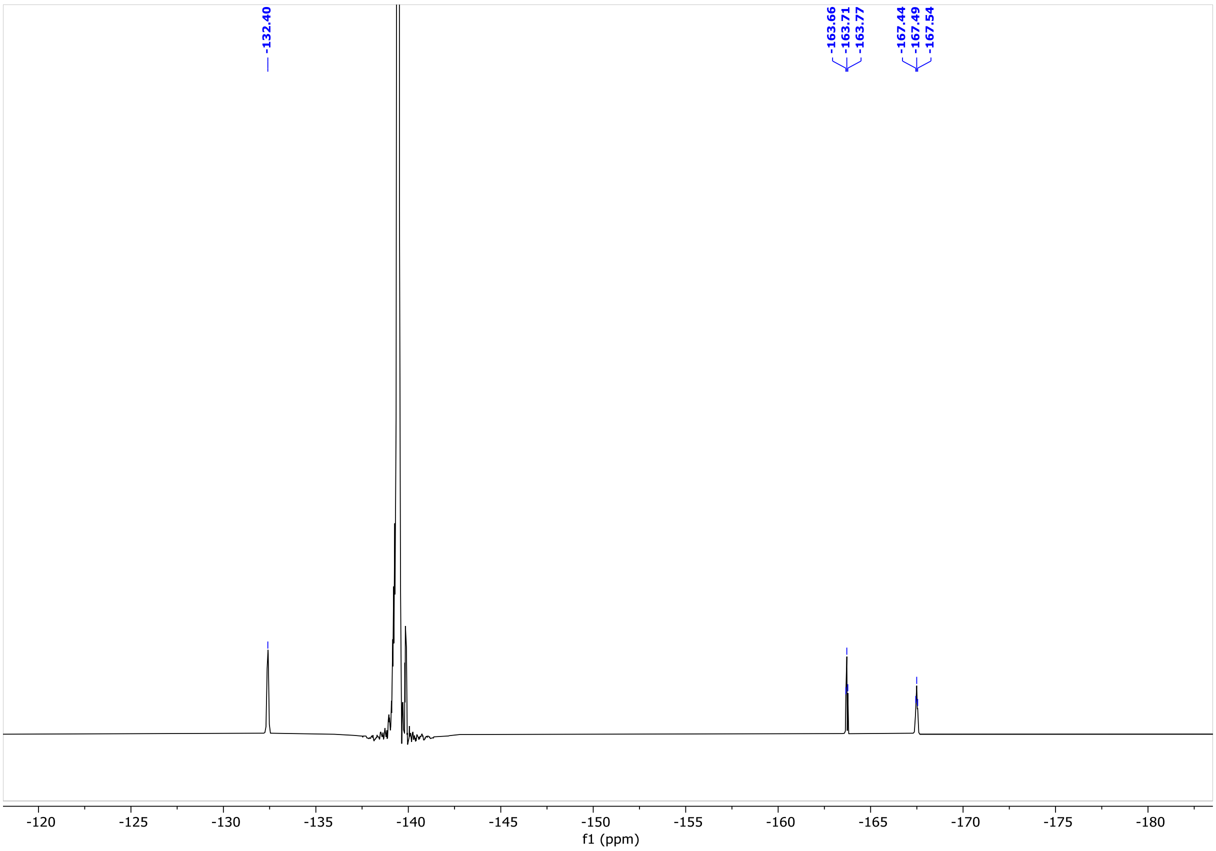

Supplementary Figure 17. ^{19}F NMR spectrum of **5** in a mixture of DFB and C_6D_6 at 300K.

1.2.8 Synthesis of 6

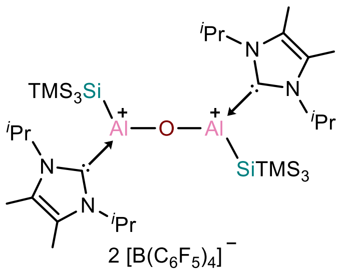

Exposure a 1,2-difluorobenzene solution of **3** (20 mg, 0.0088 mmol) to N_2O (2 bar) resulted in a color change from pale yellow to colorless after stirring at room temperature for 1 day. The crude product was recrystallized in a mixture of 1,2-difluorobenzene and pentane at -30 °C to give **6** as colorless crystals (8.6 mg, 43% yield).

^{1}H NMR (400 MHz, DFB+C_6D_6): δ [ppm] 4.53 – 4.37 (m, 4H, C*H*(CH_3)$_2$), 2.13 (s, 12H, *CH$_3$* NHC), 2.07 (d, *J* = 6.5 Hz, 12H, CH(C*H$_3$*)$_2$), 1.52 (d, *J* = 6.3 Hz, 12H, CH(C*H$_3$*)$_2$), 0.10 (s, 54H, SiC*H$_3$*).

^{13}C{^{1}H} NMR (101 MHz, DFB+C_6D_6): δ [ppm] 129.97 (*C*=*C* NHC), 50.53 (*C*H(CH_3)$_2$), 26.20 (CH(*C*H$_3$)$_2$), 23.86 (CH(*C*H$_3$)$_2$), 7.76 (*C*H$_3$ NHC), 2.43 (Si*C*H$_3$).

^{29}Si NMR (79 MHz, DFB+C_6D_6): δ [ppm] -8.27 (*Si*CH$_3$).

No signal was found for aluminum-bonded silicon and carbene carbon atom, due to the quadrupolar momentum of the ^{27}Al nucleus.

^{11}B NMR (128 MHz, DFB+C_6D_6): δ [ppm] -16.25.

^{19}F NMR (377 MHz, DFB+C_6D_6): δ [ppm] -132.42 (br), -163.76 (t, *J* = 20.2 Hz), -167.56 (d, *J* = 19.2 Hz).

Elemental Analysis (%): Calcd: C 46.28, H 4.15, N 2.45; Found: C 46.42, H 4.13, N 2.42.

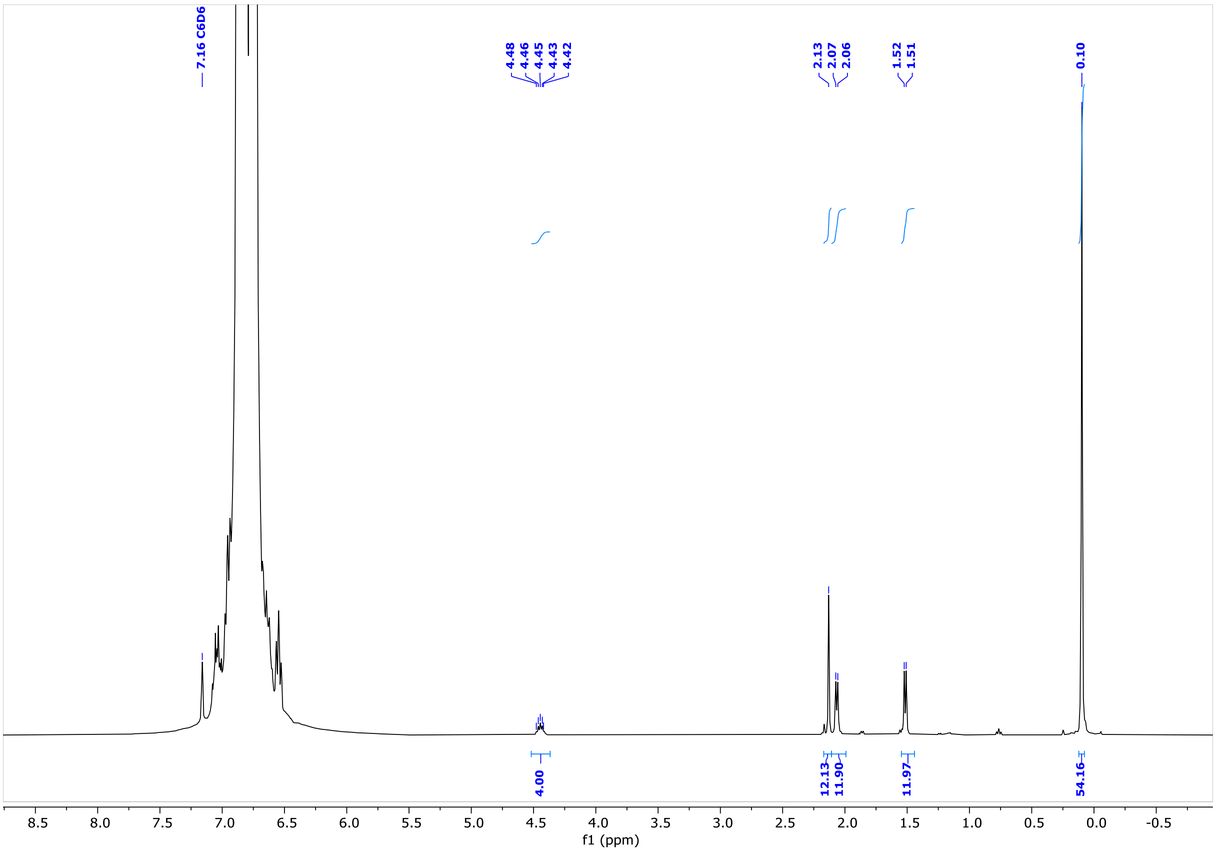

Supplementary Figure 18. ^{1}H NMR spectrum of **6** in a mixture of DFB and C_6D_6 at 300K.

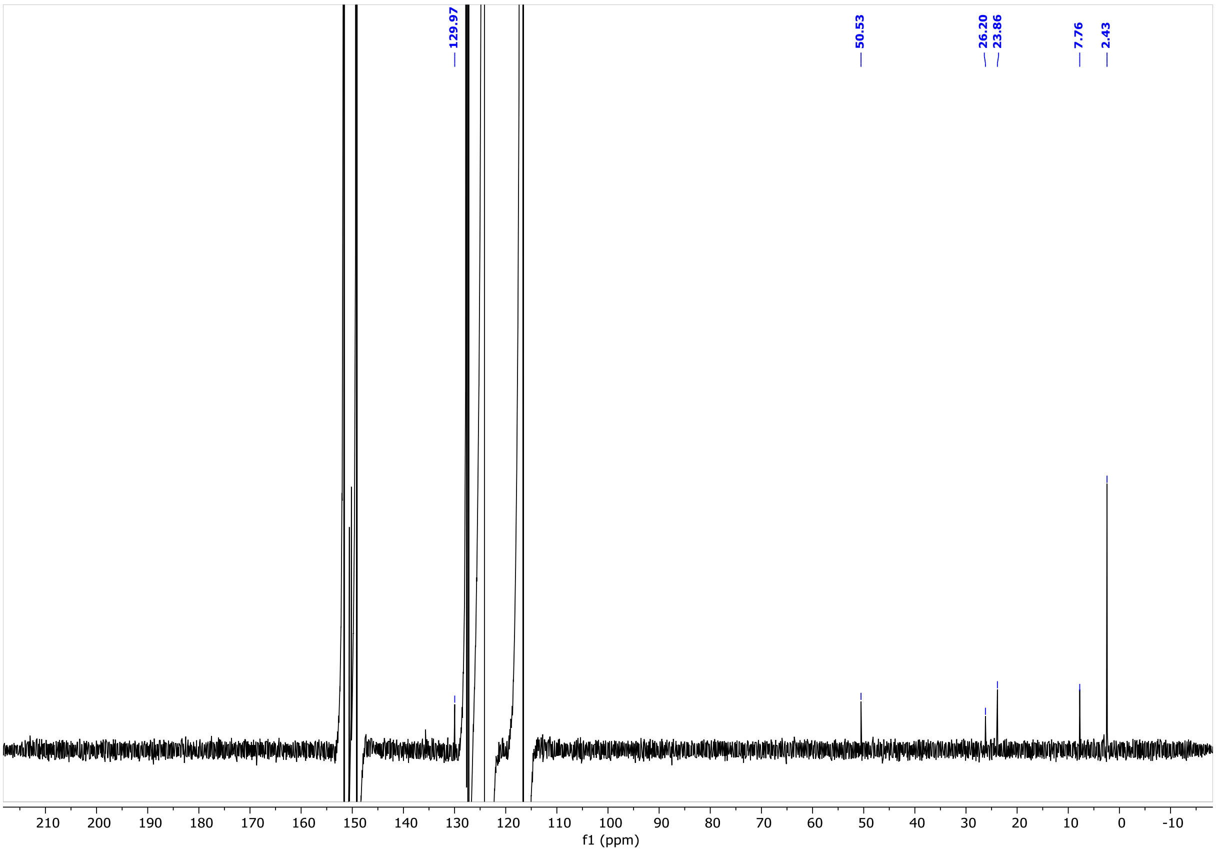

Supplementary Figure 19. $^{13}C\{^{1}H\}$ NMR spectrum of **6** in a mixture of DFB and C_6D_6 at 300K.

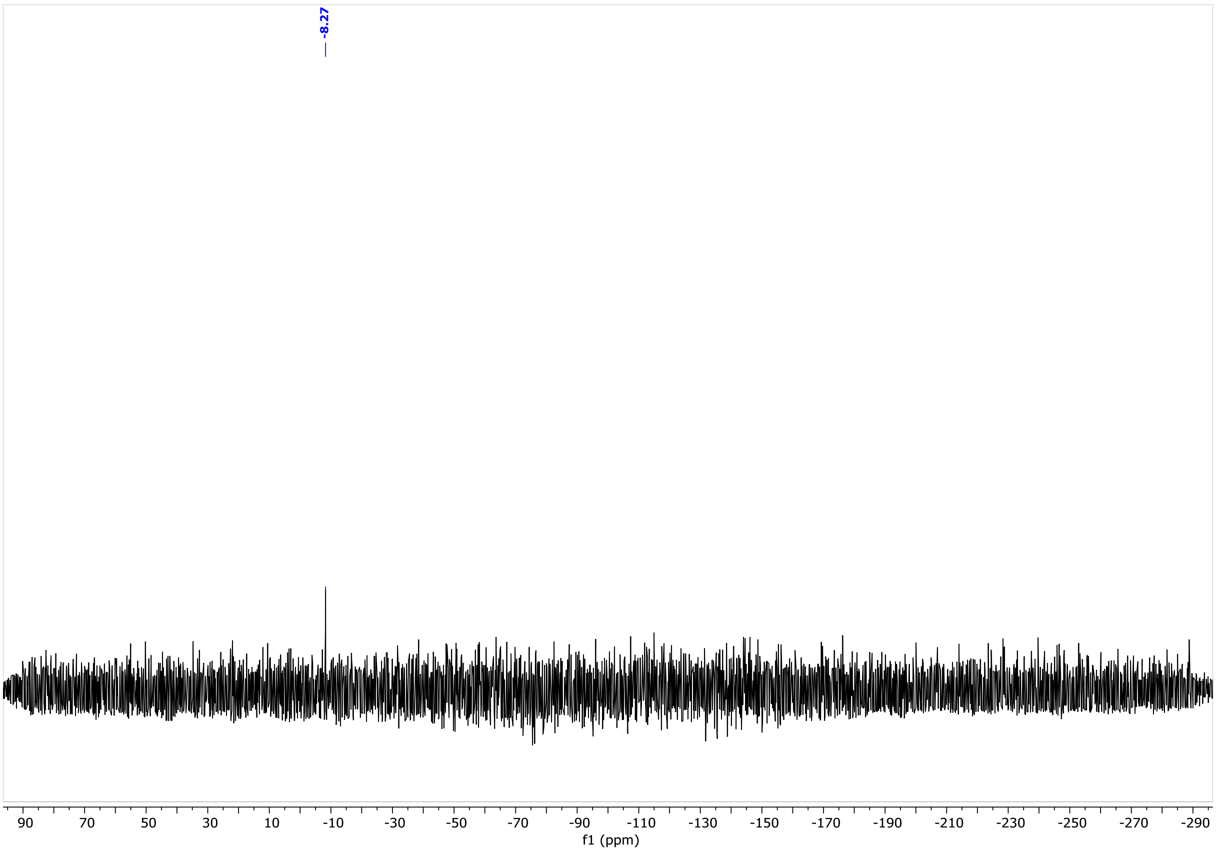

Supplementary Figure 20. ^{29}Si NMR spectrum of **6** in a mixture of DFB and C_6D_6 at 300K.

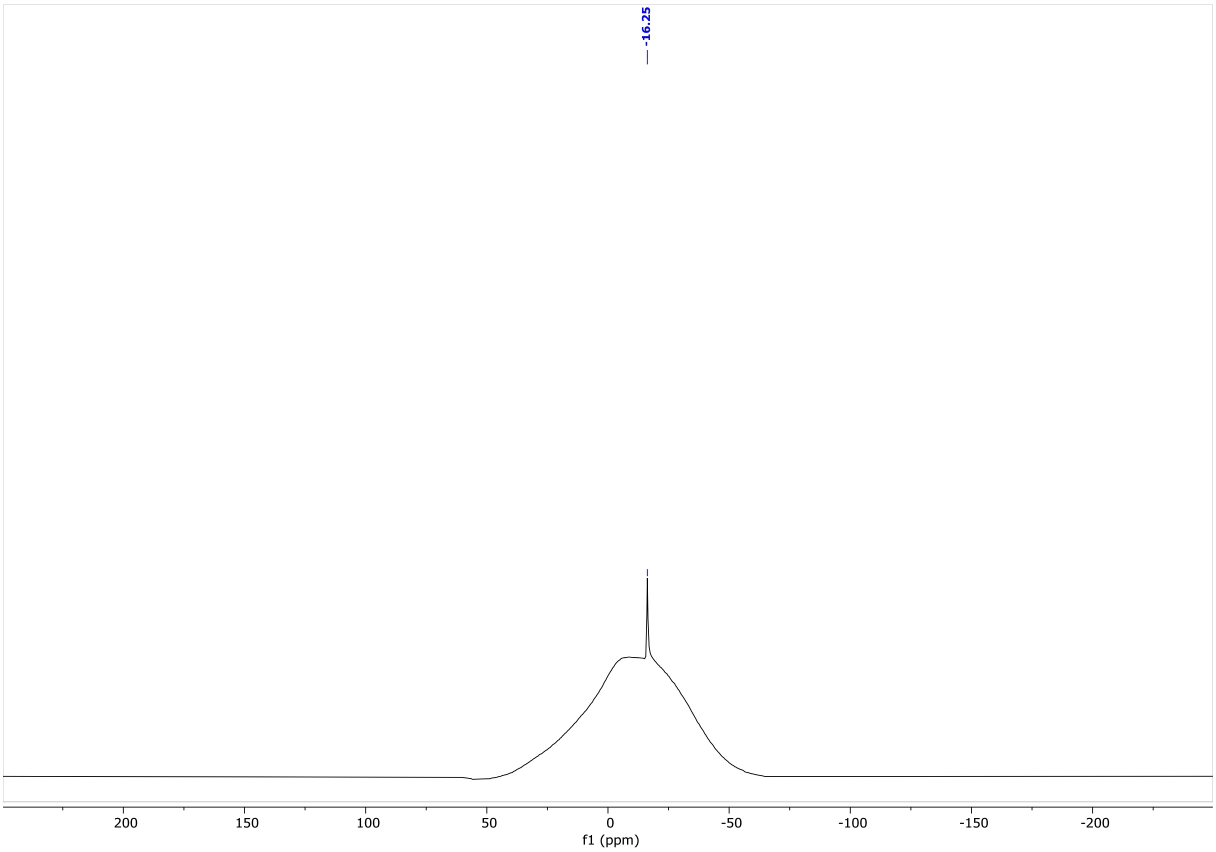

Supplementary Figure 21. ^{11}B NMR spectrum of **6** in a mixture of DFB and C_6D_6 at 300K.

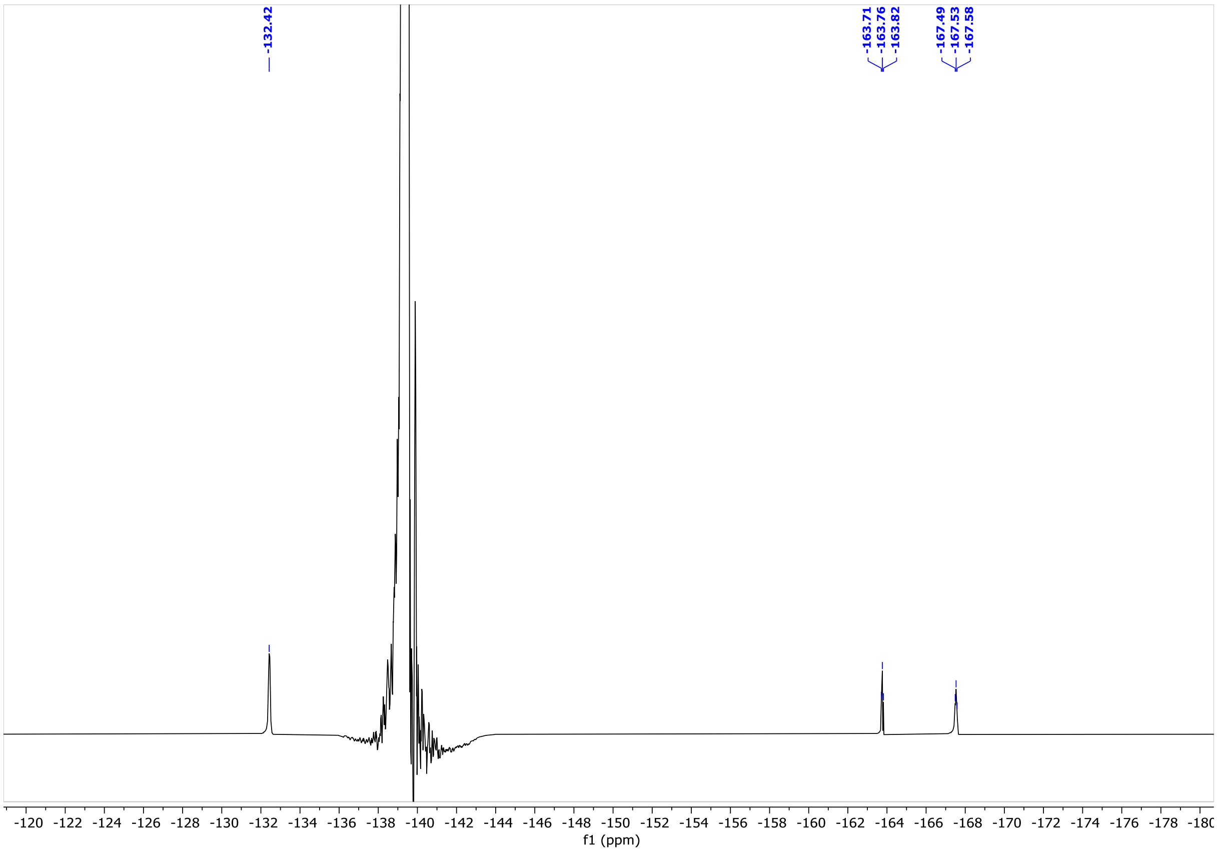

Supplementary Figure 22. ^{19}F NMR spectrum of **6** in a mixture of DFB and C_6D_6 at 300K.

1.2.9 Synthesis of 7

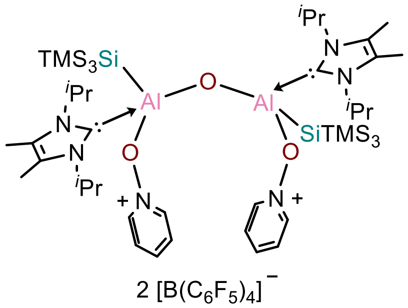

A mixture of **3** (12 mg, 0.0053 mmol) and pyridine-N-oxide (1.5 mg, 0.0159 mmol) was stirred in 1,2-difluorobenzene (0.4 mL) at room temperature. The color of the solution immediately changed from pale yellow to colorless. Recrystallization of the crude product from a 1,2-difluorobenzene/pentane mixture at –30 °C yielded compound **7** as a few colorless crystals. However, compound **7** is highly unstable in solution, preventing the collection of clean NMR data.

1.2.10 Synthesis of 8

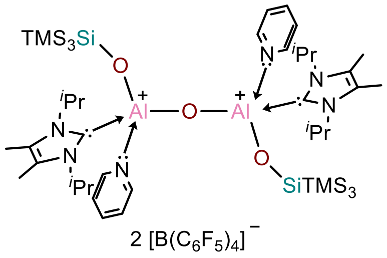

The dication **3** (12 mg, 0.0053 mmol) and pyridine-N-oxide (1.5 mg, 0.0159 mmol) was mixed in 1,2-difluorobenzene (0.4 mL) at room temperature, which resulted in an instant color change from pale yellow to colorless. The reaction mixture was then recrystallized from a 1,2-difluorobenzene/pentane mixture at –30 °C. The resulting solid was redissolved in 1,2-difluorobenzene and stirred at room temperature for 24 h, leading to complete consumption of intermediate **7**. Colorless crystals of **8** were obtained from a mixture of 1,2-difluorobenzene and pentane at -30 °C (6.3 mg, 48% yield).

^{1}H NMR (400 MHz, DFB+C_6D_6): δ [ppm] 8.78 (d, *J* = 5.5 Hz, 4H, C*H* in pyridine), 8.25 (t, *J* = 7.8 Hz, 2H, C*H* in pyridine), 7.87 (t, *J* = 6.7 Hz, 4H, C*H* in pyridine), 4.52 – 4.33 (m, 4H, C*H*(CH_3)$_2$), 2.01 (s, 12H, *CH_3* NHC), 1.09 (d, *J* = 7.0 Hz, 24H, CH(C*H_3*)$_2$), 0.06 (s, 54H, SiC*H_3*).

^{13}C{^{1}H} NMR (101 MHz, DFB+C_6D_6): δ [ppm] 147.63 (carbon in pyridine ring, other aromatic carbon peaks are overlapped with DFB solvent), 129.37 (*C*=*C* NHC), 53.17 (*C*H(CH_3)$_2$), 21.35 (CH(*C*H_3)$_2$), 21.00 (CH(*C*H_3)$_2$), 9.28 (*C*H_3 NHC), -0.15 (Si*C*H_3). No signal was found for aluminum-bonded carbene carbon atom, due to the quadrupolar momentum of the ^{27}Al nucleus.

^{29}Si NMR (79 MHz, DFB+C_6D_6): δ [ppm] -8.49 (*Si*CH_3), -17.50 (*Si*TMS_3).

^{11}B NMR (128 MHz, DFB+C_6D_6): δ [ppm] -16.26.

^{19}F NMR (377 MHz, DFB+C_6D_6): δ [ppm] -132.31 – -132.58 (m), -163.75 (t, *J* = 20.3 Hz), -167.53 (t, *J* = 19.4 Hz).

Elemental Analysis (%): Calcd: C 47.58, H 4.24, N 3.40; Found: C 48.01, H 4.18, N 3.27.

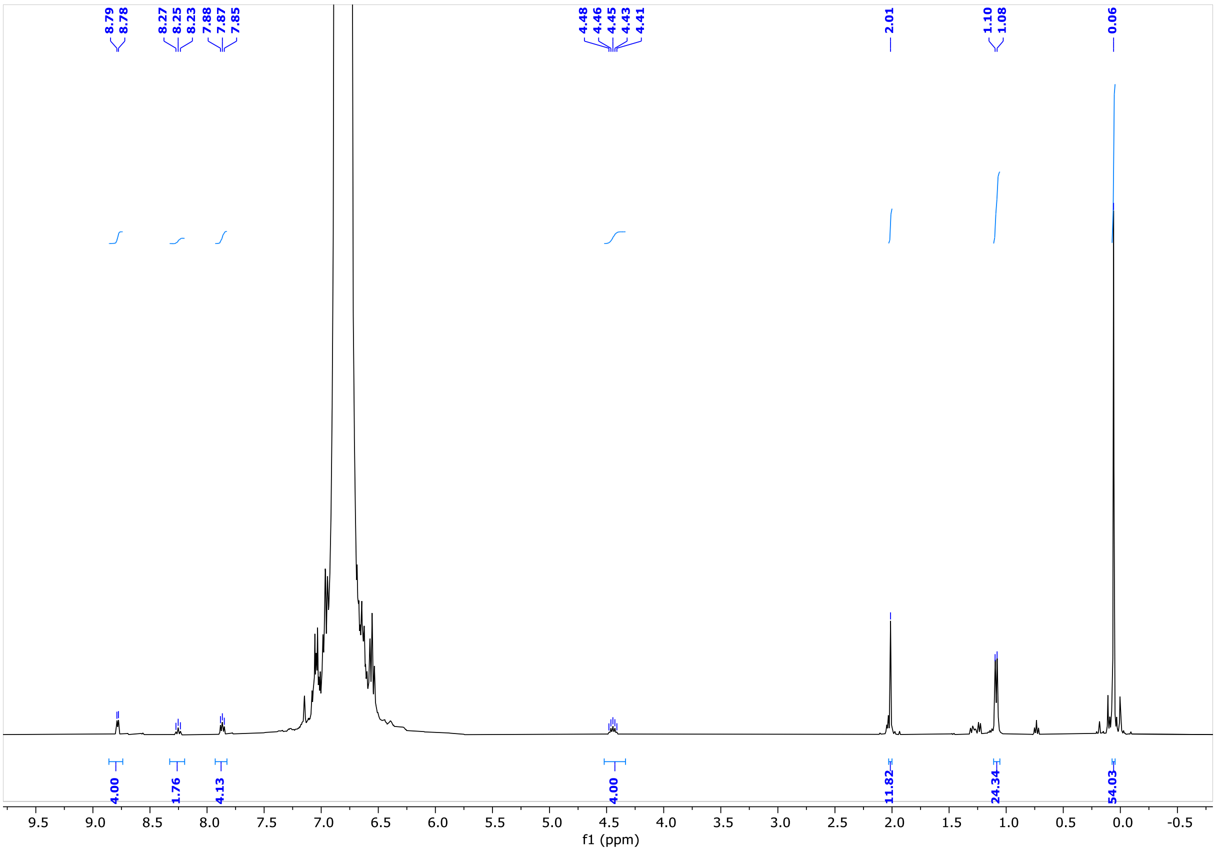

Supplementary Figure 23. ^{1}H NMR spectrum of **8** in a mixture of DFB and C_6D_6 at 300K.

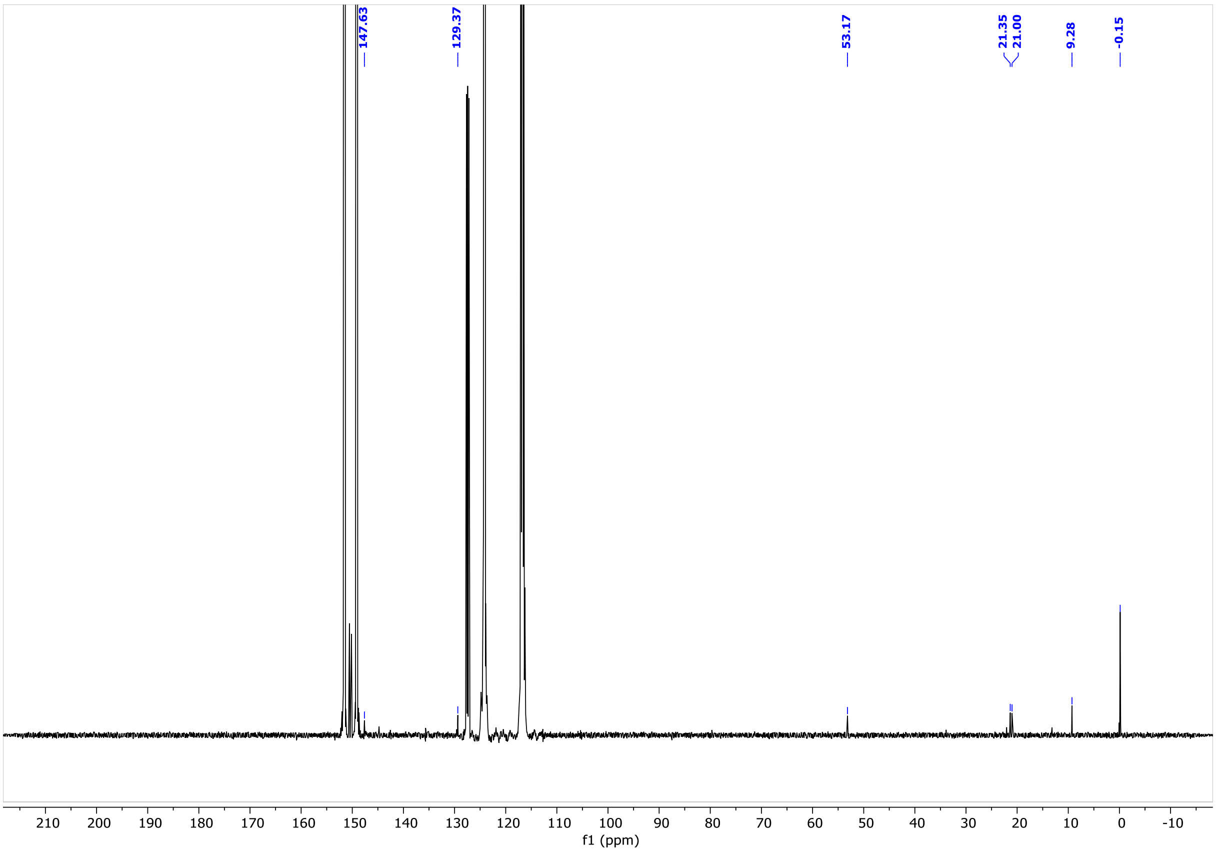

Supplementary Figure 24. ^{13}C{^{1}H} NMR spectrum of **8** in a mixture of DFB and C_6D_6 at 300K.

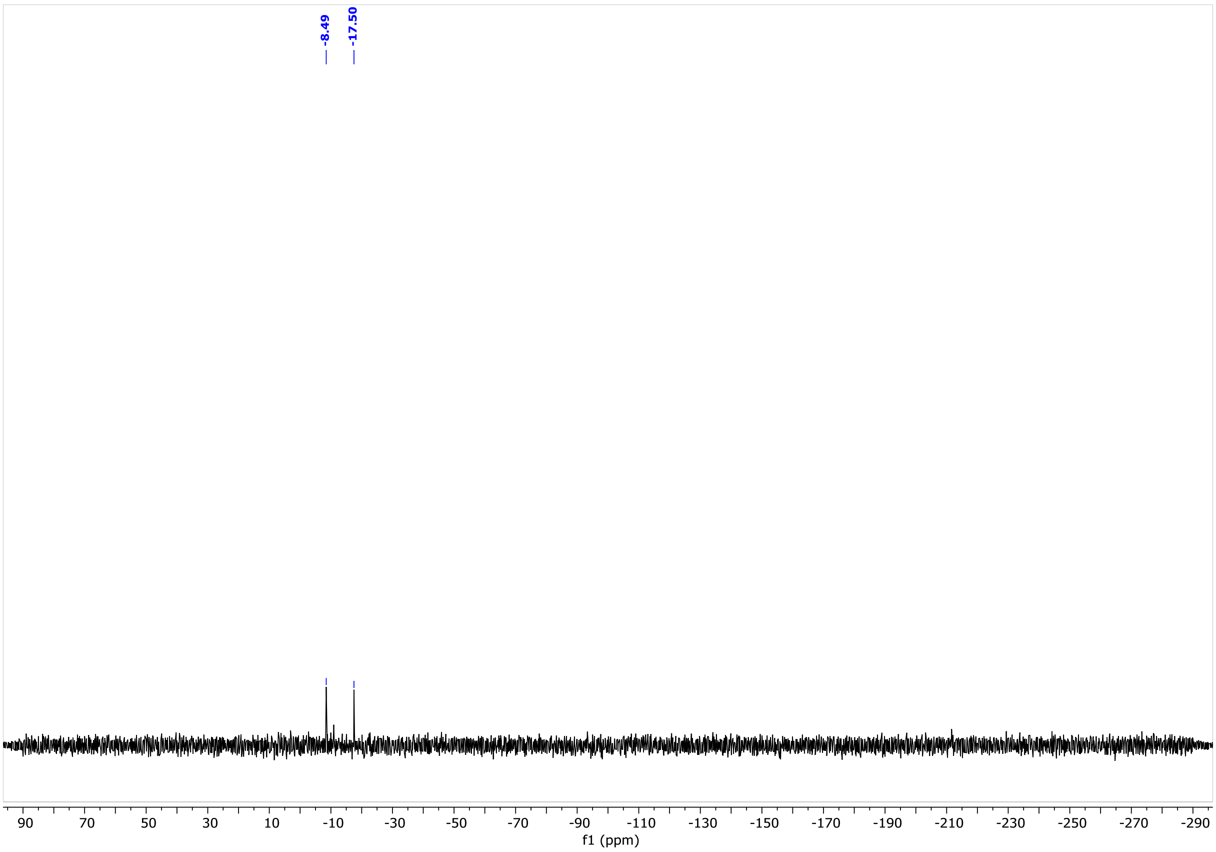

Supplementary Figure 25. ^{29}Si NMR spectrum of **8** in a mixture of DFB and C_6D_6 at 300K.

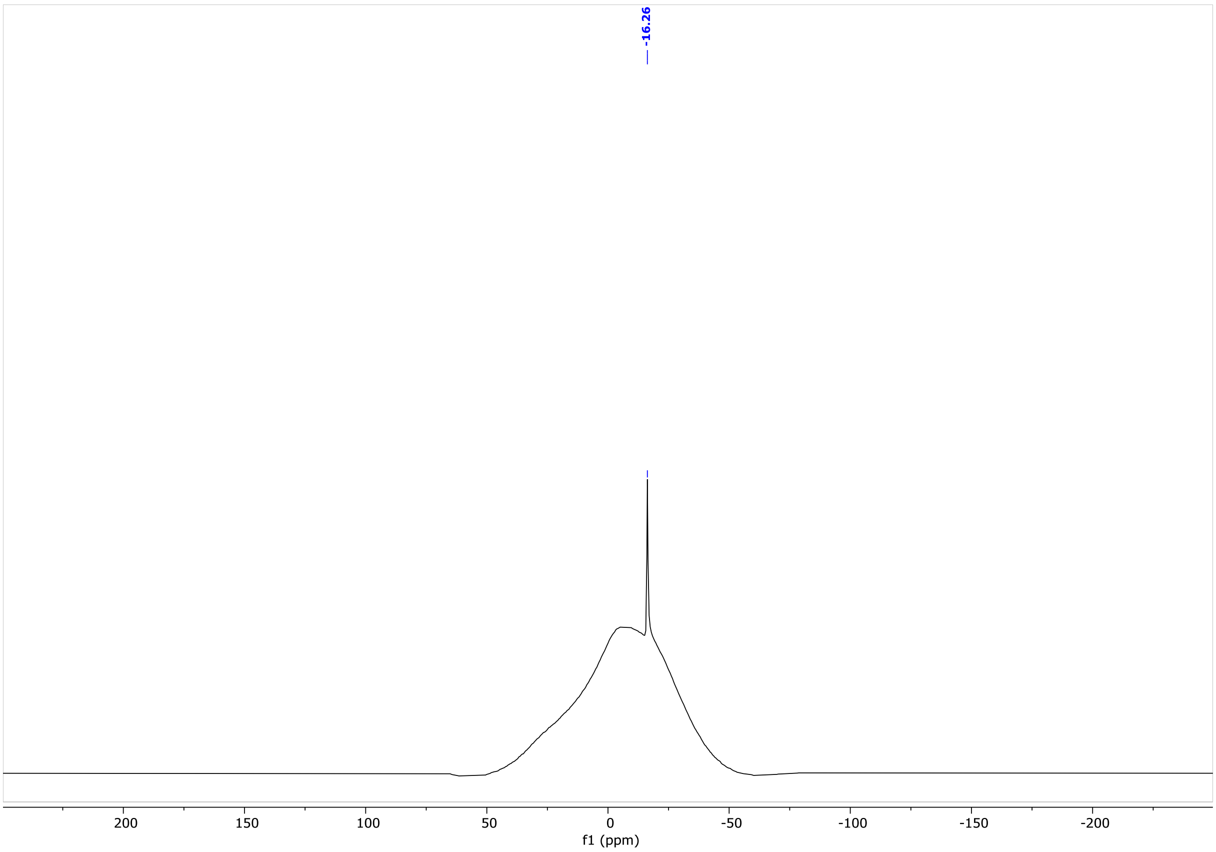

Supplementary Figure 26. ^{11}B NMR spectrum of **8** in a mixture of DFB and C_6D_6 at 300K.

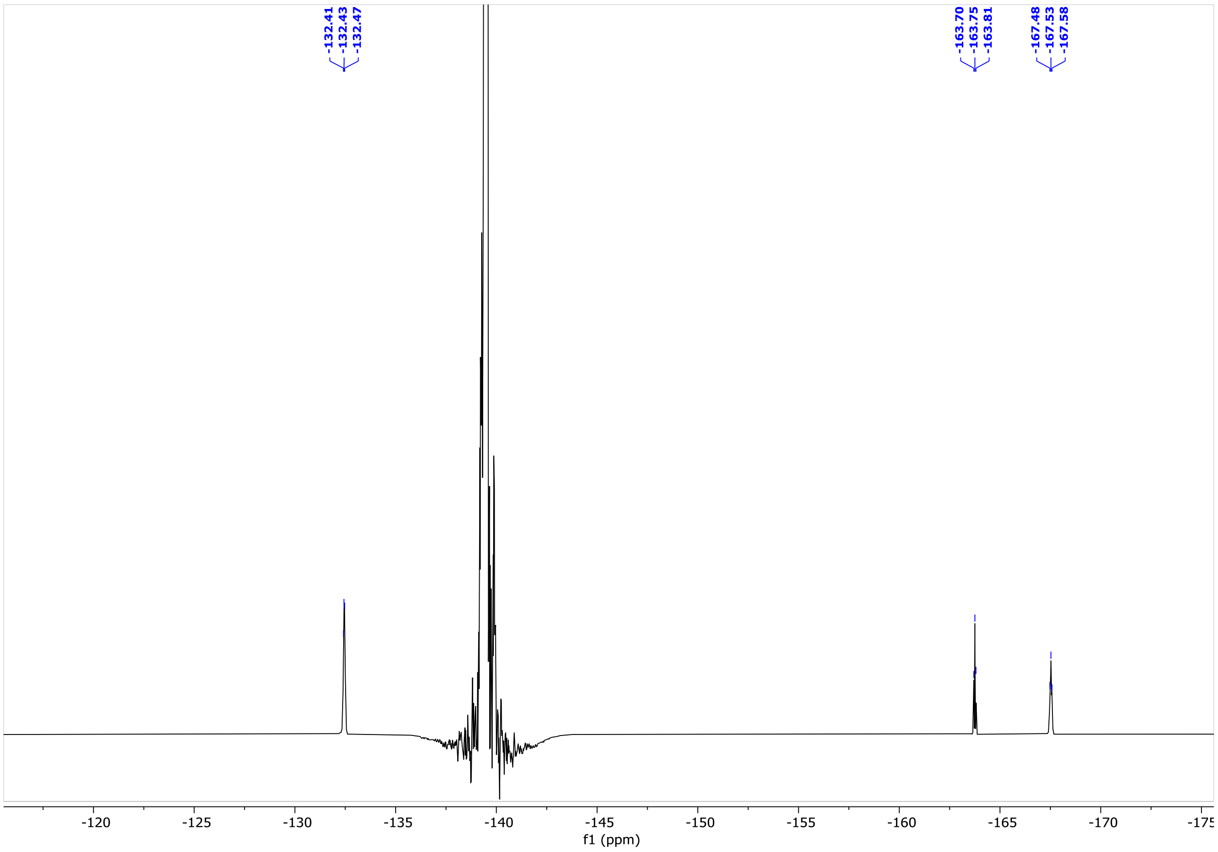

Supplementary Figure 27. ^{19}F NMR spectrum of **8** in a mixture of DFB and C_6D_6 at 300K.

1.2.11 Synthesis of 9a

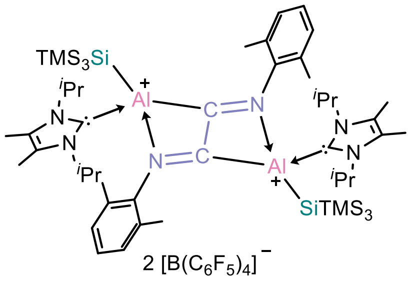

A mixture of **3** (25 mg, 0.011 mmol) and XylNC (2.9 mg, 0.022 mmol) was stirred in 1,2-difluorobenzene (0.4 mL) at room temperature for 10 minutes. The color of the solution changed from pale yellow to brown. The crude product was recrystallized in a mixture of 1,2-difluorobenzene and pentane at -30 °C to give **9a** as brown crystals (17.5 mg, 63% yield).

^{1}H NMR (400 MHz, DFB+C_6D_6): δ [ppm] 7.10 – 6.50 (m, 6H, C*H* in Ph, overlap with the DFB solvent), 3.93 – 3.70 (m, 4H, C*H*(CH_3)$_2$), 2.63 (s, 6H, C*H*$_3$ in XylNC), 2.31 (s, 6H, C*H*$_3$ in XylNC), 2.12 – 2.02 (m, 12H, *CH*$_3$ NHC), 1.32 (d, *J* = 6.8 Hz, 6H, CH(C*H*$_3$)$_2$), 1.22 (d, *J* = 6.8 Hz, 6H, CH(C*H*$_3$)$_2$), 1.09 (d, *J* = 6.9 Hz, 6H, CH(C*H*$_3$)$_2$), 0.78 (d, *J* = 6.7 Hz, 6H, CH(C*H*$_3$)$_2$), 0.13 (s, 54H, SiC*H*$_3$).

$^{13}C\{^1H\}$ NMR (101 MHz, DFB+C_6D_6): δ [ppm] 143.05, 130.96, 130.72, 130.70 (peaks from 144 – 130 ppm are assigned to the carbons in phenyl rings), carbon peaks for [*C*=*C* NHC] are overlapped with DFB solvent, 55.37 (*C*H(CH_3)$_2$), 54.73 (*C*H(CH_3)$_2$), 22.73, 22.63, 21.35, 21.06 (peaks from 23 – 21 ppm are assigned to CH($\mathit{C}H_3$)$_2$ and $\mathit{C}H_3$ in XylNC), 9.39 ($\mathit{C}H_3$ NHC), 9.35 ($\mathit{C}H_3$ NHC), 4.79 (Si$\mathit{C}H_3$).

^{29}Si NMR (79 MHz, DFB+C_6D_6): δ [ppm] -7.01 (*Si*CH_3).

No signal was found for aluminum-bonded silicon and carbene carbon atom, due to the quadrupolar momentum of the ^{27}Al nucleus.

^{11}B NMR (128 MHz, DFB+C_6D_6): δ [ppm] -16.25.

^{19}F NMR (377 MHz, DFB+C_6D_6): δ [ppm] -132.26 – -132.54 (m), -163.74 (t, *J* = 20.3 Hz), -167.49 (t, *J* = 18.4 Hz).

Elemental Analysis (%): Calcd: C 50.32, H 4.46, N 3.32; Found: C 50.13, H 4.39, N 3.14.

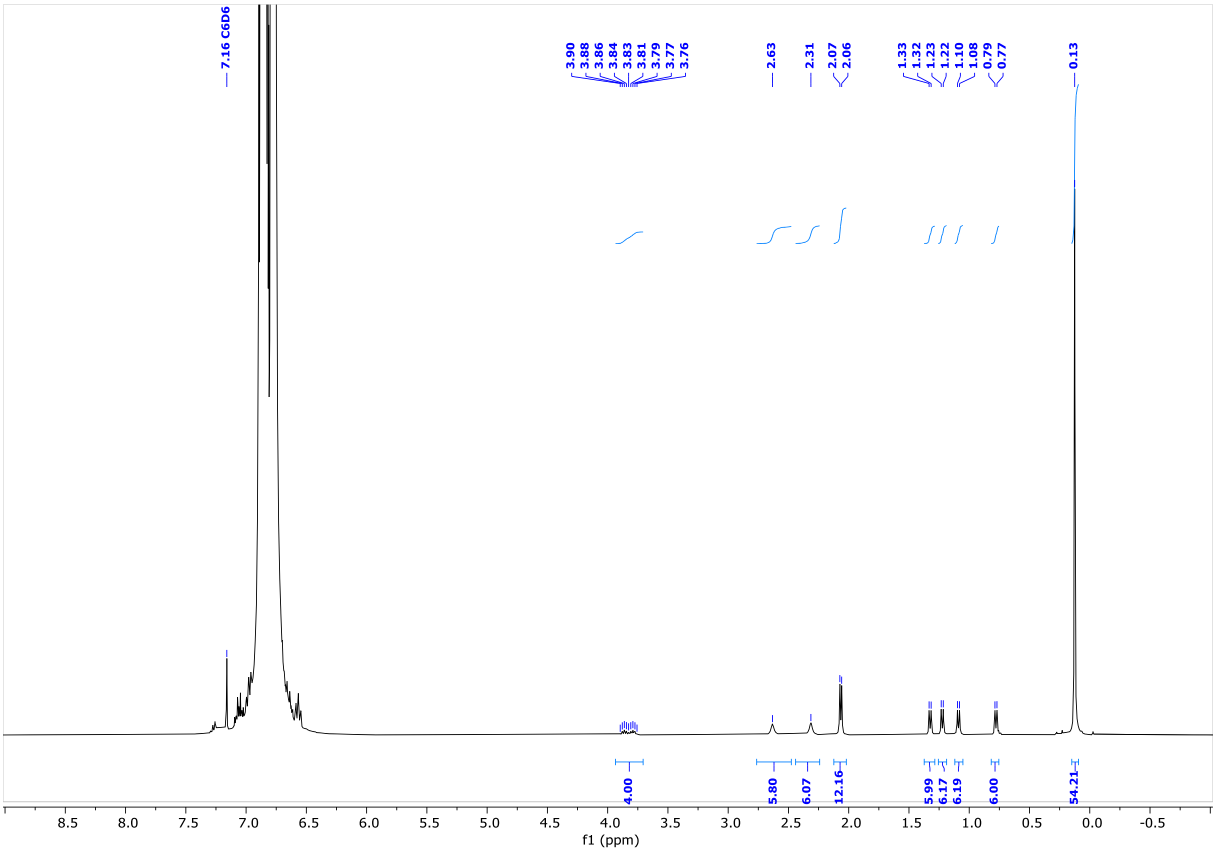

Supplementary Figure 28. ^1H NMR spectrum of **9a** in a mixture of DFB and C_6D_6 at 300K.

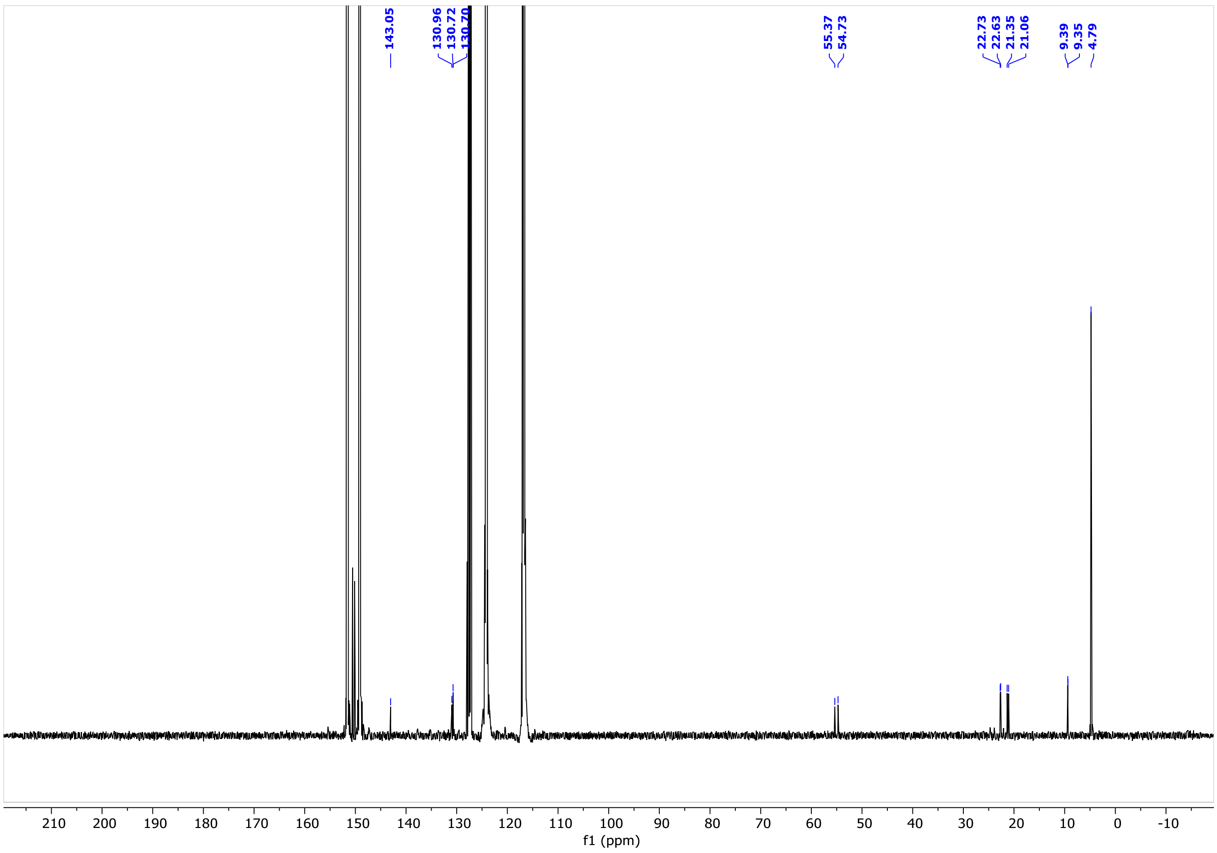

Supplementary Figure 29. $^{13}C\{^{1}H\}$ NMR spectrum of **9a** in a mixture of DFB and C_6D_6 at 300K.

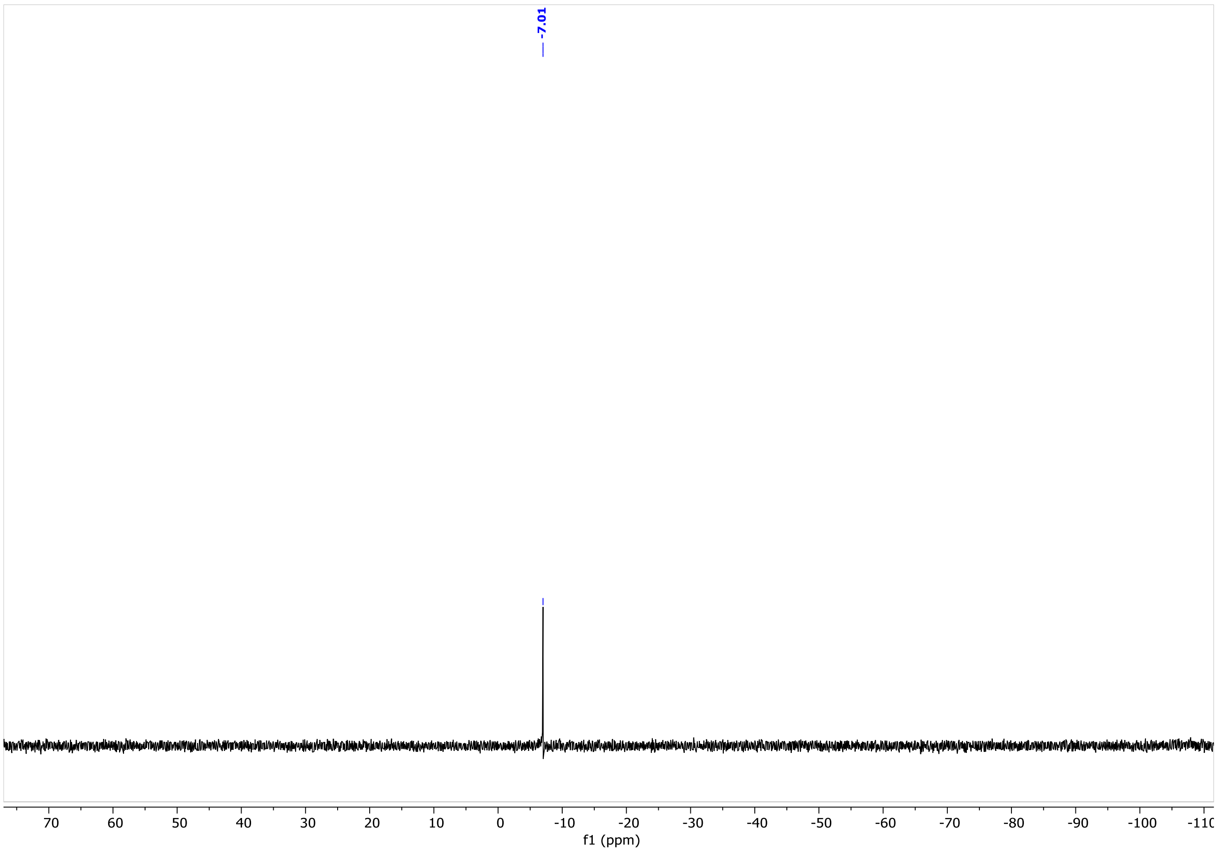

Supplementary Figure 30. ^{29}Si NMR spectrum of **9a** in a mixture of DFB and C_6D_6 at 300K.

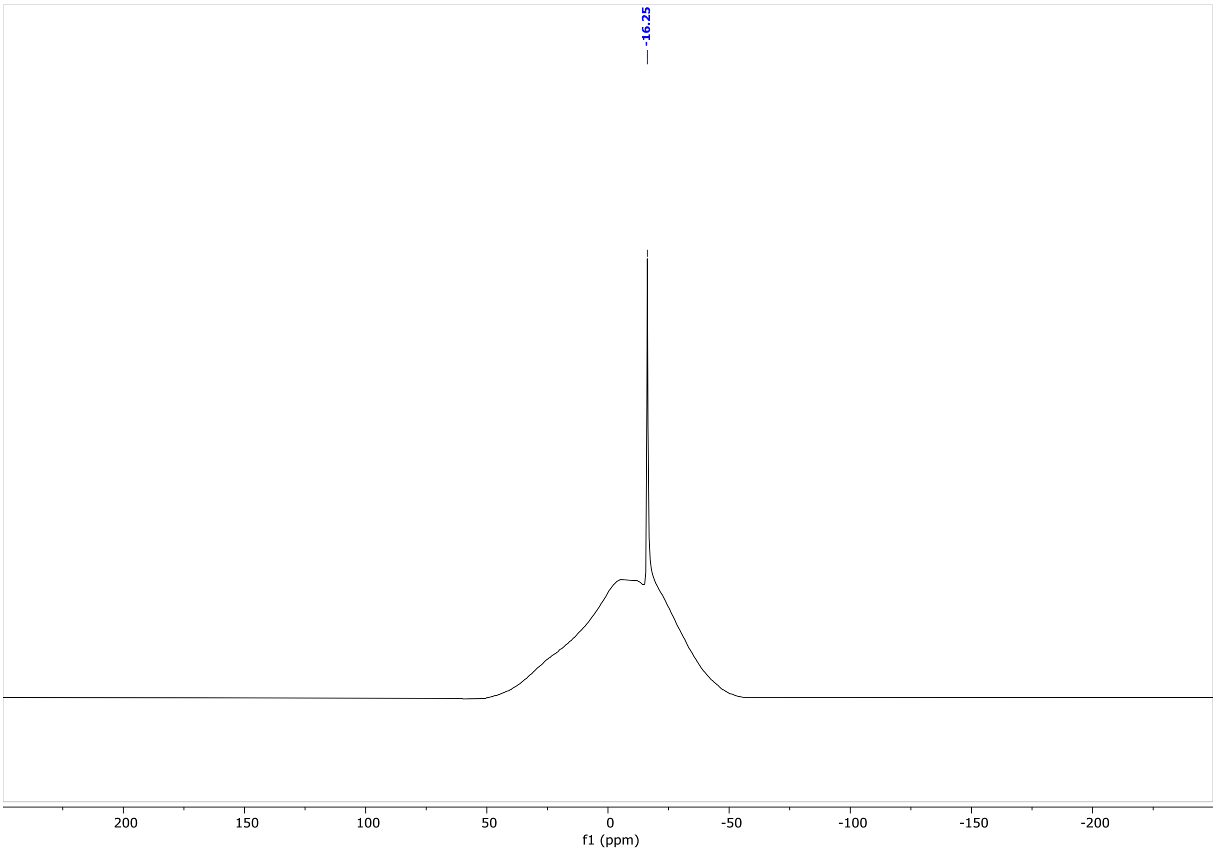

Supplementary Figure 31. ^{11}B NMR spectrum of **9a** in a mixture of DFB and C_6D_6 at 300K.

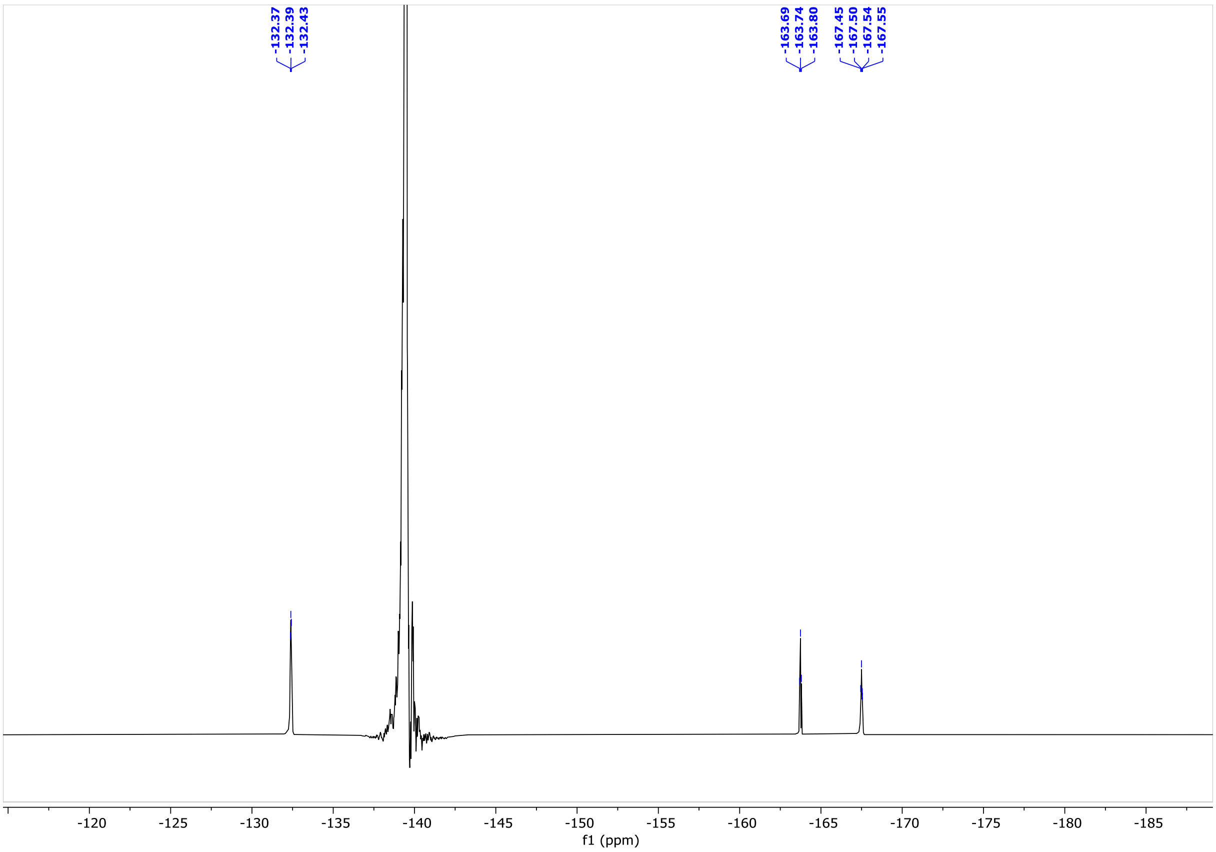

Supplementary Figure 32. ^{19}F NMR spectrum of **9a** in a mixture of DFB and C_6D_6 at 300K.

1.2.12 Synthesis of 9b

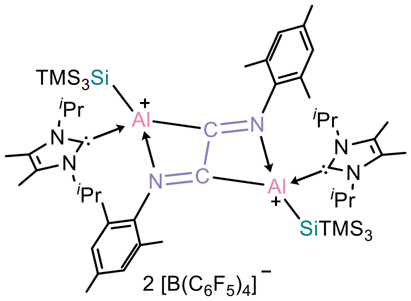

A mixture of **3** (20 mg, 0.0088 mmol) and MesNC (2.6 mg, 0.0177 mmol) was stirred in 1,2-difluorobenzene (0.4 mL) at room temperature for 10 minutes. The color of the solution changed from pale yellow to brown. The crude product was recrystallized in a mixture of 1,2-difluorobenzene and pentane at -30 °C to give **9b** as brown crystals (14 mg, 62% yield).

^{1}H NMR (400 MHz, DFB+C_6D_6): δ [ppm] 7.27 – 6.27 (m, 4H, C*H* in Ph, overlap with the DFB solvent), 4.00 – 3.88 (m, 2H, C*H*(CH_3)$_2$), 3.88 – 3.72 (m, 2H, C*H*(CH_3)$_2$), 2.62 (s, 6H, CH_3 in MesNC), 2.34 – 2.22 (m, 12H, CH_3 in MesNC), 2.12 – 2.03 (m, 12H, *CH$_3$* NHC), 1.35 (d, *J* = 6.8 Hz, 6H, CH(CH_3)$_2$), 1.25 (d, *J* = 6.8 Hz, 6H, CH(CH_3)$_2$), 1.10 (d, *J* = 6.8 Hz, 6H, CH(CH_3)$_2$), 0.77 (d, *J* = 6.7 Hz, 6H, CH(CH_3)$_2$), 0.14 (s, 54H, SiCH_3).

^{13}C{^{1}H} NMR (101 MHz, DFB+C_6D_6): δ [ppm] 142.19, 140.72, 130.87, 130.57 (peaks from 143 – 130 ppm are assigned to the carbons in phenyl rings), carbon peaks for [*C*=*C* NHC] are overlapped with DFB solvent, 55.30 (*C*H(CH_3)$_2$), 54.58 (*C*H(CH_3)$_2$), 22.62, 22.58, 21.17, 20.91, 19.59 (peaks from 23 – 19 ppm are assigned to CH(*C*H$_3$)$_2$ and *C*H$_3$ in MesNC), 9.39 (*C*H$_3$ NHC), 9.35 (*C*H$_3$ NHC), 4.71 (Si*C*H$_3$).

^{29}Si NMR (79 MHz, DFB+C_6D_6): δ [ppm] -7.06 (*Si*CH$_3$).

No signal was found for aluminum-bonded silicon and carbene carbon atom, due to the quadrupolar momentum of the ^{27}Al nucleus.

^{11}B NMR (128 MHz, DFB+C_6D_6): δ [ppm] -16.25.

^{19}F NMR (377 MHz, DFB+C_6D_6): δ [ppm] -132.28 – -132.55 (m), -163.79 (t, *J* = 20.2 Hz), -167.54 (t, *J* = 18.9 Hz).

Elemental Analysis (%): Calcd: C 50.70, H 4.57, N 3.28; Found: C 50.65, H 4.52, N 3.28.

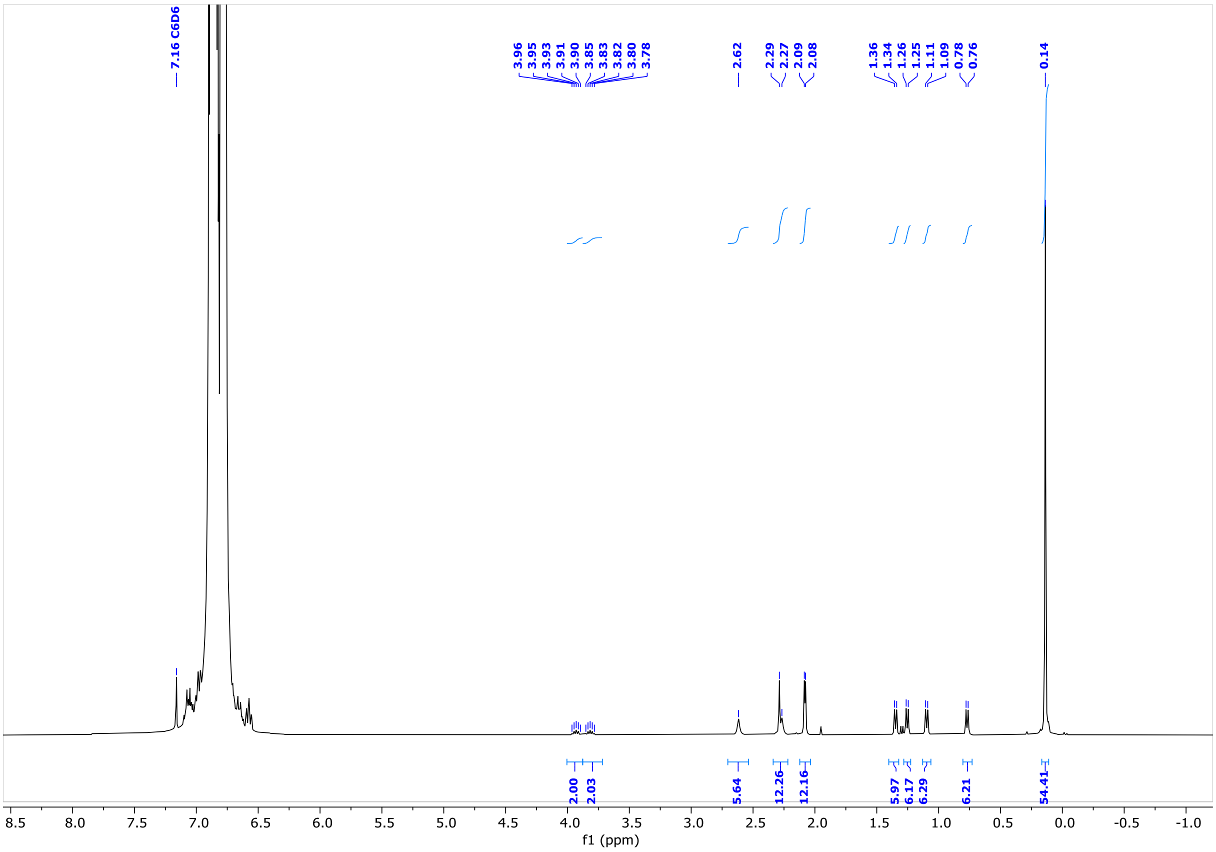

Supplementary Figure 33. ^{1}H NMR spectrum of **9b** in a mixture of DFB and C_6D_6 at 300K.

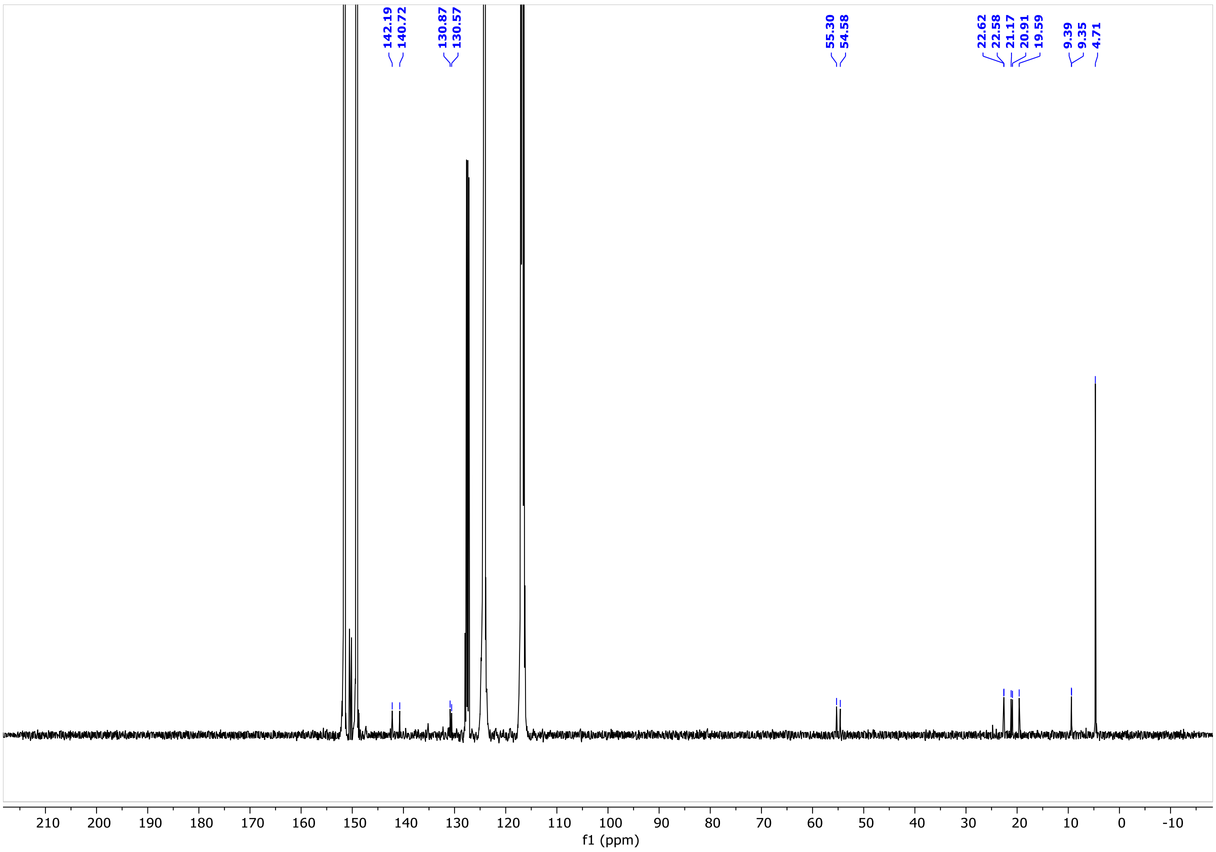

Supplementary Figure 34. $^{13}C\{^{1}H\}$ NMR spectrum of **9b** in a mixture of DFB and C_6D_6 at 300K.

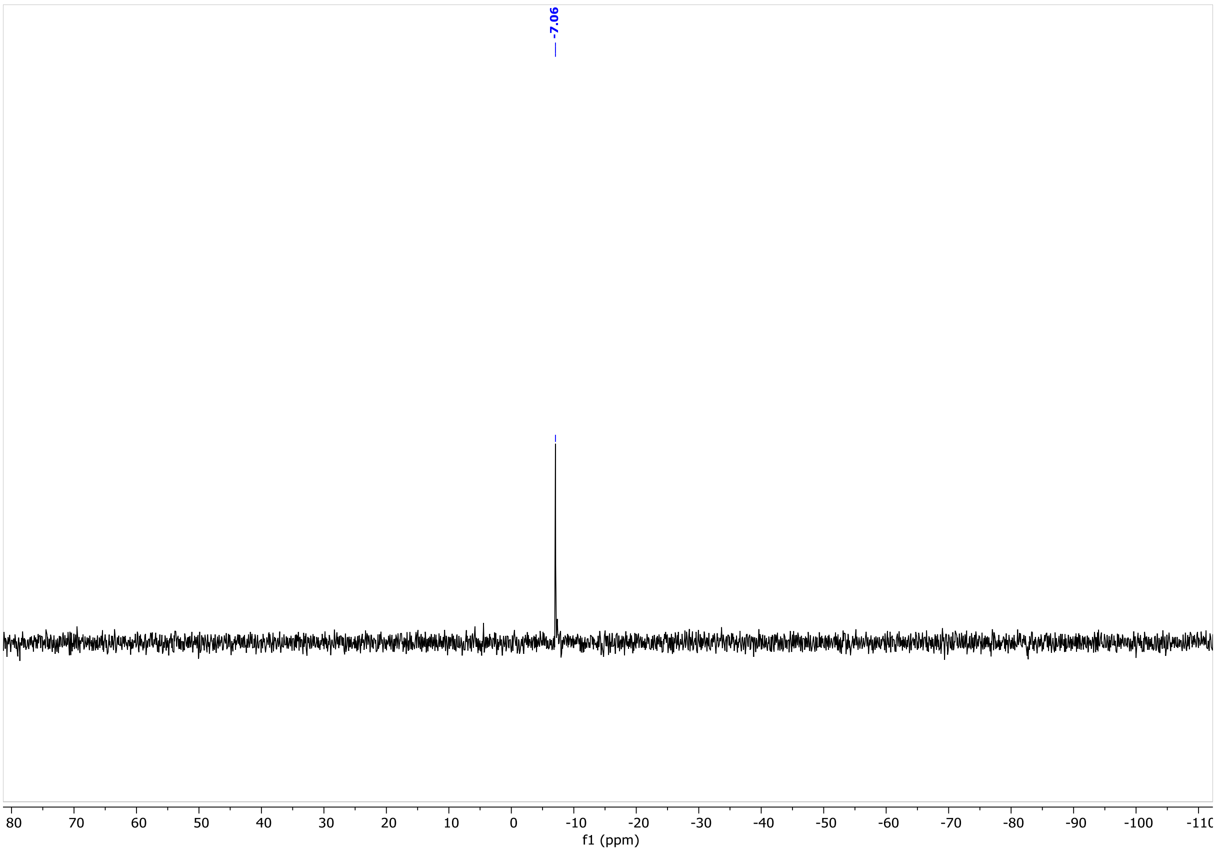

Supplementary Figure 35. ^{29}Si NMR spectrum of **9b** in a mixture of DFB and C_6D_6 at 300K.

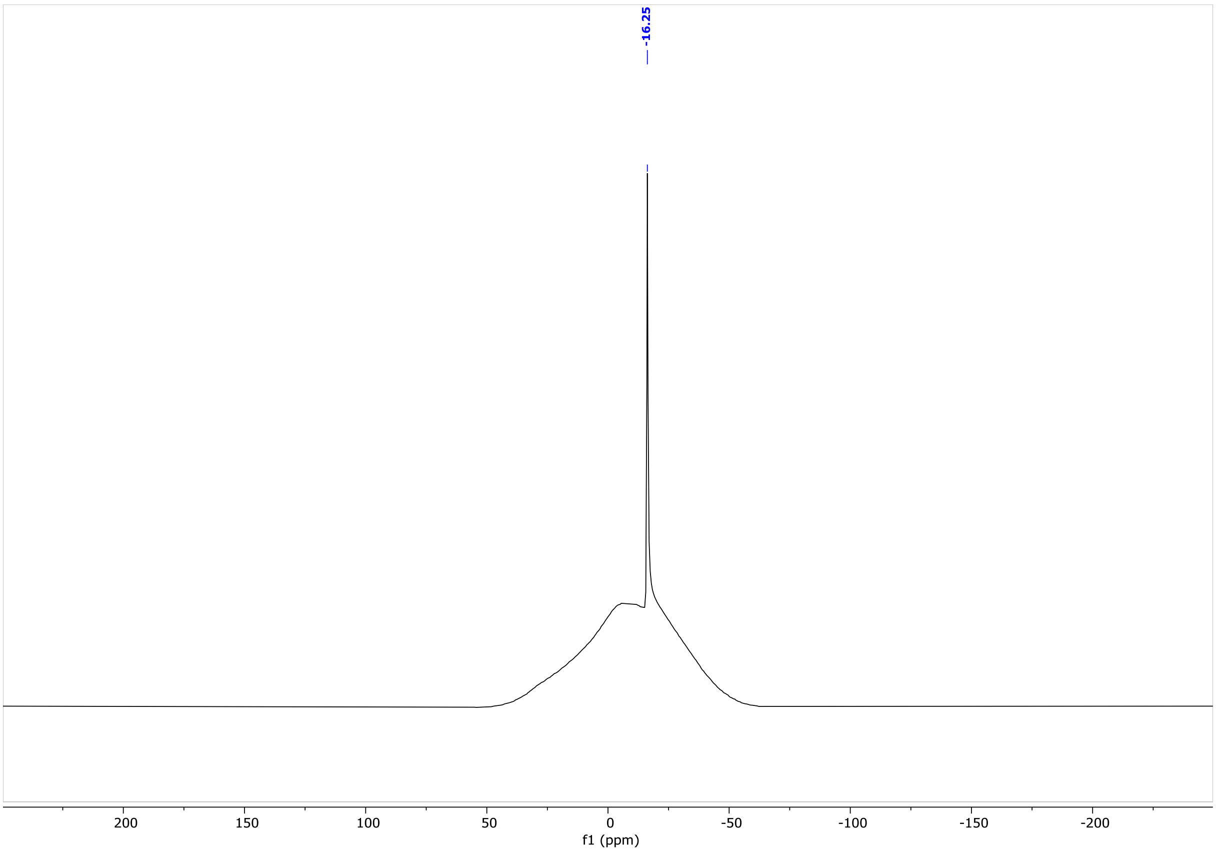

Supplementary Figure 36. ^{11}B NMR spectrum of **9b** in a mixture of DFB and C_6D_6 at 300K.

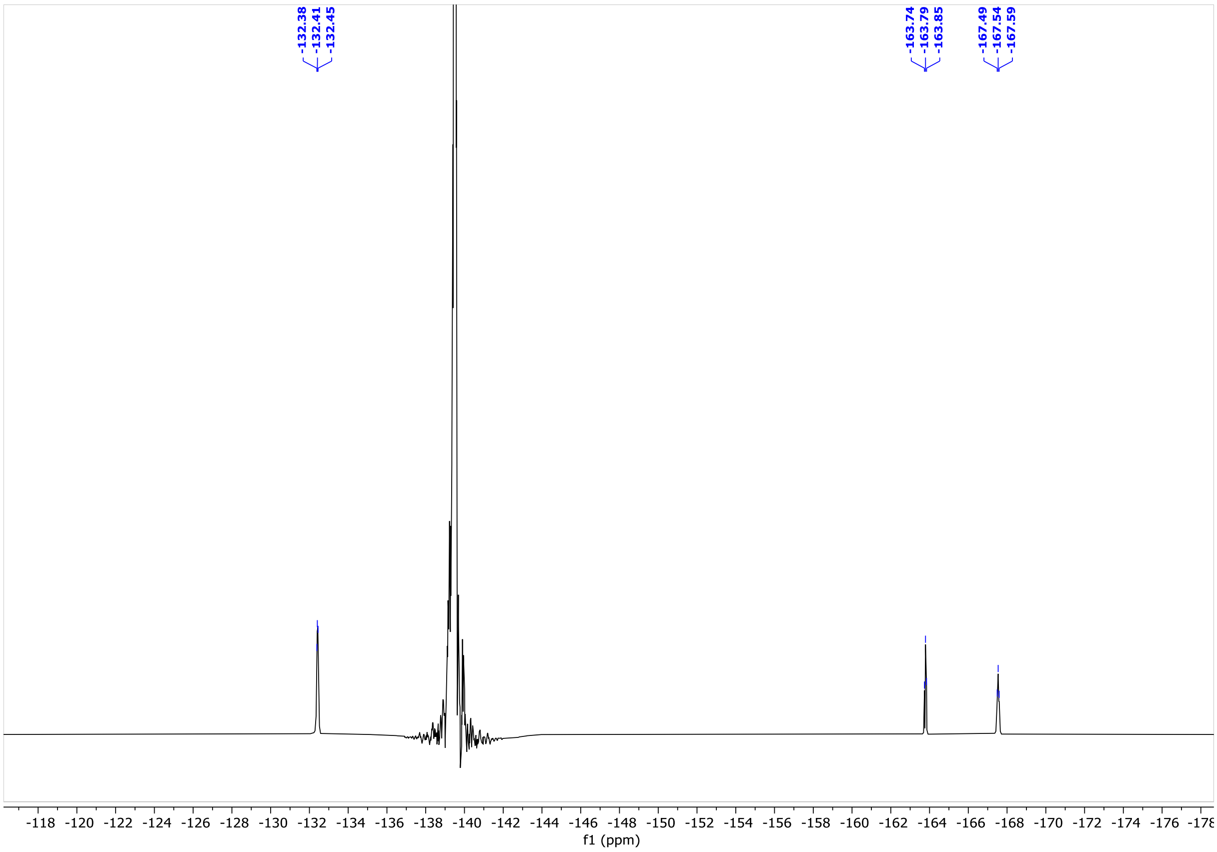

Supplementary Figure 37. ^{19}F NMR spectrum of **9b** in a mixture of DFB and C_6D_6 at 300K.

1.3 Electrochemical Measurements on Dialumene 1

We tested NaBArF, $[NBu_4][PF_6]$, $NaBPh_4$ and $[NBu_4][BPh_4]$ as electrolytes. It was found that dialumene **1** can readily react with NaBArF, indicating that $[BArF]^-$ is not a suitable counterion.

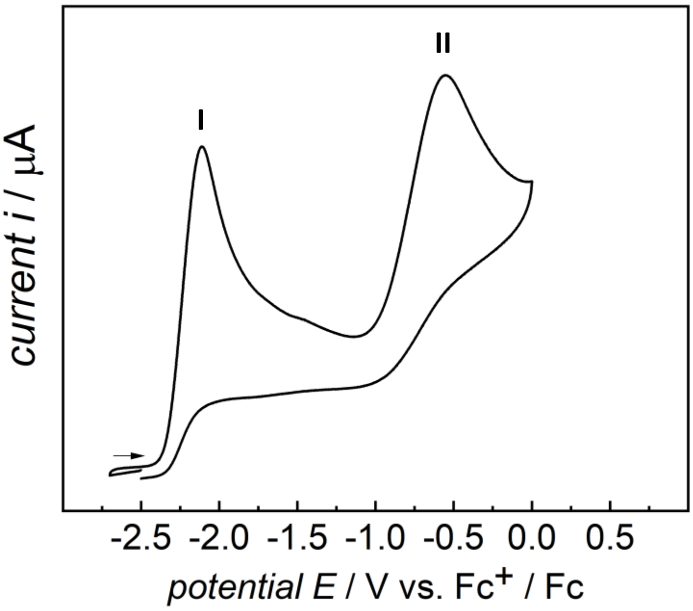

Supplementary Figure 38. Cyclic voltammogram of **1**, measured in a THF solution with ~ 0.1 M $[N(^{n}Bu)_4][PF_6]$ as electrolyte at room temperature. Scan rate = 0.05 V s^{-1}.

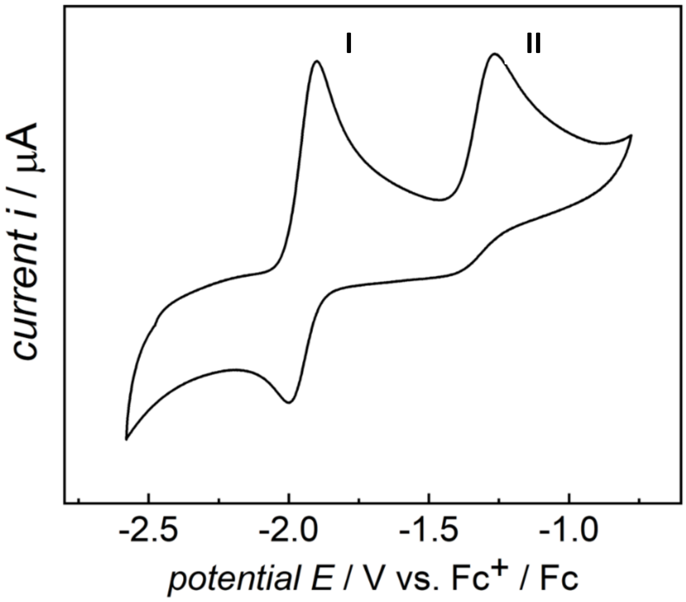

Supplementary Figure 39. Cyclic voltammogram of **1**, measured in a THF solution with ~ 0.1 M $NaBPh_4$ as electrolyte at room temperature. Scan rate = 0.05 V s^{-1}.

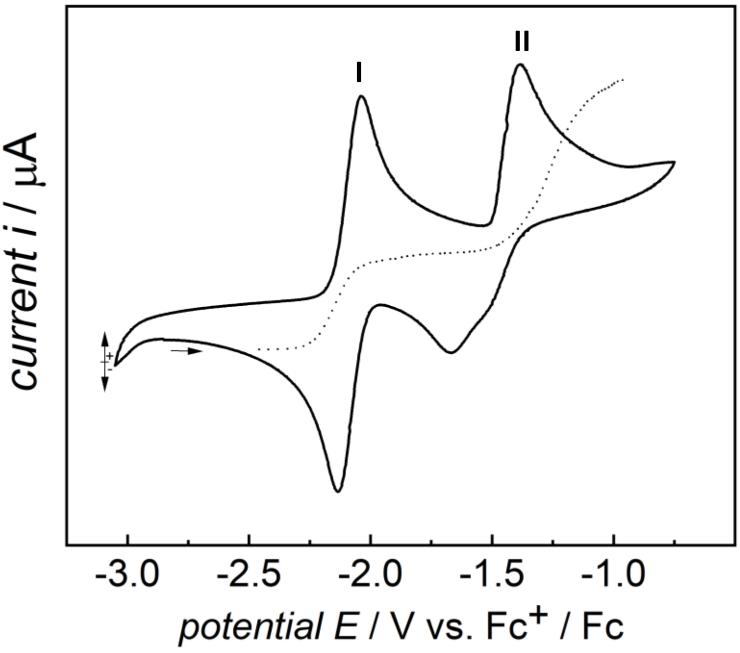

Supplementary Figure 40. Cyclic voltammogram of **1**, measured in a THF solution with ~ 0.1 M $[N(^{n}Bu)_4][BPh_4]$ as electrolyte at room temperature. Dashed line: linear sweep measurements. Scan rate = 0.05 V s^{-1}. Scan direction: - Ve to + Ve.

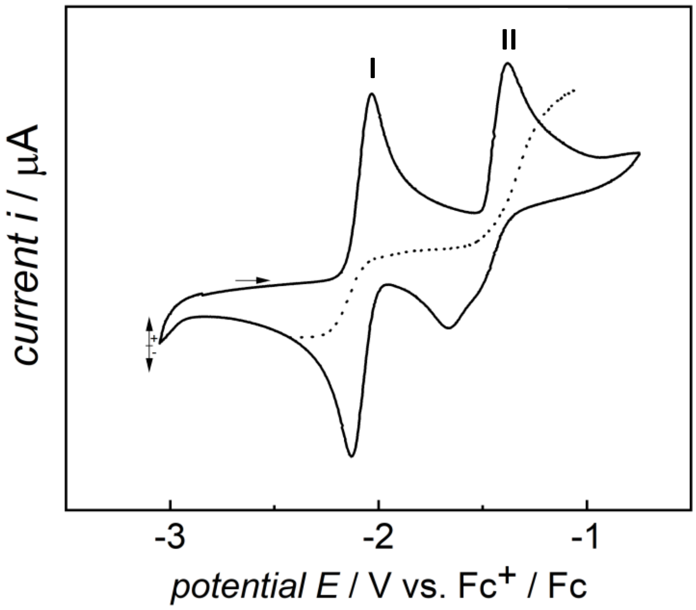

Supplementary Figure 41. Cyclic voltammogram of **1**, measured in a THF solution with ~ 0.1 M $[N(^{n}Bu)_4][BPh_4]$ as electrolyte at room temperature. Dashed line: linear sweep measurements. Scan rate = 0.05 V s^{-1}. Scan direction: + Ve to – Ve.

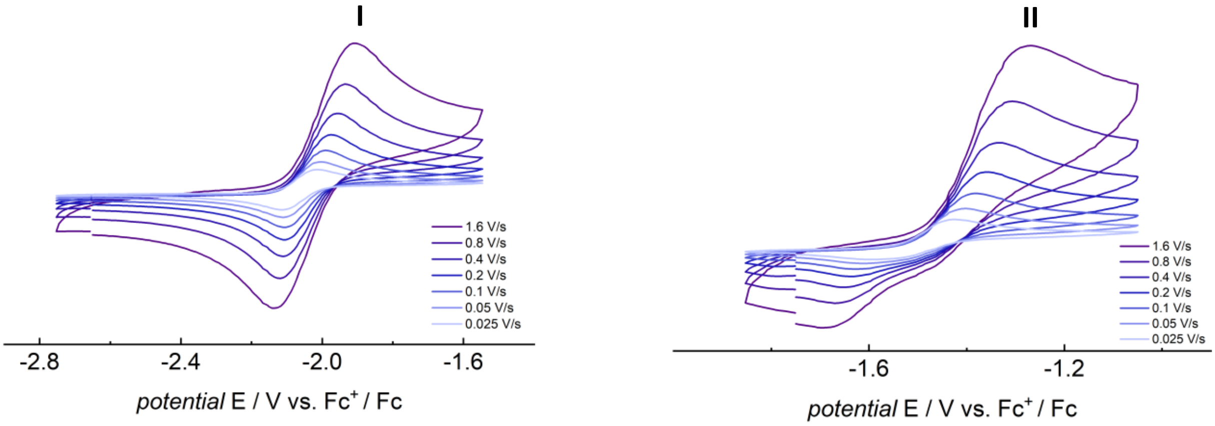

Supplementary Figure 42. Cyclic voltammograms of **1** in 1 mM THF solution at different scan rates, measured in a THF solution with ~ 0.1 M [N(^{n}Bu)$_4$][BPh$_4$] as electrolyte at room temperature.

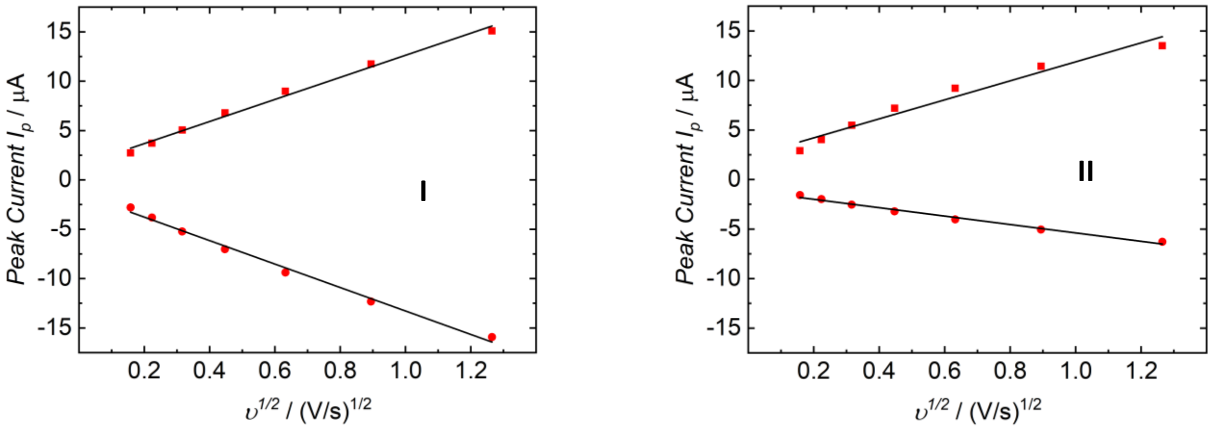

Supplementary Figure 43. Plot of cathodic (circles) and anodic (squares) currents versus the square root of the scan rates for **I** (left) and **II** (right) their corresponding linear fits (R^2 = 0.99 (cathodic) and 0.99 (anodic) for **I** and R^2 = 0.96 for **II**), measured in a THF solution with ~ 0.1 M [N(^{n}Bu)$_4$][BPh$_4$] as electrolyte at room temperature.

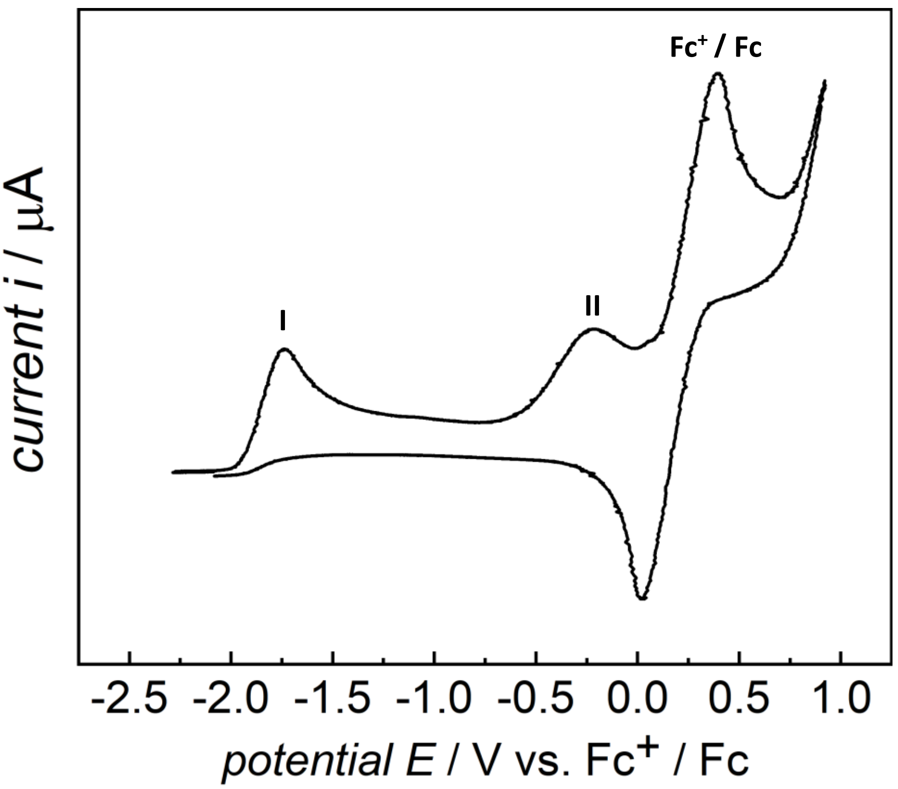

Supplementary Figure 44. Overview of the cyclic voltammogram of **1** with an additional showing of the Fc+ / Fc reference, measured in a THF solution with ~ 0.1 M $[N(^nBu)_4][BPh_4]$ as electrolyte at room temperature.

1.4 Redox-Reversible Transformation

1.4.1 Synthesis of 2 via the reduction of 3

A mixture of the dication **3** (23 mg, 0.01 mmol) and KC_8 (1.5 mg, 0.011 mmol) was stirred in difluorobenzene (0.5 mL) at room temperature for 10 minutes. The color of the solution immediately changed from pale yellow to dark purple. All volatiles were dried under reduced pressure, and the residue was washed with benzene and pentane to give a purple powder. The crude product was recrystallized in a mixture of fluorobenzene and pentane at -30 °C to give **2** as a purple solid (8.9 mg, 56% yield).

1.4.2 Synthesis of 1 via the reduction of 2 or 3

Method A: A mixture of the radical cation **2** (10 mg, 0.006 mmol) and KC_8 (0.85 mg, 0.006 mmol) was stirred in fluorobenzene (0.3 mL) at room temperature for 10 minutes. The color of the solution immediately changed from dark purple to dark blue. The regeneration of dialumene **1** was evidenced by UV-Vis. However, the regenerated dialumene **1** rapidly reacted with the in situ–formed $KBAr_4^F$, preventing its isolation.

Method B: A mixture of the dication **3** (23 mg, 0.01 mmol) and KC_8 (3 mg, 0.022 mmol) was stirred in difluorobenzene (0.5 mL) at room temperature for 10 minutes. The color of the solution changed sequentially from pale yellow to dark purple, and finally to dark blue. The regeneration of dialumene **1** was evidenced by UV-Vis. However, the regenerated dialumene **1** rapidly reacted with the in situ–formed $KBAr_4^F$, preventing its isolation.

As shown below, the characteristic absorption band at 598 nm—assigned to dialumene **1**—appears immediately upon reduction but diminishes continuously over time. This behavior is consistent with rapid reaction of **1** with the in situ–generated $KBAr_4^F$.

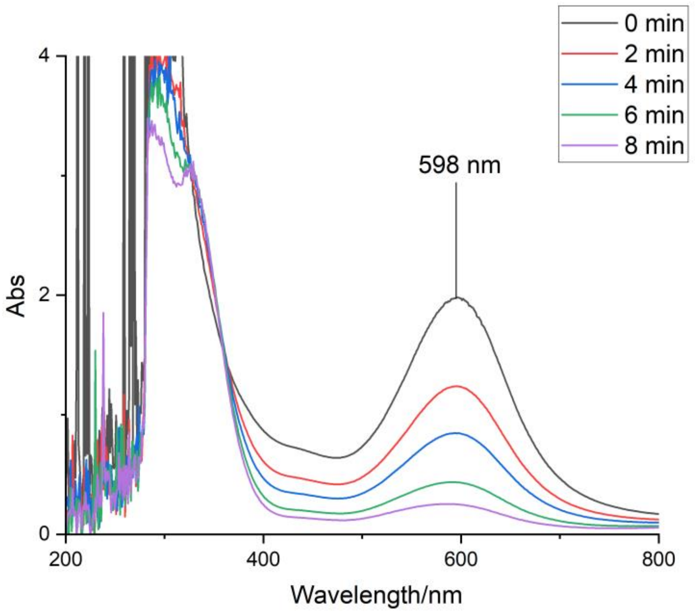

Supplementary Figure 45. UV–vis of the regenerated dialumene **1**

1.4.3 Synthesis of 2 via the reaction of 1 and 3

A mixture of the dication **3** (23 mg, 0.01 mmol) and dialumene **1** (9.1 mg, 0.01 mmol) was stirred in fluorobenzene (0.4 mL) at room temperature for 10 minutes. The color of the solution immediately changed from dark blue to dark purple. The crude product was recrystallized in a mixture of fluorobenzene and pentane at -30 °C to give the radical cation **2** as dark purple crystals (24 mg, 75% yield).

1.5 Reactivity of Radical Cation 2

Exposure a fluorobenzene solution of **2** (20 mg) to N_2O (1 bar) at room temperature resulted in an instant color change from dark purple to colorless, with compound **6** precipitated as colorless crystals.

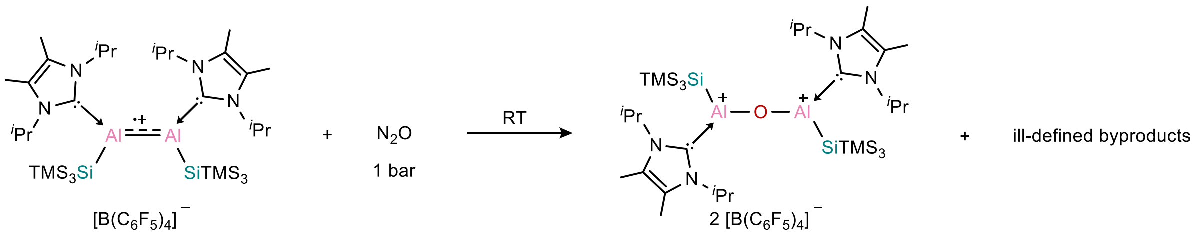

Supplementary Figure 46. Reaction of radical cation **2** with N_2O.

2 Single Crystal X-Ray Structure Determination

Single crystal diffraction data were collected on a single-crystal X-ray diffractometer equipped with a Charge-Integrating Pixel Array Detector (Brucker Photon-II), a Microfocus X-Ray Source with a CuK_α ($\lambda = 1.54178$) or a Turbo X-Ray Source rotating anode with MoK_α radiation ($\lambda = 0.71073$ Å) and a Helios optic using the APEX4 software package[4] on single crystals coated with Fomblin®Y as perfluorinated ether. The single crystals were picked on a micro sampler, transferred to the diffractometer, and measured frozen under a stream of cold nitrogen (100 K). A matrix scan was used to determine the initial lattice parameters. Reflections were merged and corrected for Lorenz and polarization effects, scan speed, and background using SAINT.[5] Absorption corrections, including odd and even ordered spherical harmonics were performed using SADABS.[5] Space group assignments were based upon systematic absences, E statistics, and successful refinement of the structures. Structures were solved by direct methods with the aid of successive difference Fourier maps and were refined against all data using the APEX IV software in conjunction with SHELXL-2014[6] and SHELXLE.[7] H atoms were placed in calculated positions and refined using a riding model, with methylene and aromatic C–H distances of 0.99 and 0.95 Å, respectively, and Uiso(H) = 1.2·Ueq(C). Non-hydrogen atoms were refined with anisotropic displacement parameters. Full-matrix least-squares refinements were carried out by minimizing Σw(Fo2-Fc2)2 with the SHELXL weighting scheme.[8] Neutral atom scattering factors for all atoms and anomalous dispersion corrections for the non-hydrogen atoms were taken from International Tables for Crystallography.[9] The images of the crystal structures were generated by Mercury.[10] The CCDC numbers 2490063-2490073 contain the supplementary crystallographic data for the structures **1-9**. These data can be obtained free of charge from the Cambridge Crystallographic Data Centre via https://www.ccdc.cam.ac.uk/structures/.

Supplementary Table 1 Crystallographic details

	**1**	**2**	**3**
**CCDC-Number**	2490063	2490064	2490065
Chemical formula	$C_{40}H_{94}Al_2N_4Si_8$	$C_{64}H_{94}Al_2N_4Si_8BF_{20}$	$[C_{88}H_{94}Al_2N_4Si_8B_2F_{40}]\cdot 2\ C_6H_4F_2$
M_r	909.87	1588.92	2496.15
Crystal system, space group	triclinic, $P\bar{1}$	orthorhombic, $Pna2_1$	triclinic, $P\bar{1}$
Temperature (K)	100	100	100
a (Å), α(°)<br>*b (Å)*, β(°)<br>*c (Å)*, γ(°)	17.1933(8), 94.023(2)<br>18.4876(8), 100.360(2)<br>18.6096(8), 99.023(2)	24.9426(14), 90<br>17.7212(13), 90<br>18.5034(14), 90	13.8747(7), 86.770(2)<br>15.6903(8), 67.135(2)<br>15.8458(7), 64.678(2)
V ($Å^3$)	5717.3(4)	8178.7(10)	2848.0(2)
Z	4	4	1
F(000)	2000	3316	1274
D_x (g/cm^3)	1.057	1.290	1.455
Radiation type	Mo *K*α	Mo *K*α	Mo *K*α
μ (mm^{-1})	0.247	0.237	0.229
θ range (°) for cell meas.	2.40–25.65	2.20–25.16	2.64–25.63
Crystal size (mm)	0.193 × 0.188 × 0.102	0.294 × 0.201 × 0.124	0.302 × 0.256 × 0.195
Diffractometer	Bruker Photon CMOS	Bruker Photon CMOS	Bruker Photon CMOS
Radiation source	TXS rotating anode	TXS rotating anode	TXS rotating anode
Monochromator	Helios optic	Helios optic	Helios optic
Absorption correction	Multi-scan	Multi-scan	Multi-scan
T_{min}, T_{max}	0.692, 0.745	0.701, 0.745	0.719, 0.745
$θ_{max}$ (°)	25.688	25.667	25.675
Range of *h*, *k*, *l*	*h* = -20→20, *k* = -22→22, *l* = -22→22	*h* = -30→26, *k* = -21→21, *l* = -22→22	*h* = -16→16, *k* = -19→19, *l* = -19→19
Refinement method	Full-matrix least-squares on F^2	Full-matrix least-squares on F^2	Full-matrix least-squares on F^2
Data/restraints/ parameters	21670/28/1043	15448/1/922	10767/0/736
Goodness-of-fit on F^2	1.194	1.055	1.031
Final *R* indices (I>2σ(I))	R_1 = 0.0770, wR_2 = 0.1854	R_1 = 0.0453, wR_2 = 0.1032	R_1 = 0.0354, wR_2 = 0.0956
$Δρ_{max}$, $Δρ_{min}$ (e $Å^{-3}$)	1.374, -0.487	0.302, -0.269	0.471, -0.588

	**4a**	**4b**	**5**
**CCDC-Number**	2490066	2490067	2490068
Chemical formula	$C_{102}H_{114}Al_2N_8Si_8B_2F_{40}$	$[C_{98}H_{104}Al_2N_6Si_8B_2F_{40}]\cdot 2\ C_6H_4F_2$	$C_{102}H_{104}Al_2N_6Si_8B_2F_{40}$
M_r	2512.31	2654.35	2474.21
Crystal system, space group	triclinic, $P\bar{1}$	triclinic, $P\bar{1}$	monoclinic, *C*2/*c*
Temperature (K)	100	100	100
a (Å), α(°)<br>*b (Å)*, β(°)<br>*c (Å)*, γ(°)	13.8346(12), 67.681(3)<br>15.1115(13), 77.665(3)<br>17.1149(16), 82.374(3)	13.7351(9), 112.951(2)<br>15.5961(9), 96.172(2)<br>16.8845(10), 106.465(2)	18.2695(13), 90<br>21.4405(14), 98.617(2)<br>33.162(2), 90
V ($Å^3$)	3228.3(5)	3095.1(3)	12843.0(15)
Z	1	1	4
F(000)	1290	1358	5070.9
D_x (g/cm^3)	1.292	1.424	1.280
Radiation type	Mo $K\alpha$	Mo $K\alpha$	Mo $K\alpha$
μ (mm^{-1})	0.199	0.215	0.198
θ range (°) for cell meas.	2.49–25.65	2.35–25.65	2.48–25.49
Crystal size (mm)	0.254 × 0.205 × 0.189	0.384 × 0.184 × 0.09	0.264 × 0.186 × 0.125
Diffractometer	Bruker Photon CMOS	Bruker Photon CMOS	Bruker Photon CMOS
Radiation source	TXS rotating anode	TXS rotating anode	TXS rotating anode
Monochromator	Helios optic	Helios optic	Helios optic
Absorption correction	Multi-scan	Multi-scan	Multi-scan
T_{min}, T_{max}	0.715, 0.745	0.721, 0.745	0.707, 0.745
θ_{max} (°)	25.649	25.676	25.720
Range of *h*, *k*, *l*	*h* = -16→16, *k* = -18→18, *l* = -20→20	*h* = -16→16, *k* = -18→18, *l* = -20→20	*h* = -22→22, *k* = 0→26, *l* = 0→40
Refinement method	Full-matrix least-squares on F^2	Full-matrix least-squares on F^2	Full-matrix least-squares on F^2
Data/restraints/ parameters	12201/0/747	11696/0/794	12192/0/736
Goodness-of-fit on F^2	1.027	1.038	1.060
Final *R* indices (I>2σ(I))	R_1 = 0.0372, wR_2 = 0.1039	R_1 = 0.0359, wR_2 = 0.1093	R_1 = 0.0389, wR_2 = 0.1158
$\Delta\rho_{max}$, $\Delta\rho_{min}$ (e $Å^{-3}$)	0.371, -0.293	0.466 -0.433	0.3353, -0.302

	**6**	**7**	**8**
**CCDC-Number**	2490069	2490070	2490071
Chemical formula	$C_{88}H_{94}Al_2N_4Si_8B_2F_{40}O$	$[C_{98}H_{104}Al_2N_6Si_8B_2F_{40}O_3]\cdot C_6H_4F_2$	$[C_{98}H_{104}Al_2N_6Si_8B_2F_{40}O_3]\cdot 2\ C_6H_4F_2$
M_r	2283.97	2588.26	2702.35
Crystal system, space group	monoclinic, $P2_1/c$	triclinic, $P\bar{1}$	triclinic, $P\bar{1}$
Temperature (K)	100	100	100
a (Å), α(°)<br>*b (Å)*, β(°)<br>*c (Å)*, γ(°)	13.8821(13), 90<br>13.1353(12), 95.854(3)<br>28.352(3), 90	14.1780(14), 86.669(3)<br>16.1350(16), 83.107(3)<br>28.714(3), 68.809(3)	13.9904(18), 113.750(4)<br>15.845(2), 96.289(4)<br>16.967(2), 106.189(5)
V (Å^3)	5142.9(8)	6079.4(10)	3199.1(7)
Z	2	2	1
F(000)	2332	2648	1382
D_x (g/cm^3)	1.475	1.414	1.403
Radiation type	Mo $K\alpha$	Mo $K\alpha$	Mo $K\alpha$
μ (mm^{-1})	0.241	0.217	0.212
θ range (°) for cell meas.	2.21–22.02	2.39–24.17	2.30–25.65
Crystal size (mm)	0.256 × 0.214 × 0.105	0.195 × 0.156 × 0.106	0.203 × 0.185 × 0.124
Diffractometer	Bruker Photon CMOS	Bruker Photon CMOS	Bruker Photon CMOS
Radiation source	TXS rotating anode	TXS rotating anode	TXS rotating anode
Monochromator	Helios optic	Helios optic	Helios optic
Absorption correction	Multi-scan	Multi-scan	Multi-scan
T_{min}, T_{max}	0.628, 0.745	0.660, 0.745	0.707, 0.745
θ_{max} (°)	25.754	25.776	25.668
Range of h, k, l	h = -16→16, k = -15→16, l = -34→33	h = -17→17, k = -19→19, l = -35→35	h = -17→17, k = -19→19, l = -20→20
Refinement method	Full-matrix least-squares on F^2	Full-matrix least-squares on F^2	Full-matrix least-squares on F^2
Data/restraints/ parameters	9743/0/670	23200/14/1534	12056/0/805
Goodness-of-fit on F^2	1.046	1.034	1.031
Final R indices (I>2σ(I))	R_1 = 0.0524, wR_2 = 0.1258	R_1 = 0.0595, wR_2 = 0.1722	R_1 = 0.0537, wR_2 = 0.1704
$\Delta\rho_{max}$, $\Delta\rho_{min}$ (e Å^{-3})	0.401, -0.362	1.630, -0.552	1.149, -0.832

	9a	9b
**CCDC-Number**	2490072	2490073
Chemical formula	$[C_{106}H_{112}Al_2N_6Si_8B_2F_{40}]_2$	$[C_{108}H_{116}Al_2N_6Si_8B_2F_{40}]_2$
M_r	5060.32	5116.72
Crystal system, space group	triclinic, $P\bar{1}$	triclinic, $P\bar{1}$
Temperature (K)	100	100
a (Å), α(°)<br>*b (Å)*, β(°)<br>*c (Å)*, γ(°)	13.2328(6), 90.990(2)<br>15.2365(8), 94.530(2)<br>29.6005(17), 100.736(2)	17.215(3), 85.030(6)<br>18.316(3), 89.205(5)<br>19.122(3), 82.255(5)
V (Å^3)	5842.2(5)	5952.0(18)
Z	1	1
F(000)	2596	2628
D_x (g/cm^3)	1.438	1.428
Radiation type	Mo $K\alpha$	Mo $K\alpha$
μ (mm^{-1})	0.220	0.217
θ range (°) for cell meas.	2.32–25.56	2.25–24.85
Crystal size (mm)	0.292 × 0.192 × 0.156	0.173 × 0.128 × 0.067
Diffractometer	Bruker Photon CMOS	Bruker Photon CMOS
Radiation source	TXS rotating anode	TXS rotating anode
Monochromator	Helios optic	Helios optic
Absorption correction	Multi-scan	Multi-scan
T_{min}, T_{max}	0.702, 0.745	0.585, 0.745
θ_{max} (°)	25.720	25.872
Range of *h*, *k*, *l*	*h* = -16→16, *k* = -18→18, *l* = -36→36	*h* = -21→21, *k* = -22→22, *l* = -4→23
Refinement method	Full-matrix least-squares on F^2	Full-matrix least-squares on F^2
Data/restraints/parameters	22193/16/1522	22766/551/1423
Goodness-of-fit on F^2	1.102	1.015
Final *R* indices (I>2σ(I))	R_1 = 0.0544, wR_2 = 0.1447	R_1 = 0.1161, wR_2 = 0.3421
$\Delta\rho_{max}$, $\Delta\rho_{min}$ (e Å^{-3})	2.078, -0.594	0.920, -0.813

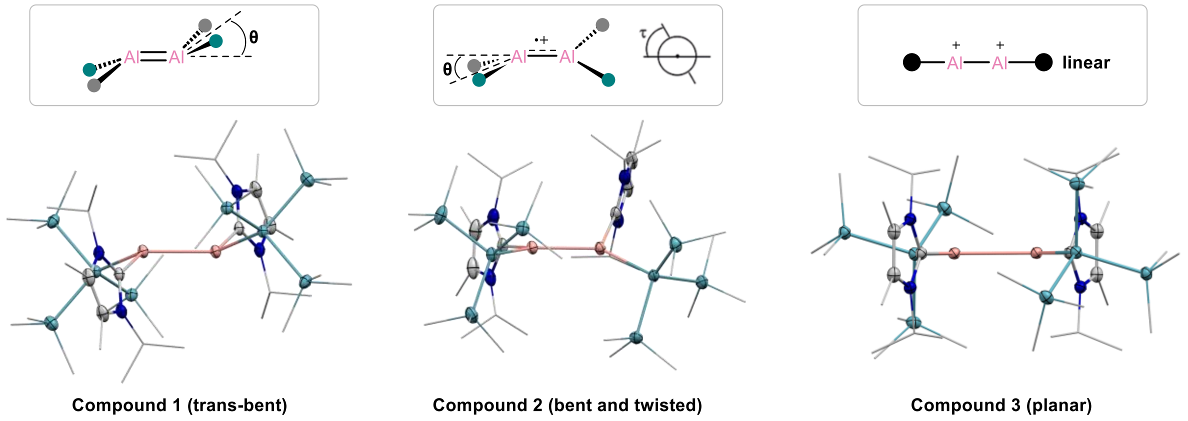

Supplementary Figure 47. Side-view depictions of the crystal structures of compounds **1-3**.

ORTEP-style illustrations

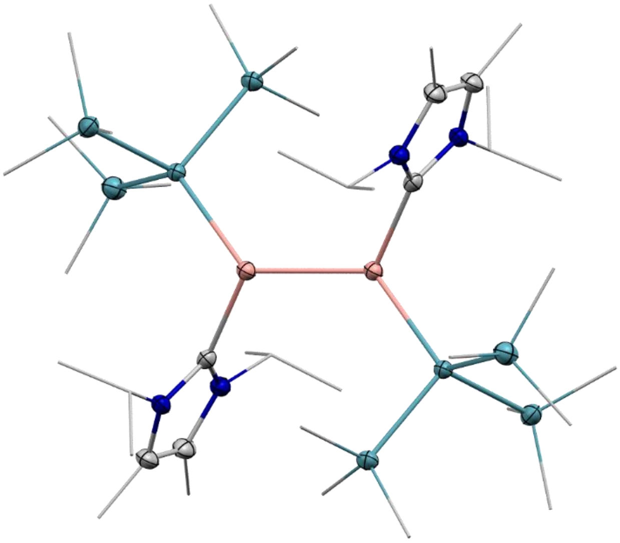

Supplementary Figure 48. Thermal ellipsoid plot (50% probability) of X-ray structure of compound **1** (anions were omitted for clarity).

CCDC: 2490063.

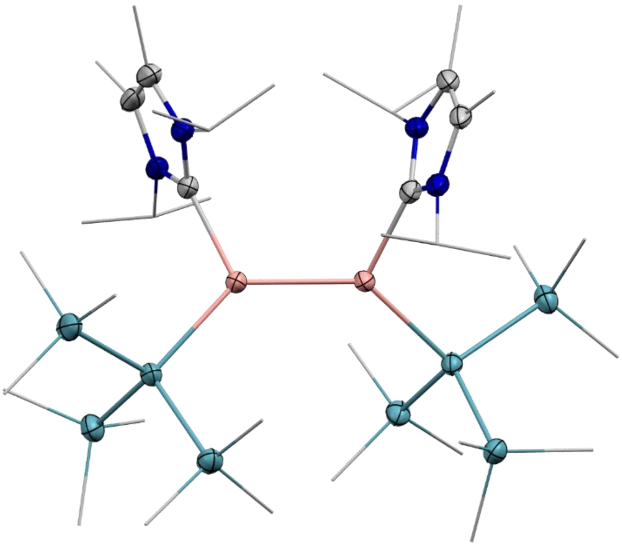

Supplementary Figure 49. Thermal ellipsoid plot (50% probability) of X-ray structure of compound **2** (anions were omitted for clarity).

CCDC: 2490064.

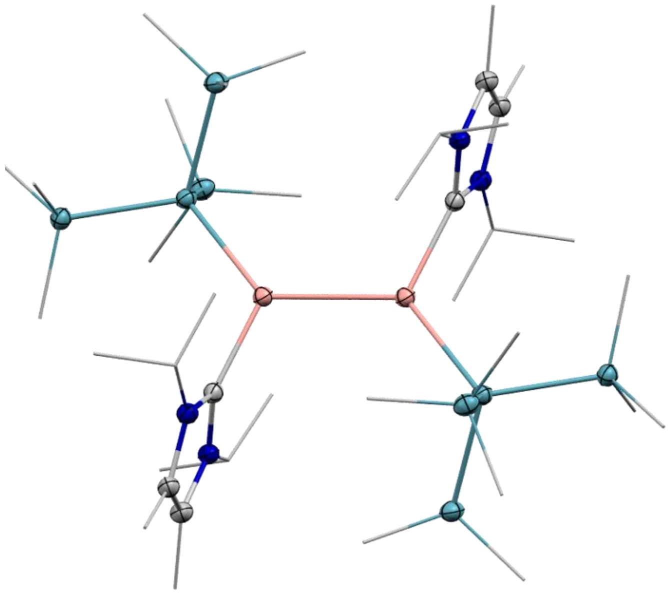

Supplementary Figure 50. Thermal ellipsoid plot (50% probability) of X-ray structure of compound **3** (anions were omitted for clarity).

CCDC: 2490065.

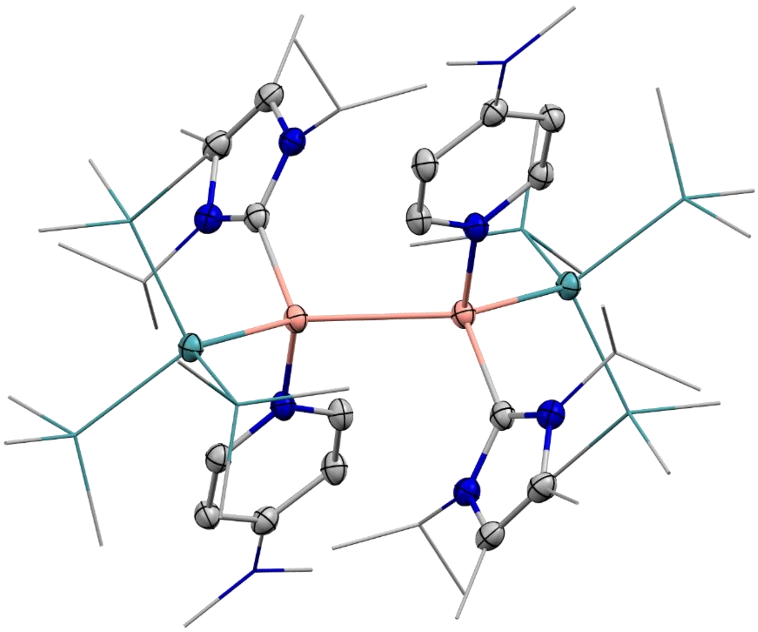

Supplementary Figure 51. Thermal ellipsoid plot (50% probability) of X-ray structure of compound **4a** (anions were omitted for clarity).

CCDC: 2490066.

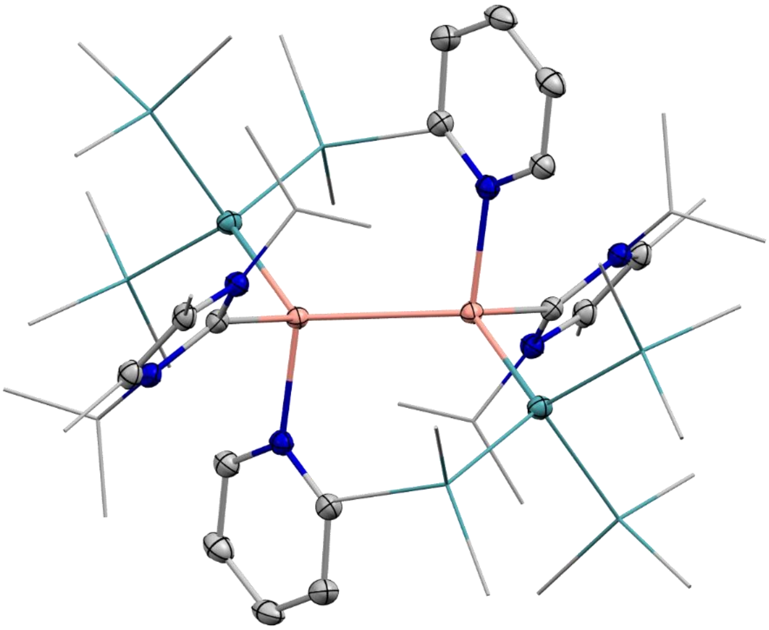

Supplementary Figure 52. Thermal ellipsoid plot (50% probability) of X-ray structure of compound **4b** (anions were omitted for clarity).

CCDC: 2490067.

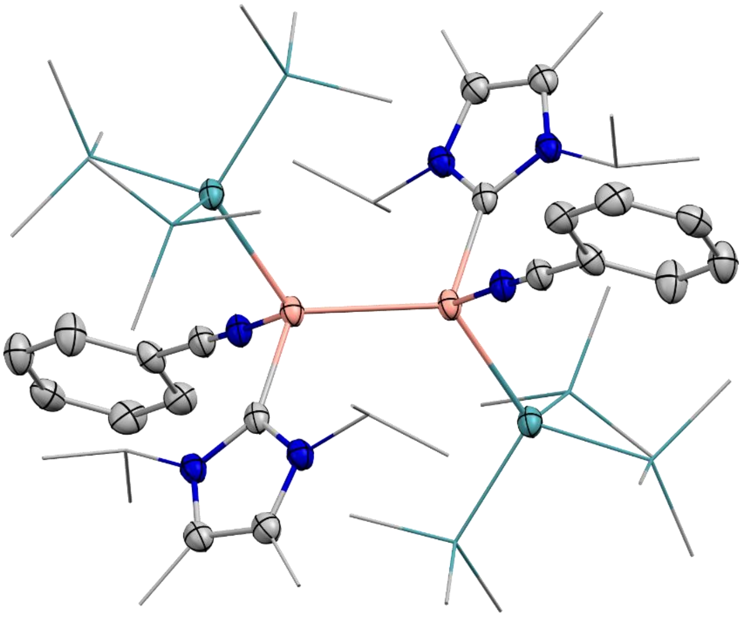

Supplementary Figure 53. Thermal ellipsoid plot (50% probability) of X-ray structure of compound **5** (anions were omitted for clarity).

CCDC: 2490068.

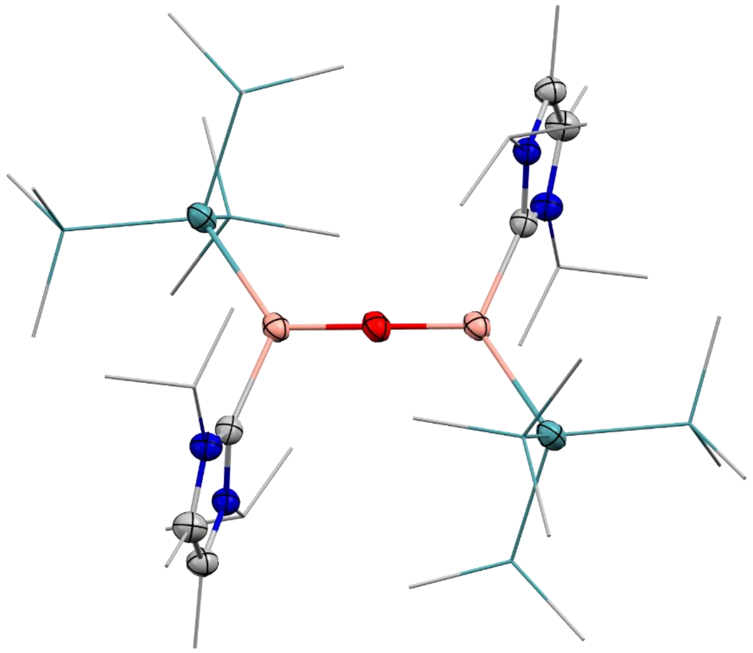

Supplementary Figure 54. Thermal ellipsoid plot (50% probability) of X-ray structure of compound **6** (anions were omitted for clarity).

CCDC: 2490069.

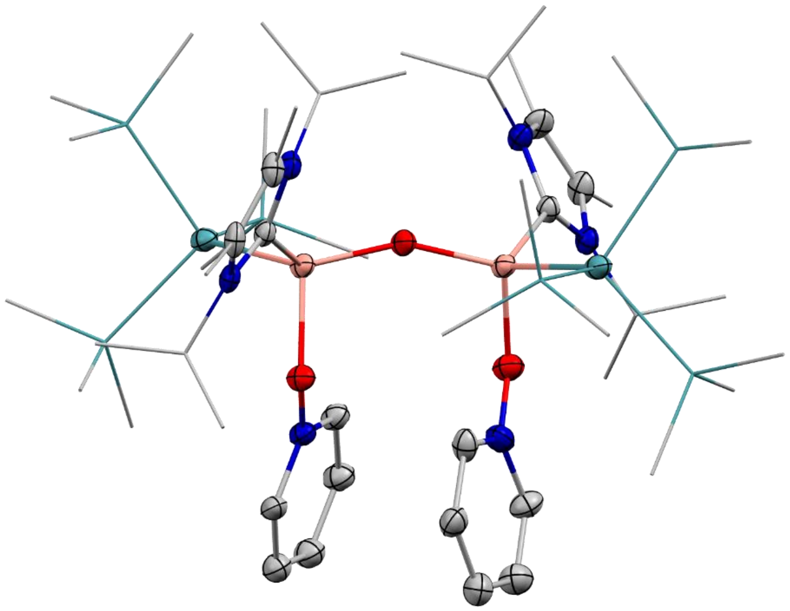

Supplementary Figure 55. Thermal ellipsoid plot (50% probability) of X-ray structure of compound **7** (anions were omitted for clarity).

CCDC: 2490070.

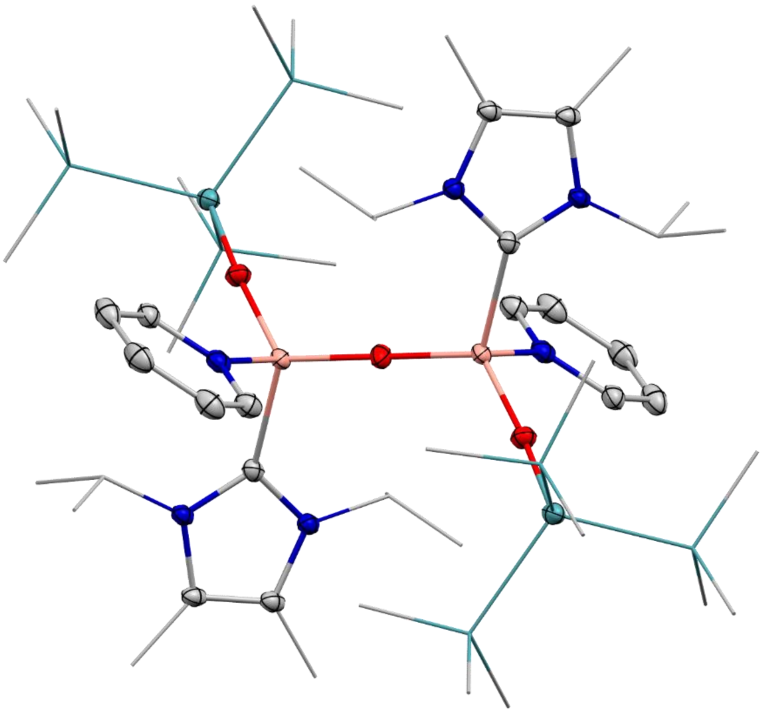

Supplementary Figure 56. Thermal ellipsoid plot (50% probability) of X-ray structure of compound **8** (anions were omitted for clarity).

CCDC: 2490071.

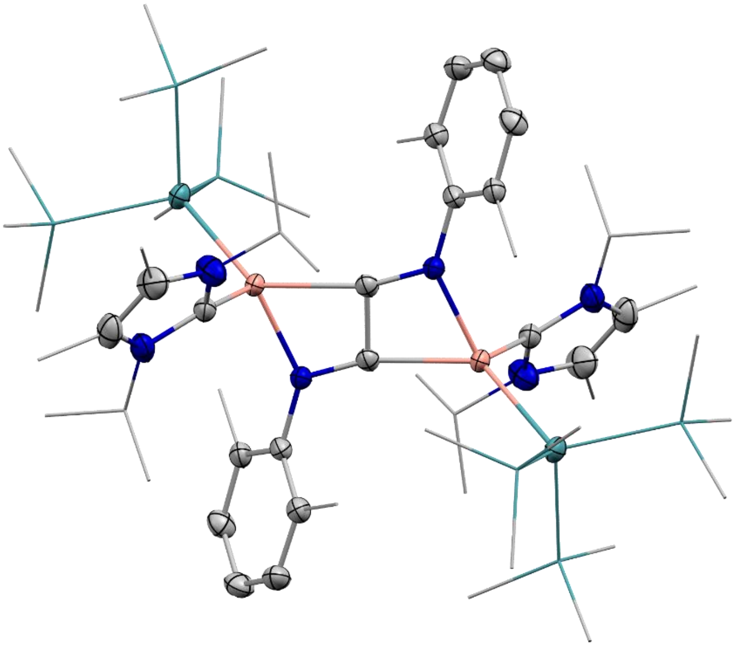

Supplementary Figure 57. Thermal ellipsoid plot (50% probability) of X-ray structure of compound **9a** (anions were omitted for clarity).

CCDC: 2490072.

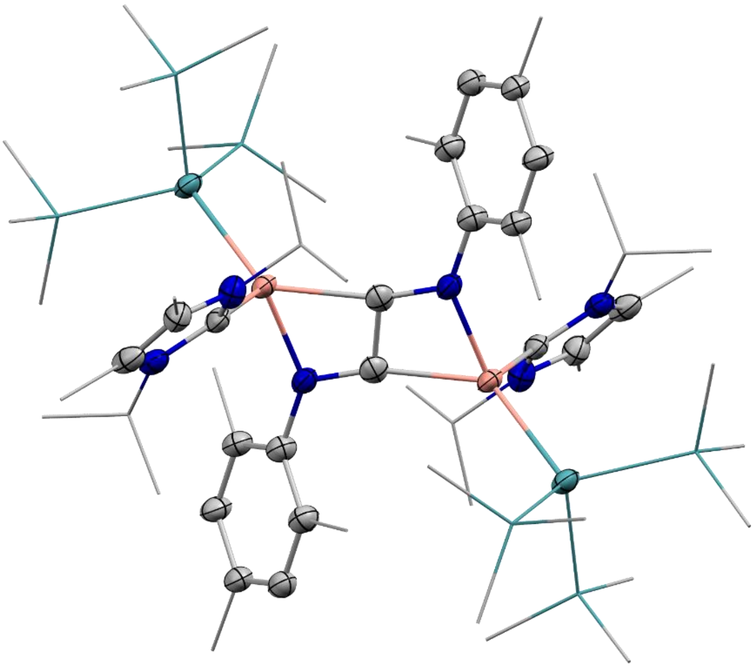

Supplementary Figure 58. Thermal ellipsoid plot (30% probability) of X-ray structure of compound **9b** (anions were omitted for clarity).

CCDC: 2490073.

3 Computational Details

Calculations were carried out using ORCA 6 software.[11-18] Geometry optimizations were carried using the B97-c composite method.[19] The optimized geometries were verified as minima by analytical frequency calculations. The NBO analysis was done using the NBO7 software,[20] at the PBE0[21]/def2-TZVP[22]//B97-c level of theory. TD-DFT calculations were carried out at the the B3LYP[23,24]/6-311G(2d,2p)[25–29]/SMD=THF//B97-c level of theory.

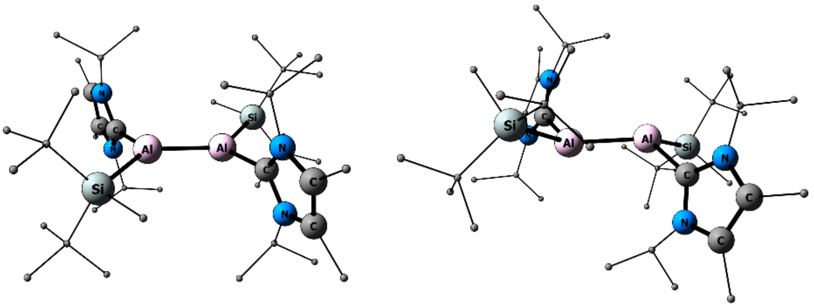

Supplementary Figure 59. Optimized structure of Si^tBu_2Me-substituted dialumene in trans-bent and trans-planar geometries.

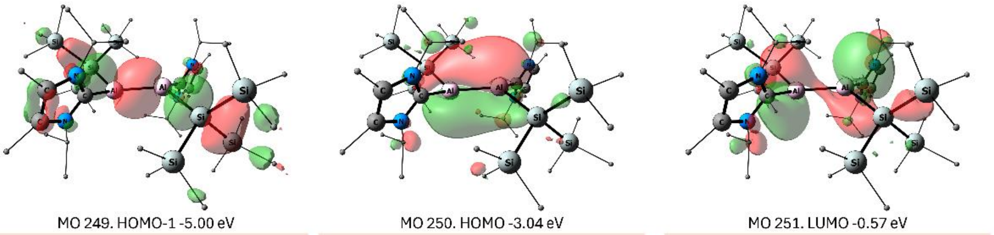

Bonding	N.B.	A.B.
94.3%	2.4%	3.3%

0.573*[123]	BD(1) Al9-Al10	32.8%
-0.437*[109]	BD(1) Si4-Al10	19.1%
-0.437*[122]	BD(1) Si8-Al9	19.1%

Bonding	N.B.	A.B.
87.4%	1.8%	10.9%

-0.894*[124]	BD(2) Al9-Al10	79.9%

Bonding	N.B.	A.B.
4.0%	5.5%	90.5%

-0.785*[278]	BD*(2) Al9-Al10	61.6%

Supplementary Figure 60. Selected canonical molecular orbitals (CMOs) of **1** and their NBO analysis.

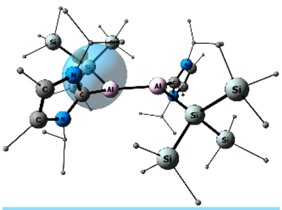

NBO 109. BD(1) Si4-Al10 occ. 1.93
70.08% Si4 sp1.91
29.92% Al10 sp2.30

Acceptor	E(2) (kcal mol^{-1})
284. BD*(2) N11-C32	3.3
262. BD*(1) Si3-C26	2.0
280. BD*(1) Al10-C32	1.5
276. BD*(1) Si8-Al9	1.4
252. BD*(1) Si1-C18	1.3

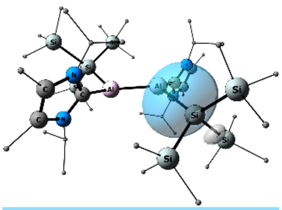

NBO 122. BD(1) Si8-Al9 occ. 1.93
70.08% Si8 sp1.91
29.92% Al9 sp2.30

Acceptor	E(2) (kcal mol^{-1})
291. BD*(2) N13-C52	3.3
275. BD*(1) Si7-C46	2.0
279. BD*(1) Al9-C52	1.5
263. BD*(1) Si4-Al10	1.4
265. BD*(1) Si5-C38	1.3

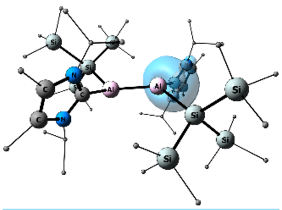

NBO 125. BD(1) Al9-C52 occ. 1.94
15.92% Al9 sp4.44
84.08% C52 sp1.23

Acceptor	E(2) (kcal mol^{-1})
294. BD*(1) N14-C53	6.0
288. BD*(1) N13-C48	5.8
280. BD*(1) Al10-C32	1.9
276. BD*(1) Si8-Al9	1.8
739. RY (3) N13	1.2

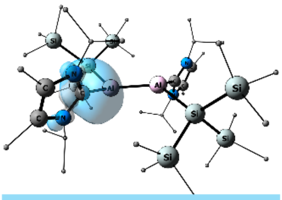

NBO 126. BD(1) Al10-C32 occ. 1.94
15.92% Al10 sp4.44
84.08% C32 sp1.23

Acceptor	E(2) (kcal mol^{-1})
287. BD*(1) N12-C33	6.0
281. BD*(1) N11-C28	5.8
279. BD*(1) Al9-C52	1.9
263. BD*(1) Si4-Al10	1.8
687. RY (3) N11	1.2

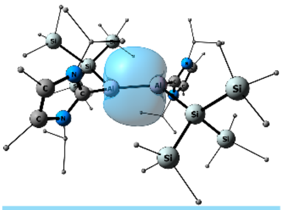

NBO 123. BD(1) Al9-Al10 occ. 1.91
50.00% Al9 sp1.16
50.00% Al10 sp1.16

Acceptor	E(2) (kcal mol^{-1})
278. BD*(2) Al9-Al10	3.3
284. BD*(2) N11-C32	2.8
291. BD*(2) N13-C52	2.8
286. BD*(1) N12-C32	1.0
293. BD*(1) N14-C52	1.0

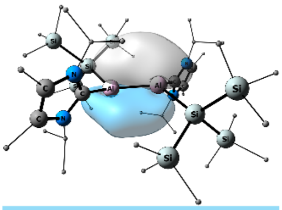

NBO 124. BD(2) Al9-Al10 occ. 1.75
50.00% Al9 sp17.42
50.00% Al10 sp17.41

Acceptor	E(2) (kcal mol^{-1})
277. BD*(1) Al9-Al10	6.7
303. BD*(1) C17-H61	4.8
358. BD*(1) C37-H108	4.8
397. BD*(1) C50-H142	2.7
342. BD*(1) C30-H95	2.6

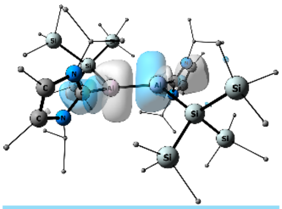

NBO 277. BD*(1) Al9-Al10 occ. 0.03
50.00% Al9 sp1.16
50.00% Al10 sp1.16

Donor	E(2) (kcal mol^{-1})
124. BD(2) Al9-Al10	6.7
125. BD(1) Al9-C52	0.8
126. BD(1) Al10-C32	0.8
109. BD(1) Si4-Al10	0.6
122. BD(1) Si8-Al9	0.6

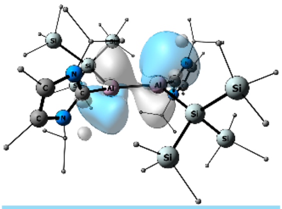

NBO 278. BD*(2) Al9-Al10 occ. 0.07
50.00% Al9 sp17.42
50.00% Al10 sp17.41

Donor	E(2) (kcal mol^{-1})
123. BD(1) Al9-Al10	3.3
149. BD(1) C17-H61	3.0
204. BD(1) C37-H108	3.0
188. BD(1) C30-H95	1.1
243. BD(1) C50-H142	1.1

Supplementary Figure 61. Selected NBOs of **1**, their atomic orbital compositions and five largest donor-acceptor interactions, according to the second-order perturbation theory.

Supplementary Table 2. TD-DFT calculated excites states (S1-S10) of **1** at the B3LYP/6-311G(2d,2p)/SMD=THF//B97-c level of theory.

STATE	Transitions	Contribution	fosc(D2)	E (cm^{-1})	λ (nm)
S_1	HOMO→LUMO+1	0.983913 (c= 0.99192403)	0.000000179	16584.9	603.0
S_2	HOMO-1→LUMO+3<br>HOMO→LUMO<br>HOMO→LUMO+2	0.010824 (c= -0.10403635)<br>**0.859625** (c= 0.92715958)<br>0.094799 (c= 0.30789414)	**0.514792359**	17643.9	566.8
S_3	HOMO→LUMO<br>HOMO→LUMO+2	0.089809 (c= 0.29968192)<br>**0.896767** (c= -0.94697768)	**0.097136646**	18981.6	526.8
S_4	HOMO→LUMO+3	0.977397 (c= -0.98863369)	0.000000128	19318.1	517.6
S_5	HOMO→LUMO+4<br>HOMO→LUMO+5	0.798572 (c= -0.89362834)<br>0.171284 (c= 0.41386443)	0.203363493	24896.6	401.7
S_6	HOMO→LUMO+4<br>HOMO→LUMO+5<br>HOMO→LUMO+7	0.149934 (c= 0.38721316)<br>0.786979 (c= 0.88711823)<br>0.038348 (c= -0.19582529)	0.031547997	25586.5	390.8
S_7	HOMO→LUMO+6<br>HOMO→LUMO+8<br>HOMO→LUMO+10	0.810363 (c= -0.90020140)<br>0.019143 (c= 0.13835913)<br>0.137893 (c= 0.37133996)	0.000000180	25852.5	386.8
S_8	HOMO→LUMO+4<br>HOMO→LUMO+5<br>HOMO→LUMO+7<br>HOMO→LUMO+9	0.032081 (c= -0.17911256)<br>0.022412 (c= -0.14970783)<br>0.843787 (c= -0.91857858)<br>0.087077 (c= 0.29508781)	0.037257603	26902.0	371.7
S_9	HOMO→LUMO+6<br>HOMO→LUMO+8<br>HOMO→LUMO+10<br>HOMO→ LUMO+12	0.128371 (c= -0.35828925)<br>0.466779 (c= -0.68321215)<br>0.345657 (c= -0.58792597)<br>0.030188 (c= -0.17374682)	0.000000157	27076.9	369.3
S_{10}	HOMO→LUMO+7<br>HOMO→LUMO+9	0.096444 (c= -0.31055455)<br>0.897534 (c= -0.94738253)	0.011640516	27545.1	363.0

Supplementary Table 3. TD-DFT calculated excites states (S1-S10) of **2** at the B3LYP/6-311G(2d,2p)/SMD=THF//B97-c level of theory.

STATE	Transitions	Contribution	fosc(D2)	E (cm^{-1})	λ (nm)
S_1	SOMOα → LUMOα<br>SOMOα → LUMOα+1	**0.957563** (c= -0.97855151)<br>0.014402 (c= 0.12000703)	**0.100115931**	10938.1	914.2
S_2	SOMOα → LUMOα<br>SOMOα → LUMOα+1<br>HOMOβ → LUMOβ	0.012054 (c= 0.10978884)<br>**0.964132** (c= 0.98190225)<br>0.013222 (c= -0.11498883)	**0.071560125**	16460.6	607.5
S_3	SOMOα → LUMOα+2<br>SOMOα → LUMOα+3	0.977163 (c= 0.98851568)<br>0.014244 (c= -0.11934891)	0.005764693	16999.7	588.2
S_4	SOMOα → LUMOα+1<br>HOMOβ → LUMOβ	0.011460 (c= 0.10705351)<br>0.948415 (c= 0.97386624)	0.014370940	19705.6	507.5
S_5	SOMOα → LUMOα+2<br>SOMOα → LUMOα+3	0.017253 (c= 0.13135245)<br>0.940174 (c= 0.96962555)	0.003995922	21004.8	476.1
S_6	SOMOα → LUMOα+4<br>SOMOα → LUMOα+15<br>HOMOβ → LUMOβ	0.947755 (c= -0.97352730)<br>0.014456 (c= -0.12023248)<br>0.010909 (c= 0.10444509)	0.051948713	22225.1	449.9
S_7	HOMOα-1 → LUMOα<br>HOMOβ-1 → LUMOβ<br>HOMOβ → LUMOβ+1	0.762258 (c= 0.87307408)<br>0.021203 (c= 0.14561260)<br>0.169487 (c= 0.41168813)	0.001215710	25248.4	396.1
S_8	SOMOα → LUMOα+5<br>SOMOα → LUMOα+7<br>SOMOα → LUMOα+9	0.923567 (c= -0.96102418)<br>0.024786 (c= -0.15743644)<br>0.018000 (c= -0.13416539)	0.049659289	26125.6	382.8
S_9	SOMOα → LUMOα+6<br>SOMOα → LUMOα+10<br>SOMOα → LUMOα+11<br>HOMOβ → LUMOβ+1	0.933397 (c= -0.96612460)<br>0.011766 (c= -0.10846906)<br>0.010467 (c= 0.10230887)<br>0.016613 (c= -0.12889107)	0.005778023	27367.9	365.4
S_{10}	SOMOα → LUMOα+5<br>SOMOα → LUMOα+7<br>SOMOα → LUMOα+12	0.031145 (c= -0.17647918)<br>0.933111 (c= 0.96597685)<br>0.019079 (c= -0.13812837)	0.003296019	27535.3	363.2

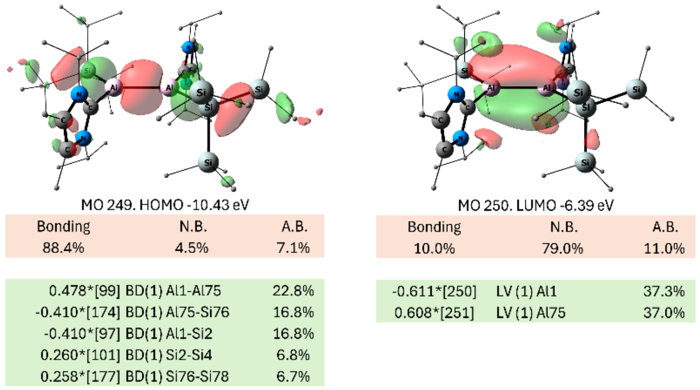

Supplementary Figure 62. Selected canonical molecular orbitals (CMOs) of **3** and their NBO analysis.

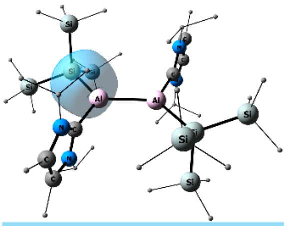

NBO 97. BD(1) Al1-Si2 occ. 1.94
33.92% Al1 sp1.49
66.08% Si2 sp2.53

Acceptor	E(2) (kcal mol^{-1})
253. BD*(1) Al1-C14	3.1
254. BD*(1) Al1-Al75	2.3
269. BD*(2) N6-C14	2.0
323. BD*(1) C67-H68	1.1
329. BD*(1) Al75-Si76	1.1

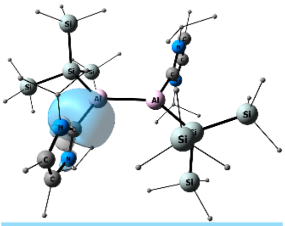

NBO 98. BD(1) Al1-C14 occ. 1.94
14.96% Al1 sp3.60
85.04% C14 sp1.35

Acceptor	E(2) (kcal mol^{-1})
272. BD*(1) N7-C24	5.6
270. BD*(1) N6-C15	5.4
252. BD*(1) Al1-Si2	3.2
330. BD*(1) Al75-C88	1.7
254. BD*(1) Al1-Al75	1.6

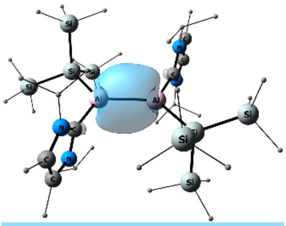

NBO 99. BD(1) Al1-Al75 occ. 1.89
50.01% Al1 sp1.61
49.99% Al75 sp1.61

Acceptor	E(2) (kcal mol^{-1})
345. BD*(2) N80-C88	4.6
269. BD*(2) N6-C14	4.6
252. BD*(1) Al1-Si2	4.3
329. BD*(1) Al75-Si76	4.3
1616. RY (1) C88	3.3

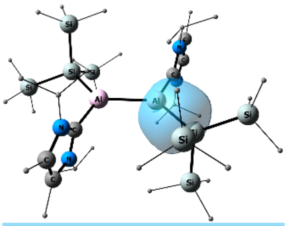

NBO 174. BD(1) Al75-Si76 occ. 1.94
33.92% Al75 sp1.49
66.08% Si76 sp2.54

Acceptor	E(2) (kcal mol^{-1})
330. BD*(1) Al75-C88	3.1
254. BD*(1) Al1-Al75	2.3
345. BD*(2) N80-C88	2.0
252. BD*(1) Al1-Si2	1.1
399. BD*(1) C141-H142	1.0

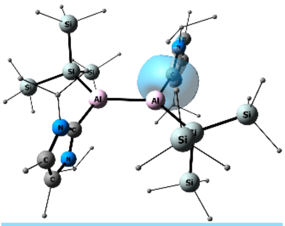

NBO 175. BD(1) Al75-C88 occ. 1.94
14.92% Al75 sp3.60
85.08% C88 sp1.34

Acceptor	E(2) (kcal mol^{-1})
348. BD*(1) N81-C98	5.6
346. BD*(1) N80-C89	5.5
329. BD*(1) Al75-Si76	3.1
253. BD*(1) Al1-C14	1.7
254. BD*(1) Al1-Al75	1.6

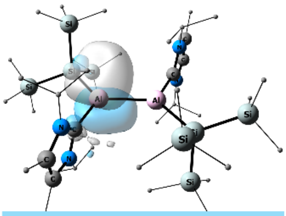

NBO 250. LV(1) Al1 occ. 0.15
sp1.00

Donor	E(2) (kcal mol^{-1})
168. BD(1) C67-H68	14.9
171. BD(1) C71-H72	11.2
173. BD(1) C71-H74	10.2
170. BD(1) C67-H70	5.4
102. BD(1) Si2-Si5	3.1

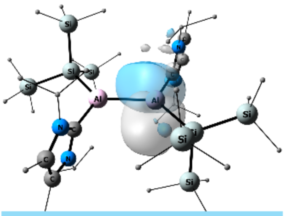

NBO 251. LV(1) Al75 occ. 0.15
sp1.00

Donor	E(2) (kcal mol^{-1})
244. BD(1) C141-H142	14.4
247. BD(1) C145-H146	11.7
249. BD(1) C145-H148	10.2
246. BD(1) C141-H144	5.4
178. BD(1) Si76-Si79	3.2

Supplementary Figure 63. Selected NBOs of **3**, their atomic orbital compositions and five largest donor-acceptor interactions, according to the second-order perturbation theory.

4 Supplementary References

1. Bill, E. EPR Program Eview. 2019.
2. Bill, E. EPR Program Esim. 2019.
3. Gaffney, B. J.; Silverstone, H. J. Simulation of the EMR Spectra of High-Spin Iron in Proteins; Springer, Boston, MA, 1993; pp 1–57. https://doi.org/10.1007/978-1-4615-2892-0_1.
4. APEX suite of crystallographic software, APEX 4 version 2021.10-0; Bruker AXS Inc.: Madison, Wisconsin, USA (2021).
5. SAINT, Version 7.56a and SADABS Version 2008/1; Bruker AXS Inc.: Madison, Wisconsin, USA (2008).
6. Sheldrick, G. M. SHELXL-2014, University of Göttingen, Göttingen, Germany (2014).
7. Hübschle, C. B.; Sheldrick, G. M.; Dittrich, B. ShelXle: A Qt Graphical User Interface for SHELXL. *J. Appl. Cryst.* **44**, 1281-1284 (2011).
8. Sheldrick, G. M. SHELXL-97, University of Göttingen, Göttingen, Germany (1998).
9. Wilson, A. J. C. International Tables for Crystallography, Vol. C, Tables 6.1.1.4 (pp. 500-502), 4.2.6.8 (pp. 219-222), and 4.2.4.2 (pp. 193-199); Kluwer Academic Publishers: Dordrecht, The Netherlands (1992).
10. Macrae, C. F.; Bruno, I. J.; Chisholm, J. A.; Edgington, P. R.; McCabe, P.; Pidcock, E.; Rodriguez-Monge, L.; Taylor, R.; van de Streek, J.; Wood, P. A. Mercury CSD 2.0 - new features for the visualization and investigation of crystal structures. *J. Appl. Cryst.* **41**, 466-470 (2008).
11. F. Neese, "Software update: The ORCA program system—Version 5.0" *WIREs Comput. Mol. Sci.***12***,* e1606 (2022).
12. F. Neese, "An improvement of the resolution of the identity approximation for the formation of the Coulomb matrix" *J. Comput. Chem.* **24**, 1740–1747 (2003).
13. D. Bykov, T. Petrenko, R. Izsák, S. Kossmann, U. Becker, E. Valeev, F. Neese, "Efficient implementation of the analytic second derivatives of Hartree–Fock and hybrid DFT energies: a detailed analysis of different approximations" *Mol. Phys.* **113**, 1961–1977 (2015).
14. F. Neese, "The <scp>SHARK</scp> integral generation and digestion system" *J. Comput. Chem.* **44**, 381–396 (2023).
15. S. Grimme, J. Antony, S. Ehrlich, H. Krieg, "A consistent and accurate ab initio parametrization of density functional dispersion correction (DFT-D) for the 94 elements H-Pu" *J. Chem. Phys.* **132**, DOI 10.1063/1.3382344/926936 (2010).
16. S. Grimme, S. Ehrlich, L. Goerigk, "Effect of the damping function in dispersion corrected density functional theory" *J. Comput. Chem.* **32**, 1456–1465 (2011).
17. F. Neese, "The ORCA program system" *Wiley Interdiscip. Rev. Comput. Mol. Sci.* **2**, 73–78 (2012).
18. F. Neese, F. Wennmohs, U. Becker, C. Riplinger, "The ORCA quantum chemistry program package" *J. Chem. Phys.* **152**, 224108 (2020).
19. J. G. Brandenburg, C. Bannwarth, A. Hansen, S. Grimme, "B97-3c: A revised low-cost variant of the B97-D density functional method" *J. Chem. Phys.* **148**, DOI 10.1063/1.5012601/196461 (2018).
20. NBO 7.0. E. D. Glendening, J, K. Badenhoop, A. E. Reed, J. E. Carpenter, J. A. Bohmann, C. M. Morales, P. Karafiloglou, C. R. Landis, and F. Weinhold, Theoretical Chemistry Institute, University of Wisconsin, Madison (2018). preprint.
21. C. Adamo, V. Barone, "Toward reliable density functional methods without adjustable parameters: The PBE0 model" *J. Chem. Phys.* **110**, 6158–6170 (1999).
22. F. Weigend, R. Ahlrichs, "Balanced basis sets of split valence, triple zeta valence and quadruple zeta valence quality for H to Rn: Design and assessment of accuracy" *Phys. Chem. Chem. Phys.* **7**, 3297–3305 (2005).

23. A. D. Becke, “Density-functional thermochemistry. III. The role of exact exchange” *J. Chem. Phys.* **98**, 5648–5652 (1993).
24. C. Lee, W. Yang, R. G. Parr, “Development of the Colle-Salvetti correlation-energy formula into a functional of the electron density” *Phys. Rev. B* **37**, 785–789 (1988).
25. T. Clark, J. Chandrasekhar, G. W. Spitznagel, P. V. R. Schleyer, “Efficient diffuse function-augmented basis sets for anion calculations. III. The 3-21+G basis set for first-row elements, Li–F” *J. Comput. Chem.* **4**, 294–301 (1983).
26. L. A. Curtiss, M. P. McGrath, J. P. Blaudeau, N. E. Davis, R. C. Binning, L. Radom, “Extension of Gaussian-2 theory to molecules containing third-row atoms Ga–Kr” *J. Chem. Phys.* **103**, 6104–6113 (1995).
27. J. P. Blaudeau, M. P. McGrath, L. A. Curtiss, L. Radom, “Extension of Gaussian-2 (G2) theory to molecules containing third-row atoms K and Ca” *J. Chem. Phys.* **107**, 5016–5021 (1997).
28. A. D. McLean, G. S. Chandler, “Contracted Gaussian basis sets for molecular calculations. I. Second row atoms, Z=11–18” *J. Chem. Phys.* **72**, 5639–5648 (1980).
29. R. Krishnan, J. S. Binkley, R. Seeger, J. A. Pople, “Self-consistent molecular orbital methods. XX. A basis set for correlated wave functions” *J. Chem. Phys*. **72**, 650–654 (1980).
